# Elucidation of the (*R*)-enantiospecific benzylisoquinoline alkaloid biosynthetic pathways in sacred lotus (*Nelumbo nucifera*)

**DOI:** 10.1038/s41598-023-29415-0

**Published:** 2023-02-20

**Authors:** Ivette M. Menéndez-Perdomo, Peter J. Facchini

**Affiliations:** Department of Biological Sciences, University of Calgary, Calgary, AB T2N 1N4 Canada

**Keywords:** Biochemistry, Chemical biology, Plant sciences

## Abstract

Benzylisoquinoline alkaloids (BIAs) are a structurally diverse group of plant specialized metabolites found mainly in members of the order Ranunculales, including opium poppy (*Papaver somniferum*), for which BIA biosynthetic pathways leading to the critical drugs morphine, noscapine, and sanguinarine have been elucidated. Sacred lotus (*Nelumbo nucifera*), in the order Proteales, accumulates medicinal BIAs in the proaporphine, aporphine, and bisbenzylisoquinoline structural subgroups with a prevalence of *R* enantiomers, opposed to the dominant *S* configuration occurring in the Ranunculales. Nevertheless, distinctive BIA biosynthetic routes in sacred lotus have not been explored. *In planta* labeling experiments and in vitro assays with recombinant enzymes and plant protein extracts showed that dopamine and 4-hydroxyphenylacetaldehyde derived from l-tyrosine serve as precursors for the formation of (*R,S*)-norcoclaurine in sacred lotus, whereas only (*R*)-norcoclaurine byproducts are favored in the plant by action of *R*-enantiospecific methyltransferases and cytochrome P450 oxidoreductases (CYPs). Enzymes responsible for the *R*-enantiospecific formation of proaporphine (NnCYP80Q1) and bisbenzylisoquinoline (NnCYP80Q2) scaffolds, and a methylenedioxy bridge introduction on aporphine substrates (NnCYP719A22) were identified, whereas additional aspects of the biosynthetic pathways leading to the distinctive alkaloid profile are discussed. This work expands the availability of molecular tools that can be deployed in synthetic biology platforms for the production of high-value alkaloids.

## Introduction

Sacred lotus (*Nelumbo nucifera* Gaertn.) is an aquatic plant of the Nelumbonaceae family, in the order Proteales. Sacred lotus has been extensively cultivated in parts of Asia as a food crop, for religious observance owing to the symbolic purity of the plant in both Buddhist and Hindu traditions, and due to numerous applications used for centuries in traditional Ayurvedic and Chinese medicine^1^. The medicinal properties of sacred lotus are primarily associated with the accumulation of several benzylisoquinoline alkaloids (BIAs), which include the core 1-benzylisoquinoline scaffold and derivatives belonging to the proaporphine, aporphine, and bisbenzylisoquinoline structural subgroups^2^. Major alkaloids, which accumulate in leaves and embryos, include norcoclaurine, a 1-benzylisoquinoline exhibiting anti-HIV activity^3^, pronuciferine, a proaporphine that shows promise as a neuroprotective agent in the treatment of Alzheimer’s disease^4^, nuciferine, an aporphine reportedly effective for the treatment of lung, colon, prostate, and breast cancers^5^, and neferine, a bisbenzylisoquinoline recently identified as a potential therapeutic agent to treat COVID-19^6^. Despite the pharmacological importance of sacred lotus alkaloids, limited research has been performed on the elucidation of corresponding BIA biosynthetic pathways.

Extensive investigation of BIA metabolic biochemistry in the opium poppy (*Papaver somniferum*) and related species in the order Ranunculales has resulted in the complete elucidation of biosynthetic pathways leading to several pharmacologically important alkaloids (Supplementary Fig. 1), including morphine (morphinan), noscapine (phthalideisoquinoline), and sanguinarine (benzophenanthridine)^7^. Despite the structural diversity represented in the various subclasses, all BIAs produced in members of the Ranunculales share a common biosynthetic origin beginning with the enantioselective Pictet-Spengler condensation of two l-tyrosine derivatives, dopamine and 4-hydroxyphenylacetaldehyde (4-HPAA), which is enzymatically catalyzed by norcoclaurine synthase (NSC) to yield exclusively (*S*)-norcoclaurine, the first committed intermediate in BIA metabolism^8–12^. (*S*)-Norcoclaurine is sequentially methylated by norcoclaurine 6-*O*-methyltransferase (6OMT) and coclaurine *N*-methyltransferase (CNMT) forming (*S*)-coclaurine and (*S*)-*N*-methylcoclaurine, respectively. Subsequently, the cytochrome P450 monooxygenase (CYP) *N*-methylcoclaurine 3′-hydroxylase (NMCH) produces (*S*)-3′-hydroxy-*N*-methylcoclaurine, which is further 4′-*O*-methylated by 3′-hydroxy-*N*-methylcoclaurine 4′-*O*-methyltransferase (4′OMT) yielding (*S*)-reticuline, the central branch-point intermediate leading to most BIAs in the Ranunculales. Various oxidative enzymes catalyzing specific C–C and C–O couplings convert (*S*)-reticuline to protoberberine (and subsequently derived benzophenanthridines and phthalideisoquinolines), aporphine, and morphinan alkaloids^7,13^.

The presumed monophyletic evolution of BIA biosynthesis in angiosperms^14^ suggests that opium poppy BIA metabolic pathways can be used to guide the selection of corresponding biosynthetic genes in BIA-accumulating species. Consequently, sacred lotus BIA biosynthesis is predicted to begin with the NCS-catalyzed condensation of dopamine and 4-HPAA, followed by conversion of (*R,S*)-norcoclaurine by specific enzymes in a limited number of families (e.g., OMT, NMT, and CYPs) to differentially substituted 1-benzylisoquinoline, proaporphine, aporphine, and bisbenzylisoquinoline alkaloids. Previously, we identified and functionally characterized genes encoding 6OMT (NnOMT1) and 7OMT (NnOMT5), and showed that these enzymes contribute to the diversity among 1-benzylisoquinolines in sacred lotus^2^. However, distinctive features of sacred lotus BIA metabolism remained unexplained. First, *N. nucifera* alkaloids occur primarily as *R*-enantiomers, contrary to the predominant occurrence of *S*-enantiomers in the Ranunculales. Moreover, although NCS catalyzes the enantioselective formation of (*S*)-norcoclaurine, both (*R*)- and (*S*)-norcoclaurine have been detected in sacred lotus^3,15,16^, indicating the potential involvement of either a non-enantioselective enzyme or two distinct *R*- and *S*-enantioselective NCS orthologs. Therefore, the occurrence of enantiospecific enzymes operating downstream of norcoclaurine and favoring the conversion of the *R*-enantiomers would explain the unique stereochemistry of BIAs in sacred lotus compared with the Ranunculales. Second, BIAs containing a 3′-hydroxyl in the benzyl moiety have not been detected, owing presumably to the absence of an NMCH enzyme, suggesting that *N*-methylcoclaurine serves as a central branch-point intermediate in the biosynthesis of proaporphine, aporphine, and bisbenzylisoquinoline alkaloids in sacred lotus as opposed to the reticuline-derived formation of morphinans, protoberberines, phthalideisoquinolines, benzophenanthridines, and aporphines in the Ranunculales^1,7^. Third, contrary to the C–C phenol coupling that results in the direct conversion of (*S*)-reticuline to aporphine alkaloids in the Ranunculales^17^, the occurrence of proaporphines in sacred lotus suggests the involvement of a distinct mechanism responsible for an indirect conversion of 1-benzylisoquinoline substrates lacking *ortho-ortho* or *ortho-para* substituents in the benzylic moiety to aporphines^18^. Sacred lotus aporphines distinctively lack benzylic moiety substitutions and have been proposed to derive via their corresponding proaporphines, a process that requires additional reduction, dehydration, and aromatic ring rearrangement^19^. Fourth, bisbenzylisoquinolines reported in sacred lotus occur as head-to-tail dimers, whereas only tail-to-tail couplings have been reported in the Ranunculales^20^.Both intramolecular C–C and intermolecular C–O couplings leading to aporphine and bisbenzylisoquinoline alkaloids, respectively, are catalyzed by members of the CYP80 family^17,20^. Finally, major aporphines in sacred lotus contain a methylenedioxy bridge on the isoquinoline moiety. In the Ranunculales, several methylenedioxy bridge-forming enzymes in the CYP719A subfamily have been exclusively implicated in the biosynthesis of protoberberine^21^, which do not occur in sacred lotus.

Herein, we demonstrate that BIA biosynthesis in sacred lotus begins with the condensation of l-tyrosine-derived dopamine and 4-HPAA, although an enzyme responsible for the formation of norcoclaurine was not detected suggesting a non-enzymatic Pictet-Spengler condensation resulting in the formation of racemic norcoclaurine. We show that (*S*)-norcoclaurine does not proceed to the major proaporphine, aporphine, and bisbenzylisoquinoline alkaloids in sacred lotus owing to the *R*-enantiospecific preference of early biosynthetic pathway enzymes. Three *R*-enantiospecific CYP enzymes, proaporphine synthase (NnCYP80Q1), bisbenzylisoquinoline synthase (NnCYP80Q2), and an aporphine methylenedioxy bridge-forming enzyme (NnCYP719A22), were identified and the predicted catalytic mechanisms are proposed. NMCH activity was not detected, explaining the lack of 3′-hydroxylation among sacred lotus BIAs and clarifying the role of *N*-methylcoclaurine as the central branch-point intermediate in the biosynthesis of alkaloids in this plant. In addition, the lack of detected activity associated with CNMT and 4′OMT candidates suggests a strict requirement for *R* enantiomers as substrates. Unfortunatey, key *R* enantiomeric compounds were not available for this study. Overall, our results reveal key aspects of the biosynthetic pathways leading to the distinctive alkaloid profile in sacred lotus, which also expands the availability of molecular tools that can be deployed in engineered microorganisms for the production of high-value benzylisoquinoline alkaloids.

## Results

### BIA biosynthetic gene candidates from sacred lotus

Supported by the availability of a *Nelumbo nucifera* draft genome^22,23^, transcripts encoding five NCS (NnNCS1, NnNCS3, NnNCS4, NnNCS5, and NnNCS7), three OMT (NnOMT6-8 in addition to NnOMT1-5 reported previously^2^), one CNMT (NnCNMT), and four CYP (NnCYP80P1, NnCYP80Q1, NnCYP80Q2, and NnCYP719A22) candidates were selected based on their amino acid sequence identity with gene orthologs from opium poppy, or using previously reported search results^24–26^ (Supplementary Table 1). Molecular masses and amino acid sequences of predicted translation products are provided in Supplementary Tables 2–7. Sacred lotus NCS candidates, which phylogenetic analysis placed in an independent branch, showed < 40% amino acid sequence identity with respect to functionally characterized NCS enzymes (Supplementary Fig. 2A). A comparison between NnNCS candidates and *Thalictrum flavum* norcoclaurine synthase (TfNCS)^12,27^ showed that residues involved in substrate binding were mostly absent in orthologs from sacred lotus, with only NnNCS5 containing the key D141 catalytic determinant (Supplementary Fig. 3). NnOMT7 displayed 85% amino sequence identity with respect to NnOMT5, which was previously characterized as a 7OMT^2^, and both proteins clustered with NnOMT8 in a branch separate from 6OMT, 7OMT, and 4′OMT variants involved in the *O*-methylation of 1-benzylisoquinolines (Supplementary Fig. 2B). Amino acid sequence analysis of sacred lotus OMT candidates with respect to the functionally and structurally characterized *T. flavum* 6-*O*-methyltransferase (Tf6OMT)^28^ revealed that NnOMT6 contained a critical deletion compromising the catalytic residues H256 and D257, and the substitution of E315 by methionine (Supplementary Fig. 4); thus, NnOMT6 characterization was not pursued further. Other than a D257N mutation in NnOMT8, catalytic determinants were conserved in all other NnOMT candidates. The single NnCNMT candidate showed 80% identity with *Stephania intermedia* CNMT1 (SiCNMT1) and both enzymes clustered with SiCNMT2 (Supplementary Fig. 2C). Notably, *S. intermedia* CNMTs have been correlated with the *N*-methylation of secondary and tertiary amines in (*R*)-1-benzylisoquinolines biosynthesis^29^. Key active site residues identified in *Coptis japonica* CNMT (CjCNMT)^30^ and involved in BIA binding and catalysis were conserved in NnCNMT (Supplementary Fig. 5).

NnCYP80Q1 and NnCYP80Q2 exhibit 80% amino acid sequence identity and share a phylogenetic clade with *C. japonica* corytuberine synthase (CjCYP80G2)^17^ and *Berberis stolonifera* berbamunine synthase (BsCYP80A1)^20^, which are the only functionally characterized aporphine and bisbenzylisoquinoline synthases, respectively. In contrast, NnCYP80P1 shared a clade with NMCH (CYP80B subfamily), although the 3′-hydroxylation of 1-benzylisoquinolines does not occur in sacred lotus (Supplementary Fig. 2D). Alignments of NnCYP80 candidates and orthologous BIA biosynthetic genes (Supplementary Fig. 6) showed that a consensus sequence [(A/G)GX(D/E)T(T/S)] purportedly involved in alkaloid substrate interaction and formation of the oxygen-binding pocket exhibited a relevant amino acid substitution whereby alanine (underlined) is found in 3'-hydroxylases (A296 in NnCYP80P1) whereas proline occurs in aporphine and bisbenzylisoquinoline synthases (P288 in NnCYP80Q1 and P289 in NnCYP80Q2). Notably, NnCYP80P1 contained a 13-amino acid deletion after residue K125. Finally, NnCYP719A22 clustered with CYP719A enzymes annotated as canadine synthase, which incorporates a methylenedioxy bridge in the isoquinoline moiety of protoberberines^31,32^ (Supplementary Fig. 2E). For NnCYP719A22, the above consensus sequence showed characteristic amino acid substitutions occurring in other members of the CYP719A subfamily (Supplementary Fig. 7), whereby leucine (L293) replaces A/G (underlined) and serine (S297) substitutes threonine (underlined), the latter of which has been proposed to act in activation of the heme oxygen^33^. In addition, conserved residues associated with substrate binding were substituted in NnCYP719A22, including a tyrosine substituted by phenylalanine (F291), and leucine or aspartic acid residues replaced by threonine (T292). Moreover, the conserved motif [KPIAPXXXPH] displayed three unique substitutions (I358V, I362V, and deletion of alanine). A C-terminal cysteine involved in heme iron-binding was conserved in all CYP candidates.

### Organ-specific expression of BIA biosynthetic gene candidates from sacred lotus

**Figure 1:**
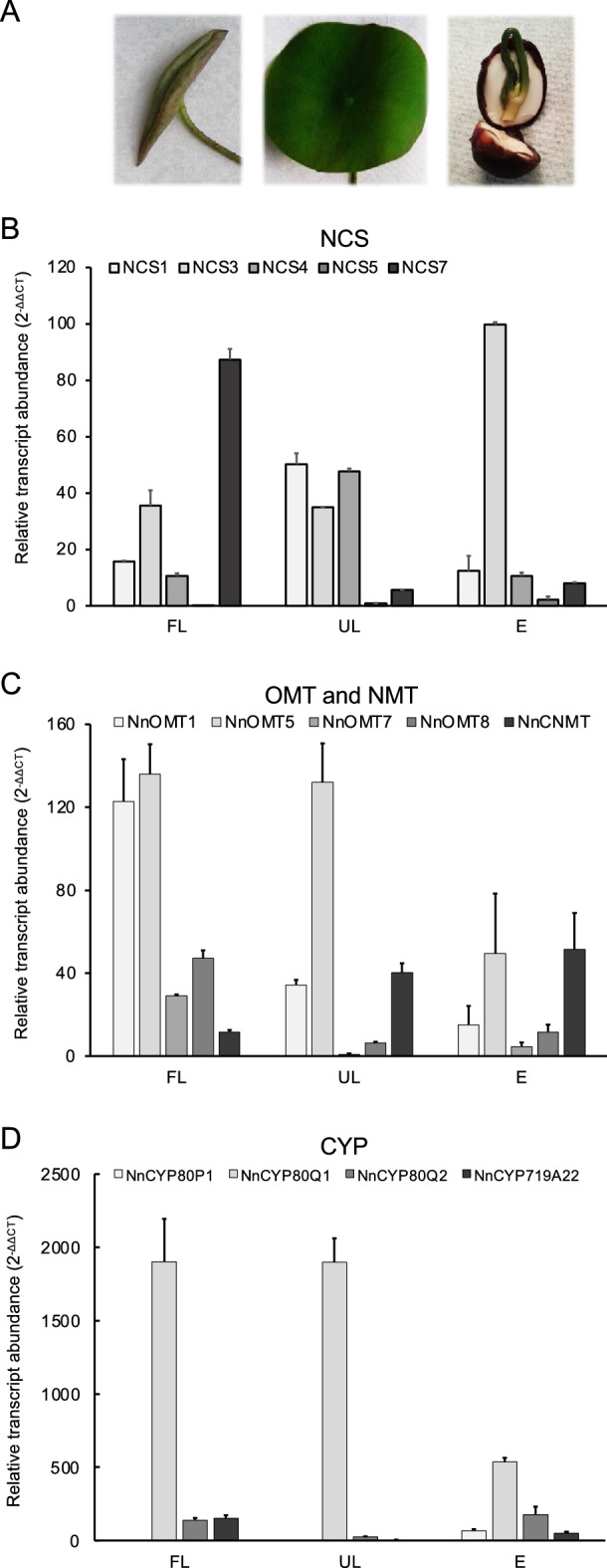
Organ-specific transcript levels in sacred lotus. (**A**) Images of a scared lotus folded leaf (FL), an unfolded leaf (UL), and an embryo (E) are shown. (**B–D**) Relative abundance of candidate BIA biosynthetic genes transcripts potentially encoding (**B**) norcoclaurine synthase (NCS), (**C**) *O*-methyltransferases (OMTs) and *N*-methyltransferase (NMT), and (**D**) cytochrome P450 monooxygenases (CYPs) in sacred lotus folded leaf, unfolded leaf, and embryos. Bars represent the mean ± standard deviation of three independent measurements.

Levels of transcript corresponding to each gene candidate were measured in sacred lotus leaves and embryos, organs in which BIAs primarily accumulate (Fig. 1A). NnNCS7 and NnNCS3 transcripts were most abundant in leaves and embryos, respectively, whereas NnNCS5 transcripts were almost absent (Fig. 1B). NnOMT7 and NnOMT8 transcript levels were highest in folded leaves and detected in unfolded leaves, but were 2–5 times lower than NnOMT1 and NnOMT5. In contrast, NnCNMT transcript levels increased with leaf growth and accumulated to a similar extent in unfolded leaves and embryos (Fig. 1C). NnCYP80Q1 displayed the highest CYP transcript levels in all organs, whereas NnCYP80P1 transcripts were low in leaves (Fig. 1D).

### Dopamine and 4-HPAA are BIA precursors

**Figure 2:**
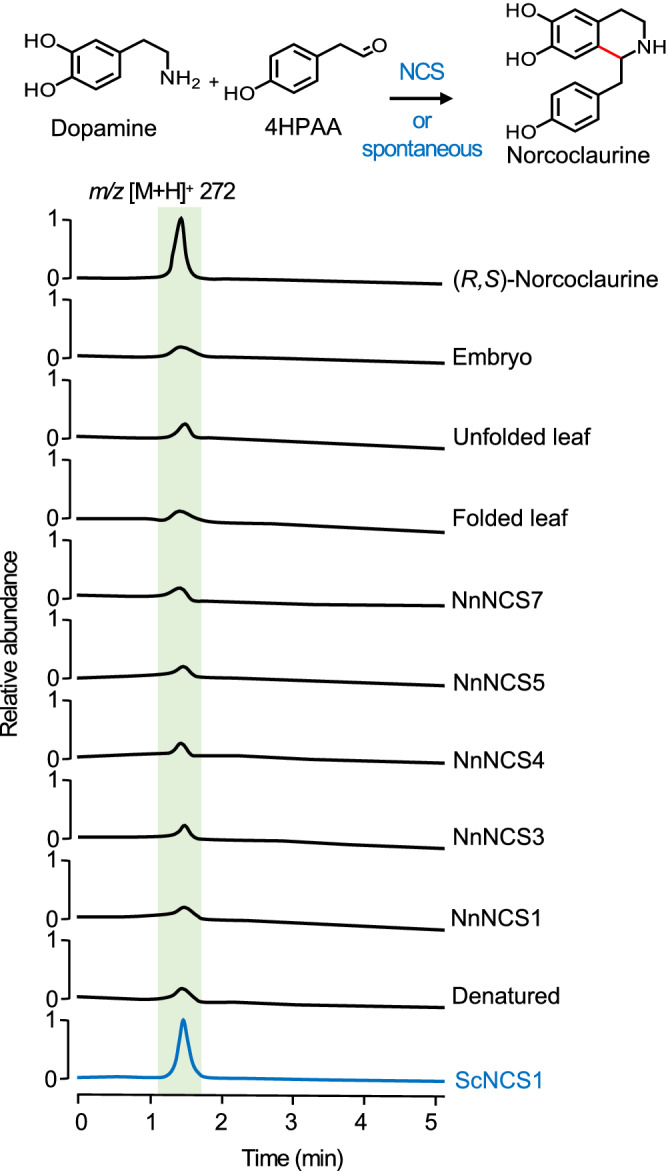
In vitro screening of recombinant NnNCS candidates and plant proteins extracts for norcoclaurine synthase activity. Extract-ion chromatograms show the relative formation of norcoclaurine (*m/z* [M + H]^+^ 272) from dopamine and 4-HPAA. Authentic (*R,S*)-norcoclaurine was used for retention time and mass spectrometric data comparisons. *Sanguinaria canadensis* NCS1 (in blue) was used as a positive control for the enzyme-catalyzed reaction. Spontaneous, non-enzymatic condensation was detected using denatured ScNCS1 protein as a negative control.

NCS, OMT, and CNMT candidates were produced in *Escherichia coli* as His_6_-tagged recombinant proteins, purified using cobalt-affinity chromatography, and detected by immunoblot analysis using an anti-His_6_ antibody (Supplementary Fig. 8). Purified NnOMT8 could not be recovered, precluding its further characterization. Using functionally characterized *Sanguinaria canadensis* NCS1^11^ as a positive control, recombinant NCS candidates and crude protein extracts from sacred lotus leaves and embryos were incubated with dopamine and 4-HPAA and assayed for norcoclaurine production. However, only ScNCS1 catalyzed the formation of norcoclaurine beyond levels produced via spontaneous condensation (Fig. 2). Since characterized NCS enzymes accept a relatively broad range of aldehyde substrates we tested phenylacetaldehyde (PAA)^34,35^ as an alternative substrate based on the hypothesis that the 4′-deoxynorcoclaurine reaction product could be subsequently 4′-hydroxylated to norcoclaurine in sacred lotus. Incubation of dopamine and PAA with ScNCS1 yielded 4′-deoxynorcoclaurine; however, no activity was detected for sacred lotus NCS candidates or plant protein extracts (Supplementary Fig. 9 and Supplementary Table 8).

**Figure 3:**
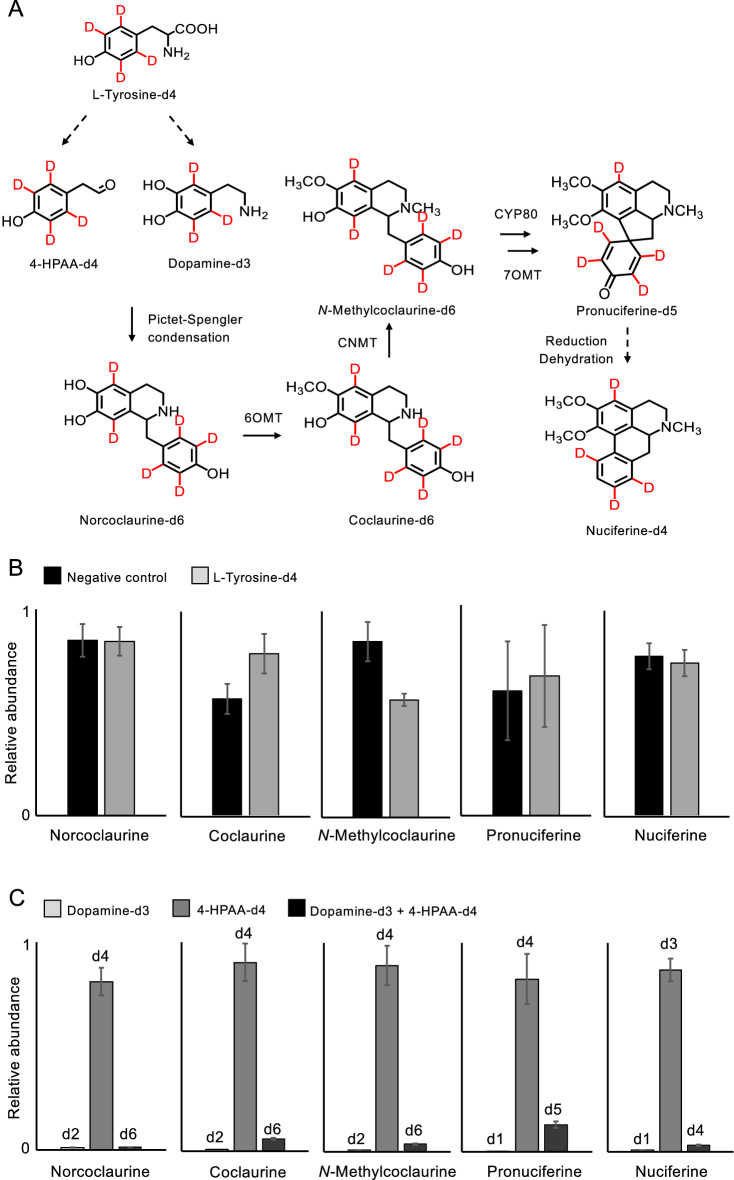
Incorporation of deuterated l-tyrosine into major alkaloids in sacred lotus folded leaves. (**A**) Labeling scheme from l-tyrosine-d4 to nuciferine-d4. Deuterated dopamine-d3 and 4-HPAA-d4 condensed to form norcoclaurine-d6. Subsequent 6-*O*-methylation and *N*-methylation yielded coclaurine-d6 and *N*-methylcoclaurine-d6, respectively. Sequential proaporphine formation and 7-*O*-methylation produced pronuciferine-d5, which was converted to nuciferine-d4 after reduction, dehydration, and aromatic ring rearrangement. The red deuterium (D) atoms show the labeled positions in each structure. Dashed arrows indicate more than one reaction. Other possible intermediates resulting from diverse combinations of labeled and non-labeled dopamine and 4-HPAA have been omitted for clarity (e.g., norcoclaurine-d2 resulting from dopamine-d3 and native 4-HPAA condensation or norcoclaurine-d4 resulting from native dopamine and 4-HPAA-d4 condensation). Graphs represent the relative abundance of (**B**) naturally occurring alkaloids in the negative control and in L-tyrosine-d4 fed plants and (**C**) deuterated alkaloids derived from (i) dopamine-d3 and native 4-HPAA, (ii) 4-HPAA-d4 and native dopamine, or (iii) dopamine-d3 and 4-HPAA-d4. Values represent the mean ± standard deviation of three independent measurements. Abbreviations: 4-HPAA, 4-hydroxyphenylacetaldehyde; CNMT, coclaurine *N*-methyltransferase; CYP80, cytochrome P450 monooxygenase; NCS, norcoclaurine synthase; OMT, *O*-methyltransferase.

To assess whether dopamine, 4-HPAA or PAA were precursors to BIA biosynthesis in sacred lotus, young leaves were incubated with either deuterated tyrosine (l-tyrosine-d4) or phenylalanine (l-phenylalanine-d5) and incorporation of the labeled amino acids into the plant alkaloids was monitored (Fig. 3, Supplementary Fig. 10, and Supplementary Table 9). Native alkaloids in control and treated leaves were equivalent (Fig. 3B and Supplementary Fig. 10B). Analysis of deuterated alkaloids in the l-tyrosine-d4 treatment revealed the presence of norcoclaurine-d6, resulting from the condensation of dopamine-d3 and 4-HPAA-d4. This result is consistent with the expected loss of two deuterium atoms during the five reactions necessary to convert two molecules of l-tyrosine to norcoclaurine, specifically, the hydroxylation of tyramine to dopamine, and the condensation of dopamine and 4-HPAA. In addition, the *O*- and *N*-methylated norcoclaurine-d6 derivatives, coclaurine-d6 and *N*-methylcoclaurine-d6, along with the major leaf proaporphine and aporphine alkaloids, pronuciferine-d5 and nuciferine-d4, were also detected (Fig. 3C and Supplementary Fig. 10C). Alkaloids were produced through a combination of deuterated and native dopamine and 4-HPAA, with a prevalence for products resulting from the coupling of native dopamine and 4-HPAA-d4. No deuterated alkaloids were detected in the negative control or from L-phenylalanine-d5.

### Methyltransferases’ substrate specificity in sacred lotus

**Figure 4:**
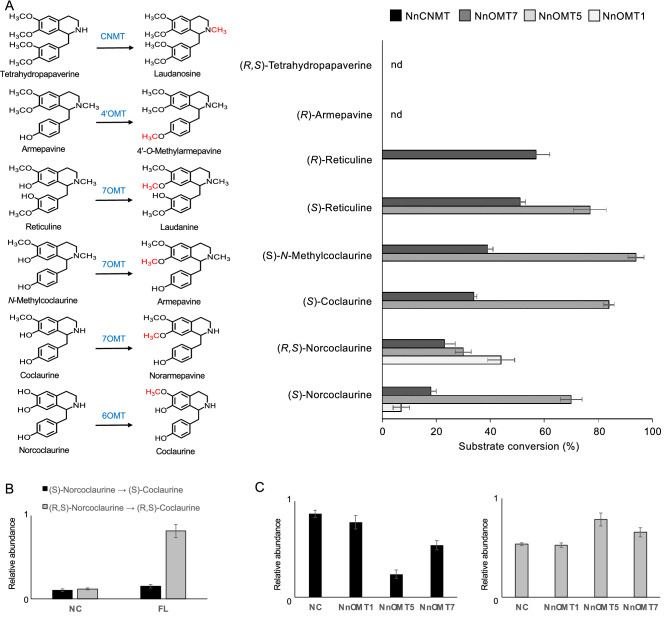
Activity of *O*-methyltransferases (NnOMTs) and *N*-methyltransferase (NnCNMT) candidates on potential substrates. (**A**) Substrates, predicted reaction products, and relative substrate conversion rates of recombinant NnCNMT, NnOMT1, NnOMT5, and NnOMT7. Reaction products corresponding to the only possible methylations of (*R*)-armepavine and (*R,S*)-tetrahydropapaverine were not detected. (**B**) Relative formation of coclaurine (6OMT activity) in crude protein extract from sacred lotus folded leaf incubated with (*S*)-norcoclaurine (black bars) versus (*R,S*)-norcoclaurine (gray bars). (**C**) Conversion of norcoclaurine (black bars) into coclaurine (gray bars) by NnOMT1, NnOMT5, and NnOMT7 incubated with a folded leaf alkaloid extract. Bars represent the mean ± standard deviation of three independent measurements. Abbreviations: FL, folded leaf; NC, negative control; nd, not detected.

Recombinant NnOMT7 or NnCNMT were incubated with SAM and various 1-benzylisoquinoline alkaloids as potential substrates (Fig. 4A and Supplementary Fig. 11). Functionally characterized NnOMT1 (6OMT) and NnOMT5 (7OMT) were assayed for comparison. NnOMT7 catalyzed the 6-*O*-methylation of norcoclaurine (*m/z* [M + H]^+^ 272) to coclaurine (*m/z* [M + H]^+^ 286) and the 7-*O*-methylation of coclaurine, *N*-methylcoclaurine (*m/z* [M + H]^+^ 300), and reticuline (*m/z* [M + H]^+^ 330) to norarmepavine (*m/z* [M + H]^+^ 300), armepavine (*m/z* [M + H]^+^ 314), and laudanine (*m/z* [M + H]^+^ 344), respectively (Supplementary Table 8). Recombinant NnOMT7 showed a pH optimum of 8.0 and maximum catalytic activity at 30 °C (Supplementary Fig. 12), a *K*_m_ value for (*R,S*)-norcoclaurine (6OMT activity) of 143 ± 30 µM, which is three-fold higher than the *K*_m_ for (*S*)-*N*-methylcoclaurine (7OMT activity) of 48 ± 4 µM. The catalytic efficiency (k_cat_/K_m_) for (*S*)-*N*-methylcoclaurine was substantially higher than for (*R,S*)-norcoclaurine (Supplementary Table 10).

*N*-Methyltransferase and 4′-*O*-methyltransferase activities were not detected for any sacred lotus recombinant enzyme candidate (Fig. 4A and Supplementary Fig. 11) or in plant crude protein extracts^2^, using any available substrate. Longer assay times also failed to yield *N*-methylated or 4′-*O*-methylated alkaloids (Supplementary Fig. 13), but evidenced the 7-*O*-methylation of (*R*)-reticuline by NnOMT5, which was not detected using shorter incubation. Interestingly, NnOMT1 activity with racemic norcoclaurine was higher compared with (*S*)-norcoclaurine, which was not tested in our previous study^2^, suggesting a preference for (*R*)-norcoclaurine (Fig. 4A and Supplementary Fig. 13). The preferential consumption of (*R,S*)-norcoclaurine versus (*S*)-norcoclaurine was further tested using plant protein extracts (Fig. 4B and Supplementary Fig. 14). Substantially more coclaurine was formed from (*R,S*)-norcoclaurine as opposed to (*S*)-norcoclaurine, suggesting that the sacred lotus 6OMT favored (*R*)-coclaurine formation. Moreover, the addition of non-enantiospecific NnOMT5 and NnOMT7 recombinant proteins to sacred lotus alkaloid extracts resulted in a decrease in endogenous norcoclaurine and an increase in coclaurine levels, whereas no substantial changes were detected using the *R*-enantiospecific NnOMT1 (Fig. 4C and Supplementary Fig. 15), indicating a specific accumulation of (*S*)-norcoclaurine in the plant, as opposed to (*R*)-norcoclaurine, likely owing to the latter being effectively used in downstream metabolic conversions.

### Novel CYPs involved in proaporphine, bisbenzylisoquinoline, and methylenedioxy bridge formation in sacred lotus

**Figure 5:**
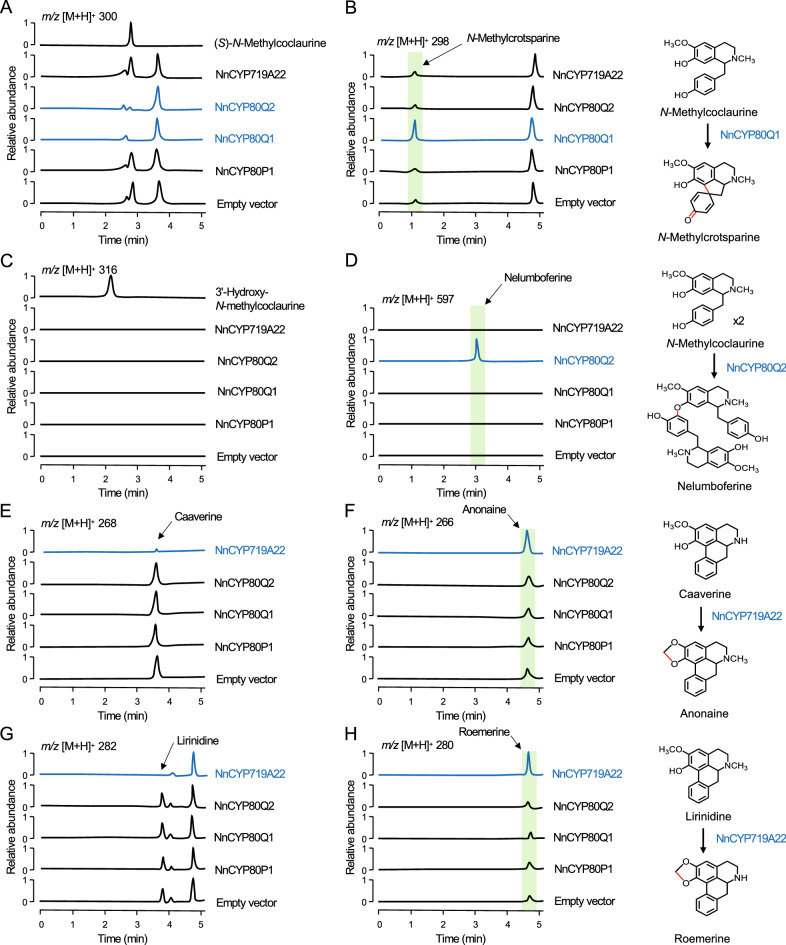
In vitro activity of sacred lotus cytochrome P450 (CYP) candidates in engineered yeast. Extracted-ion chromatograms show changes in the relative abundance of specific alkaloids in sacred lotus extracts after incubation with engineered yeast strains expressing the indicated NnCYP candidates. (**A**) Conversion of *N*-methylcoclaurine into the corresponding (**B**) proaporphine via intramolecular C–C phenol coupling by NnCYP80Q1, and (**D**) bisbenzylisoquinoline via intermolecular C–O phenol coupling by NnCYP80Q2. No 3′-hydroxy-*N*-methylcoclaurine reaction products were detected (**C**). Incorporation of a methylenedioxy bridge into the aporphines caaverine (**E**) and lirinidine (**G**) yielding anonaine (**F**) and roemerine (**H**), respectively, by NnCYP719A22.

Based on the alkaloid profile of sacred lotus, three CYP-catalyzed reactions were predicted: (1) the intramolecular C–C phenol coupling between the C8 and C1′ in 1-benzylisoquinoline substrates forming the corresponding proaporphines; (2) the intermolecular head-to-tail C–O phenol coupling between the C7 hydroxyl and C3′ of two 1-benzylisoquinoline substrates yielding the corresponding bisbenzylisoquinolines; and (3) the oxidative cyclization of *ortho* hydroxy and methoxy substituted aromatic rings in the isoquinoline moiety of aporphine substrates resulting in methylenedioxy bridge formation. All three conversions are oxidative reactions rather than typical CYP-catalyzed monooxygenations^36^. 3′-Hydroxylation of *N*-methylcoclaurine, ubiquitously catalyzed by NMCH in the Ranunculales, is not predicted to occur in sacred lotus. Four CYP candidate genes (*NnCYP80P1*, *NnCYP80Q1*, *NnCYP80Q2*, and *NnCYP719A22*) were independently expressed in a *Saccharomyces cerevisiae* strain containing one copy of opium poppy CYP reductase 2 (*PsCPR2*) and BIA uptake permease 1 (*PsBUP1*) genes integrated into the yeast genome (Supplementary Table 11). Initially, each yeast strain was incubated with (*S*)-*N*-methylcoclaurine and monitored for the formation of proaporphine (*m/z* [M + H]^+^ 298), bisbenzylisoquinoline (*m/z* [M + H]^+^ 597), and 3′-hydroxylated (*m/z* [M + H]^+^ 316) products; however, no conversions were detected (Supplementary Fig. 16), suggesting a potential strict requirement for (*R*)-*N*-methylcoclaurine as the substrate. Previously, we showed that *N*-methylcoclaurine is abundant in the folded leaves of sacred lotus^2^; thus, the yeast strains were incubated with an alkaloid extract prepared from sacred lotus folded leaves (Fig. 5). Endogenous *N*-methylcoclaurine was exclusively and almost completely depleted when the plant alkaloid extracts were exposed to yeast strains expressing *NnCYP80Q1* and *NnCYP80Q2*, and new peaks with *m/z* [M + H]^+^ 298 and *m/z* [M + H]^+^ 597, respectively, were detected. Collision-induced dissociation (CID) spectra for these two peaks were consistent with proaporphine and bisbenzylisoquinoline scaffolds, respectively (Supplementary Fig. 17 and Supplementary Table 8). The CID spectrum corresponding to the proaporphine *N*-methylcrotsparine (also known as glaziovine), showed a canonical peak equivalent to the loss of methylamine (*m/z* 255), which is consistent with the previously reported fragmentation of pronuciferine, a related proaporphine^37^. Interestingly, an additional peak at *m/z* 298 was consistently detected in all strains incubated with lotus alkaloid extract, which might correspond to the *N*-methylcrotsparine structural isomers, *N*-methylcrotonosine (C7–CH_3_ and N–CH_3_) or stepharine (C6–CH_3_ and C7–CH_3_), found in the plant alkaloid extract. Authentic standards were not available for these compounds, which precluded further characterization. Similarly, the observed CID spectrum for nelumboferine (*m/z* 597), a bisbenzylisoquinoline derived from *N*-methylcoclaurine, was in agreement with a previously reported spectrum showing major peaks at *m/z* 554, 298, and 192, which correspond to the loss of methylamine from one *N*-methylcoclaurine molecule, rupture of the C–O bond between both *N*-methylcoclaurine subunits, and cleavage of the C–C bond between the isoquinoline and benzyl moiety of one *N*-methylcoclaurine molecule, respectively^37,38^. The 3′-hydroxylation of *N*-methylcoclaurine was not detected using any of the yeast strains (Fig. 5).

Although the yeast strain expressing *NnCYP719A22* did not accept the endogenous *N*-methylcoclaurine in sacred lotus alkaloid extract, a substantial increase was detected in signals corresponding to *m/z* [M + H]^+^ 266 and *m/z* [M + H]^+^ 280, consistent with the methylenedioxy bridge-containing aporphines anonaine and roemerine, respectively (Fig. 5). The increased abundance of anonaine and roemerine was accompanied by a sharp decrease in the predicted substrates, *m/z* [M + H]^+^ 268 (caaverine) and *m/z* [M + H]^+^ 282 (lirinidine), respectively. The corresponding CID spectra were consistent with the reported fragmentation of anonaine and roemerine^2^, with the base peak matching the loss of (methyl)amine at *m/z* 249 and a lack of fragments below *m/z* 200 owing to the conjugated structure of aporphines^38^ (Supplementary Fig. 17 and Supplementary Table 8).

## Discussion

**Figure 6:**
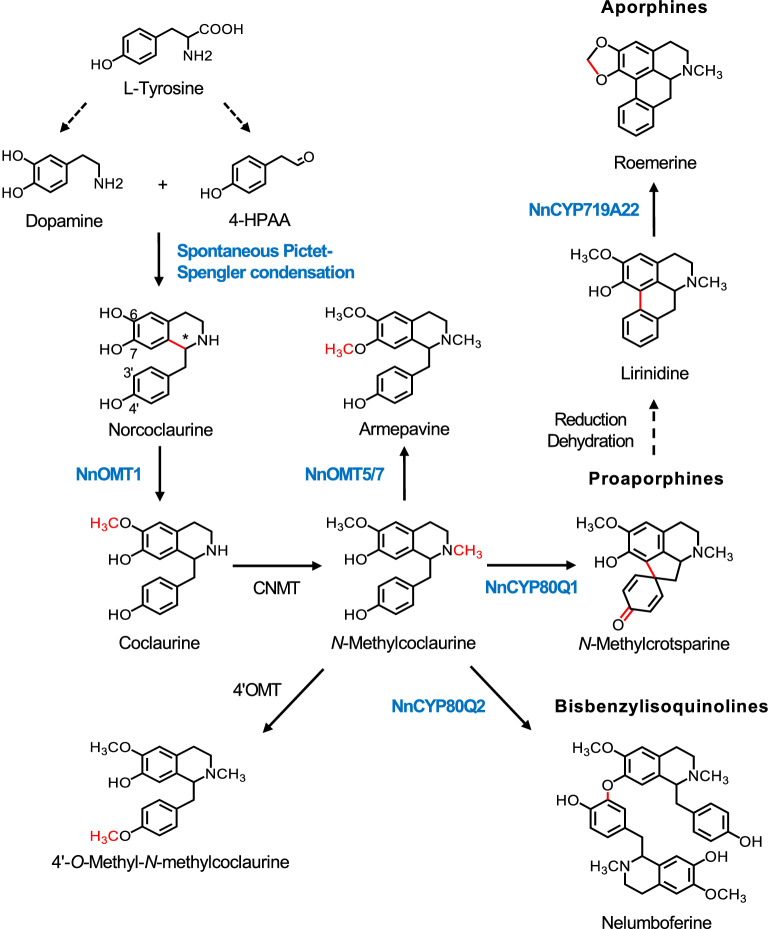
Proposed metabolic conversion of l-tyrosine to benzylisoquinoline alkaloids in sacred lotus. Dopamine and 4-HPAA condense spontaneously to form norcoclaurine. A 6-*O*-methyltransferase (NnOMT1) converts norcoclaurine to coclaurine, which is subsequently *N*-methylated to *N*-methylcoclaurine, the branch-point intermediate in the formation of three major alkaloid subgroups: bisbenzylisoquinolines, proaporphines, and aporphines. 1-Benzylisoquinolines could be *O*-methylated by NnOMT5 and NnOMT7 (7OMTs) or by an unknown 4′OMT or undergo intermolecular (bisbenzylisoquinolines) or intramolecular (proaporphines) phenol coupling by NnCYP80Q2 and NnCYP80Q1, respectively. Aporphines are generated from the corresponding proaporphine intermediates via reduction, dehydration, and rearrangement of the aromatic ring. CYP719A22 introduces a methylenedioxy bridge in the isoquinoline moiety of aporphine substrates, such as lirinidine. Other 1-benzylisoquinolines are likely converted to their corresponding aporphines and bisbenzylisoquinolines via parallel pathways, yielding the diversity of BIAs found in sacred lotus. Functionally characterized enzymes are shown in bold. Red functional groups in each structure indicates specific chemical conversions. Dashed arrows imply multiple enzymatic steps. Asterisk denotes the chiral center in norcoclaurine, and numbers refer to carbons. Alkaloids stereochemistry has been omitted for simplicity. Abbreviations: 4HPAA, 4-hydroxyphenylacetaldehyde; CNMT, coclaurine *N*-methyltransferase; CYP, cytochrome P450 monooxygenase; OMT, *O*-methyltransferase.

We present a revision of the proposed biosynthetic pathways leading to the major alkaloids in sacred lotus (Fig. 6). In vivo deuterium labeling experiments unambiguously showed that l-tyrosine-derived dopamine and 4-HPAA condense in the plant to form norcoclaurine. The occurrence in sacred lotus of both *R* and *S* enantiomers of norcoclaurine^3,15,16^, together with the absence of detectable NCS activity associated with candidate homologs, or even in plant protein extracts, strongly suggests a spontaneous, non-enzymatic Pictet-Spengler condensation of dopamine and 4-HPAA, in contrast to the enzymatic and enantioselective reaction yielding exclusively (*S*)-norcoclaurine in the Ranunculales^9–12^. Analysis of NnNCS amino acid sequences revealed that the key catalytic residue D141 was only conserved in NnNCS5, which showed exceptionally low transcript levels in the plant. The D141 residue is responsible for the initial deprotonation of the nitrogen atom in dopamine, triggering a nucleophilic attack on the 4-HPAA aldehyde and leading to the formation of an iminium intermediate^12^. D141E and D141N mutations resulted in a substantial (50–90%) decrease in NCS activity^39^. Furthermore, the highly conserved F99 residue and amino acids 76–80, forming the active site entrance loop, which have been proposed to favor a conformation change in the iminium intermediate required for the cyclization step^27^, were generally not found in NnNCS candidates. The absence of key catalytic and substrate-binding residues in sacred lotus NCS candidates is consistent with the determined lack of function.

We cannot entirely rule out the possibility that a different enzyme with unique reaction requirements is responsible for the Pictet-Spengler condensation of dopamine and 4-HPAA in sacred lotus. A limited number of gateway enzymes catalyzing Pictet-Spengler condensations have been reported in plant alkaloid metabolism^40^, including strictosidine synthase in the biosynthesis of monoterpenoid indole alkaloids and deacetylipecoside synthase in the formation of tetrahydroisoquinoline monoterpene glucosides. However, like NCS, both of these enzymes are enantioselective, which is inconsistent with the formation of both (*R*)- and (*S*)-norcoclaurine in sacred lotus. It could also be postulated that an *S*-enantioselective enzyme catalyzes the formation of (*S*)-norcoclaurine in sacred lotus, with second enzymes required for stereochemical inversion to (*R*)-norcoclaurine. However, the only known enzyme able to catalyze an *S*-to-*R* conversion in BIA biosynthesis is reticuline epimerase (REPI), which only accepts *N*-methylated substrates^41^ and is the outcome of a relatively rare gene-clustering event that appears to have occurred only in the genus *Papaver*, and resulted in the translational fusion of the two required oxidative and reductive enzymes^42,43^. However, dopamine and 4-HPAA efficiently yield racemic norcoclaurine under mild, non-enzymatic conditions, including physiological pH and temperature^9,44,45^. A similar non-enzymatic reaction has been reported for iminium formation in betalain biosynthesis via the spontaneous condensation of dopamine and betalamic acid^46^. Interestingly, racemic norcoclaurine has been detected in several plant taxa, including gymnosperms (*Gnetum* spp.), magnoliids (*Annona squamosa*), and Ranunculales (*Aconite* spp.)^47^, suggesting that the non-enantioselective, spontaneous condensation of dopamine and 4-HPAA might not be restricted to sacred lotus. In addition, bisbenzylisoquinoline alkaloids derived from (*R*)-*N*-methylcoclaurine have been reported in *B. stolonifera*^20^, suggesting that the spontaneous formation of racemic norcoclaurine might also occur parallel to NCS-catalyzed production of (*S*)-norcoclaurine.

Norcoclaurine undergoes several regiospecific SAM-dependent methylations to produce a variety of 1-benzylisoquinolines. In our previous functional characterization of sacred lotus OMTs, we isolated the genes encoding 6OMT and 7OMT^2^. Herein, we searched for enzymes responsible for the 4′-*O*-methylation and *N*-methylation of 1-benzylisoquinoline substrates by testing the homologs NnOMT7 and NnCNMT. NnOMT7 was assayed with various 1-benzylisoquinoline substrates and kinetically characterized using (*R,S*)-norcoclaurine and (*S*)-*N*-methylcoclaurine to confirm primarily 7-*O*-methyltransferase activity; thus it exhibits properties similar to NnOMT5^2^, in agreement with the considerable sequence identity (85%). The catalytic efficiency (k_cat_/K_m_) of NnOMT7 with (*S*)-*N*-methylcoclaurine (451 M^−1^ s^−1^) was substantially lower than the value previously determined for NnOMT5 (1465 M^−1^ s^−1^), whereas the K_m_ value (48 µM) of NnOMT7 was four-fold higher compared with NnOMT5 (13 µM)^2^. Together with the lower transcript levels of NnOMT7 in leaves and embryos, NnOMT5 is likely the main 7OMT in sacred lotus.

An enzyme capable of 4′-*O*-methylation on a 1-benzylisoquinoline was not detected. Secondary 4′-*O*-methyltransferase activity has been reported for some 6OMT and 7OMT enzymes, suggesting that 1-benzylisoquinolines can bind in the active site with a ‘flipped’ orientation^48^. Although our understanding of 4′OMT substrate recognition and enzymatic mechanics is incomplete, the catalytic determinants proposed for Tf6OMT^28^ are fully conserved in NnOMT1, NnOMT5, and NnOMT7. All characterized 4′OMT variants almost exclusively prefer 1-benzylisoquinoline substrates with a free hydroxyl group at C7^49–52^, which potentially explains why (*R*)-armepavine was not accepted (Fig. 4), and suggests that 4′-*O*-methylation occurs prior to 7-*O*-methylation in sacred lotus. However, norcoclaurine and coclaurine substrates have not been reported as substrates for 4′OMT from the Ranunculales, other than the minor activity of Cj4′OMT on (*R*)-coclaurine^50^. Since (*S*)-*N*-methylcoclaurine did not undergo 4′-*O*-methylation (Fig. 4) and only (*R*)-*N*-methylcoclaurine has been detected in the plant^3^, the latter is likely the preferred substrate for lotus 4′OMT. Similarly, *N*-methylation of available 1-benzylisoquinoline substrates was not detected for either the NnCNMT recombinant enzyme candidate or in plant protein extracts. Notably, NnCNMT displays 80% amino acid sequence identity with a recently characterized enzyme (SiCNMT1) that purportedly catalyzes the *N*-methylation of (*R*)-coclaurine^29^. Phylogenetic analysis showed that NnCNMT was also closely related to SiCNMT2, which catalyzes the single and double *N*-methylation of (*R*)-coclaurine yielding *N*-methylcoclaurine and magnocurarine (reported as isolotusine in sacred lotus), respectively^29^. Key active site residues involved in BIA-binding and catalysis have been determined for CjCNMT^30^, which favors (*R*)-coclaurine turnover in vitro^53^, and these positions are fully conserved in NnCNMT; thus, it is possible that NnCNMT strictly accepts (*R*)-coclaurine, but this substrate was not available to test.

Consistent with the prevalence of *R*-enantiomeric alkaloids in sacred lotus, catalytic activity was not detected when engineered yeast strains expressing CYP candidates were incubated with (*S*)-*N*-methylcoclaurine. However, replacing (*S*)-*N*-methylcoclaurine with sacred lotus leaf extracts resulted in detectable changes in the endogenous alkaloid profile for all CYP candidates other than NnCYP80P1. Phylogenetic analysis showed that NnCYP80P1 was closely related to regio- and stereo-specific 3′-hydroxylases involved in the formation of (*S*)-reticuline in the Ranunculales^54–57^. In agreement with previous reports^24,26^, NnCYP80P1 transcript levels were also negligible in the plant. The deletion of 13 amino acids in NnCYP80P1 potentially compromises correct protein folding (Supplementary Fig. 6). The absence of 1-benzylisoquinoline 3′-hydroxylase activity in sacred lotus contributes to a distinct plant alkaloid profile compared with members of the Ranunculales since several entry-point enzymes, such as salutaridine synthase^58^ (leading to morphinan alkaloids) and berberine bridge enzyme^59^ (leading to protoberberines, phthalideisoquinolines, and benzophenanthridines) exhibit catalytic mechanisms strictly dependent on 3′-hydroxylated substrates.

NnCYP80Q1 catalyzed the enantiospecific conversion of (*R*)-*N*-methylcoclaurine to proaporphine *N*-methylcrotsparine (Fig. 5), which could either be 7-*O*-methylated to pronuciferine, the most abundant proaporphine in the sacred lotus, or used in the formation of lirinidine and related aporphines (e.g., roemerine and nuciferine) (Supplementary Fig. 18). In vivo labeling in the plant also supported the formation of aporphines via 4′-hydroxylated proaporphines (Fig. 3). Although other 1-benzylisoquinolines with the required free C7–OH group, such as norcoclaurine and coclaurine, could also be considered direct proaporphine and aporphines precursors, in particular for those containing a secondary amine (e.g., asimilobine, *N*-nornuciferine, and anonaine), only the consumption of *N*-methylcoclaurine in plant alkaloid extracts was detected (Fig. 5). Aporphine synthase (CYP80G2) from *C. japonica* catalyzes the conversion of (*S*)-reticuline to (*S*)-corytuberine via direct C–C phenol coupling^17^. Interestingly, when incubated with (*S*)-*N*-methylcoclaurine, CYP80G2 yielded the corresponding proaporphine, although at significantly lower efficiency compared to the preferred reaction^17^, suggesting that aporphine and proaporphine synthesis functions are enzymatically conserved and depend on substrate availability (e.g., reticuline or *N*-methylcoclaurine). Based on the suggested formation of aporphines via a biradical reaction^17^, we propose a similar mechanism for sacred lotus proaporphine biosynthesis (Supplementary Fig. 19A). Aporphines derived from proaporphine intermediates must undergo further reduction, dehydration, and aromatic ring rearrangement^19^. Therefore, aporphine biosynthesis in sacred lotus requires an additional reductase, converting the proaporphine dienone to dienol, followed by an acid-catalyzed dienol-benzene rearrangement (Supplementary Fig. 19B). Although aldo–keto reductases and short-chain dehydrogenases/reductases have been implicated in BIA metabolism in the Ranunculales^60^, an equivalent enzyme involved in aporphine biosynthesis has not been reported. Since alkaloids with a dienol structure have not been detected in sacred lotus, the acid–base reaction might occur spontaneously.

NnCYP80Q2 catalyzed the head-to-tail formation of nelumboferine from (*R*)-*N*-methylcoclaurine (Fig. 5). Since feeding (*S*)-*N*-methylcoclaurine to the engineered yeast strain failed to yield a corresponding dimeric alkaloid, NnCYP80Q2 must require at least one molecule of (*R*)-*N*-methylcoclaurine. The major bisbenzylisoquinolines in sacred lotus have been detected in the (*R,R*)configuration^61^, although (*R,S*)-neferine and (*R,S*)-isoliensinine have also been reported^62^. Furthermore, the bisbenzylisoquinoline profile in sacred lotus can be rationalized as the outcome of dimerizing different combinations of *N*-methylcoclaurine, armepavine, and 4′-*O*-methyl-*N*-methylcoclaurine (Supplementary Fig. 20), although further *O*-methylation of nelumboferine scaffold can not be ruled out. However, consumption of other 1-benzylisoquinolines in plant alkaloid extracts or formation of corresponding bisbenzylisoquinoline alkaloids were not detected. Berbamunine synthase (CYP80A1) from *B. stolonifera*, the only characterized bisbenzylisoquinoline synthase^20,63^, catalyzes the tail-to-tail (C4′–O–C3′) phenol coupling of (1) (*S*)-coclaurine and (*R*)-*N*-methylcoclaurine, (2) (*R*)-*N*-methylcoclaurine and (*S*)-*N*-methylcoclaurine, and (3) two molecules of (*R*)-*N*-methylcoclaurine. Compared with CYP80A1, NnCYP80Q2 could bind its substrates with an alternative orientation favoring a head-to-tail coupling. Like NnCYP80Q2, CYP80A1 is unable to couple two molecules of (*S*)-*N*-methylcoclaurine and does not accept C3′ substituted 1-benzylisoquinolines likely due to the requirement of a free C3′ position to support the proposed biradical reaction mechanism^63^. Owing to the distinct C7–O–C3′ coupling regiospecificity, we propose a modified biradical reaction mechanism for NnCYP80Q2 (Supplementary Fig. 21).

NnCYP719A22 introduced a methylenedioxy bridge in the isoquinoline moiety of caaverine and lirinidine, yielding anonaine and roemerine, respectively (Fig. 5). The formation of a methylenedioxy bridge in an aporphine substrate has not been previously reported. Several characterized CYP719A enzymes incorporate a methylenedioxy bridge in the isoquinoline moiety of protoberberine alkaloids containing adjacent C6-OCH_3_ and C7-OH substitutions^21,64^. Previously, NnCYP719A22 was reported to not accept protoberberines^21^. Compared with CYP719A variants, NnCYP719A22 has several unique mutations (e.g., Y291F and L/D292T, V358I, V362I, and an alanine deletion) potentially related to its distinctive substrate preference. In the Ranunculales, methylenedioxy bridges are formed on protoberberines by oxidative cyclization of aromatic ring *ortho* hydroxy and methoxy groups; thus, a similar mechanism is expected for the action of NnCYP719A22 on aporphines (Supplementary Fig. 22).

BIAs are purported to provide defensive advantages to plants against herbivory and pathogen challenge, but the evolutionary forces behind the occurrence and maintenance of these specialized metabolites are not well understood^13^. In addition to the Ranunculales, BIAs occur sporadically in other plant orders including the Cornales, Laurales, Magnoliales, Piperales, Proteales, and Sapindales, although the BIA metabolism has not been investigated in members of these taxa^14^. A monophyletic evolution of BIA biosynthesis in angiosperms was proposed based on the purported detection of norcoclaurine synthase activity in total soluble protein extracts from 90 plant species. Mutations in key biosynthetic or regulatory genes affecting the conversion of norcoclaurine to more complex BIAs were suggested as a possible mechanism leading to inactivation of BIA metabolism in various taxa, explaining the scattered occurrence of alkaloids^14^. More recently, a phylogenomic study supported by genome sequence data for five BIA-accumulating species and assisted by remarkable progress in the isolation and characterization of a vast array of biosynthetic genes, indicated that BIA biosynthesis was of monophyletic origin in the Ranunculales but arose independently in the Proteales^42^. However, this conclusion was speculative since no sacred lotus BIA biosynthetic enzymes had yet been characterized. Herein, we demonstrate remarkable similarities between BIA biosynthesis in the Proteales and Ranunculales, and also striking differences, beginning with the non-enzymatic formation of racemic norcoclaurine in sacred lotus. In this regard, recruitment of NCS genes from a pathogenesis-related (PR)10 protein ancestor has been proposed as the watershed evolutionary event that facilitated BIA production in certain plant taxa^14,65^. In addition to NCS, two other PR10 proteins (i.e., thebaine synthase and neopinone isomerase) have been recruited into the morphinan alkaloid biosynthetic pathway in opium poppy^66,67^. Notably, all three PR10 proteins catalyze conversions that also proceed spontaneously, albeit less efficiently since factors such as enantioselectivity, labile byproduct formation and isomerization can be enzymatically controlled. One could also speculate that PR10 proteins represent relatively recent recruitment events to enhance yield since the metabolic processes can still proceed in their absence. Therefore, the emergence of an enantioselective NCS in the Ranunculales likely influenced the evolved enantiospecificity of downstream catalysts, although systematic evaluation using enantiomerically pure substrates has not been performed for most enzymes. Nevertheless, it is intriguing that the non-enantioselective spontaneous production of (*R,S*)-norcoclaurine is associated with the downstream metabolic transformation of only (*R*)-norcoclaurine. Our data suggests that (*S*)-norcoclaurine in sacred lotus is a metabolic dead-end from which no significant alkaloids are produced, in agreement with the vast prevalence of *R*-enantiomers among the major alkaloids in sacred lotus. Importantly, (*S*)-coclaurine and (*S*)-*N*-methylcoclaurine were not accepted as substrates by the sacred lotus CNMT candidate or by functionally characterized CYPs, respectively. The emergence of NCS, considered a relatively inefficient enzyme^39^, could nevertheless ‘economise’ plant resources by directing the formation of one enantiomer. The *S*-enantioselectivity of NCS has been related to the reaction energy profile, the preferred geometries of the intermediates, and transition states in the active site^12^. However, it is unknown if there are any underlying benefits to producing (*S*)-norcoclaurine for the plant, or if this outcome was serendipitous.

Conversely, a convergent evolution in BIA metabolism has been purported for the endogenous production of morphine in mammals^68^. Remarkably, plant and animal morphine biosynthetic pathways exhibit nearly identical chemistry, but utilize an entirely divergent set of enzymes (e.g., CYP719B1 versus CYP2D6 in opium poppy and humans, respectively, catalyze the formation of salutaridine). In mammals, spontaneous non-enzymatic Pictet-Spengler condensation of dopamine and 3,4-dihydroxyphenylacetaldehyde forming (*R,S*)-norlaudanosoline has been proposed and, although most biosynthetic enzymes remain unidentified, a human *R*-enantiospecific *N*-methyltransferase catalyzing the conversion of (*R*)-norreticuline to (*R*)-reticuline has been characterized^69^, supporting the prevalence of an *R*-enantiomeric route to reticuline. Both the prevalence of *R*-enantiospecific enzymes and the proposed spontaneous Pictet-Spengler condensation resemble features of BIA biosynthesis in sacred lotus, supporting the potential for repeated evolution of BIA metabolism.

Our work reveals unique features of BIA biosynthesis in sacred lotus and contributes novel, enantiospecific parts that could be deployed in synthetic biology platforms for the production of valuable phytopharmaceuticals. More research is required to isolate and characterize enzymes catalyzing 4′-*O*-methylation and *N*-methylation reactions, and further characterization of the identified CYPs with *R*-enantiomeric 1-benzylisoquinoline and aporphine substrates, when available, would be valuable. Additional discovery remains to determine the underlining mechanisms for enzymes responsible for the conversion of proaporphines to aporphines. BIA biosynthetic enzymes from sacred lotus also offer lucrative targets for structural and biochemical investigations with respect to substrate recognition, product formation, and underlying catalytic mechanisms.

## Methods

### Plant materials and reagents

Sacred lotus (*Nelumbo nucifera* Gaertn.) seeds were purchased with permission from Rarexoticseeds (Montreal, QC) and a voucher specimen of the plant used in this work has been deposited in the University of Calgary Herbarium (https://science.ucalgary.ca/about/faculty-office/collections-room/herbarium; unique identifier: IMenendez). Seeds were germinated in warm water until the emergence of the embryogenic leaves. Seedlings were planted in pots submerged in an artificial pond and grown as previously described^2^. To collect embryos, seeds were opened with a bench press. Plant samples harvested were flash-frozen in liquid nitrogen and stored at − 80 °C until used. The use of sacred lotus for the research conducted in this work complies with relevant institutional, national, and international guidelines and legislation.

(*R,S*)/(*S*)-Norcoclaurine, (*S*)-coclaurine, (*R,S*)-tetrahydropapaverine, (*R,S*)-laudanosine, (*S*)-6-*O*-methylnorlaudanosoline, dopamine, and 4-hydroxyphenylacetaldehyde were purchased from Toronto Research Chemicals (Toronto, ON); (*R*)-armepavine was purchased from MuseChem (Fairfield, NJ); (*R*)-reticuline was purchased from Santa Cruz Biotechnology (Santa Cruz, CA); and (*S*)-reticuline was a gift from Tasmanian Alkaloids Pty. Ltd. (Westbury, Australia). (*S*)-*N*-Methylcoclaurine and (*S*)-3′-hydroxy *N*-methylcoclaurine were produced from (*S*)-coclaurine or (*S*)-6-*O*-methylnorlaudanosoline, respectively, using recombinant *Glaucium flavum* CNMT and *S*-adenosyl-l-methionine as previously described^2^. Deuterium-labeled amino acids l-4-hydroxyphenyl-d4-alanine (l-tyrosine-d4) and l-phenyl-d5-alanine (l-phenylalanine-d5) were purchased from CDN Isotopes (Pointe-Claire, QC). (*R,S*)-4′-Deoxynorcoclaurine was chemically synthesized following a published protocol^44^ and the structure was confirmed based on the reported CID spectrum (Supplementary Table 8). All other reagents were acquired from Sigma-Aldrich (St-Louis, MO) or Bioshop Canada (Burlington, ON).

### Gene identification and phylogeny

A sacred lotus draft genome^22,23^ was searched to identify genes encoding enzymes with sequence similarity to the BIA biosynthetic enzymes norcoclaurine synthase (NCS), *O*-methyltransferases (OMTs), *N*-methyltransferase (NMTs), and cytochrome P450 monooxygenase (CYPs) from *Papaver somniferum* (i.e., PsNCS2, Ps4′OMT2, PsCNMT, PsCYP80B3, and PsCYP719A21) using the tBLASTn algorithm. Five NCS (NnNCS1, NnNCS3, NnNCS4, NnNCS5, and NnNCS7), three OMT (NnOMT6-8) in addition to five candidates previously characterized^2^, one CNMT (NnCNMT), and four CYP candidates (NnCYP80P1, NnCYP80Q1, NnCYP80Q2, and NnCYP719A22) were selected. Molecular weight and isoelectric point predictions were made using the Geneious software package (Biomatters, NJ). Amino acid sequence identity among sacred lotus gene candidates and functionally characterized orthologs was performed using Clustal Omega^70^ and sequence alignments were performed using the default parameters of the MUSCLE algorithm implemented in MEGA X^71^. Evolutionary history was inferred in MEGA X using the Maximum Likelihood method established on the Jones-Taylor-Thornton (JTT) matrix-based model. The bootstrap consensus tree inferred from 1000 replicates was taken to represent the evolutionary history of analyzed taxa and nodes were labeled according to the percentage of replicate trees in which the associated taxa clustered together in the bootstrap test. Protein sequences and GenBank™ accession numbers used in this work are provided in Supplementary Table 12.

### cDNA isolation and cloning

Sacred lotus leaves at folded and unfolded developmental stages, and embryos dissected from seeds were ground with a mortar and pestle to a fine powder under liquid nitrogen. Total RNA was extracted using the cetyltrimethylammonium bromide method, as previously reported^72^. RNA quality was confirmed by visualization in agarose gel electrophoresis, and by A_260/280_ and A_260/230_ absorbance measurements using a NanoDrop ND-1000 (Thermo Fisher Scientific). Genomic DNA was removed using an Applied Biological Materials AccuRT Genomics DNA Removal kit (Richmond, BC), and first-strand cDNA synthesis was performed using 1 µg of RNA, All-in-One RT MasterMix kit, following instructions of the manufacturer (Applied Biological Materials). Open reading frames were amplified from cDNA using New England Biolabs Q5 High Fidelity DNA polymerase and sequence-specific primers (Supplementary Table 13) using the following conditions: 98 °C (30 s); 35 cycles at 98 °C (10 s), 52–65 °C (optimized for each gene) for 30 s, and 72 °C (30 s); 72 °C for 2 min. Amplicons were cloned in the pMiniT 2.0 vector as recommended by the manufacturer (New England Biolabs), and used to transform the *Escherichia coli* TOP 10 strain for colony PCR screening. Plasmids from positive colonies were purified using a Thermo-Fisher Scientific GeneJet Plasmid Miniprep Kit (Waltham, MA) and sequenced.

### Quantitative real-time PCR

RNA extraction and cDNA synthesis were performed as described above. Candidate BIA biosynthetic gene expression was analyzed in folded and unfolded sacred lotus leaves, and in embryos^2^. Quantitative RT-PCR analysis was performed on four independent replicates for each sample using Applied Biosystems PowerUp SYBR Green Master Mix and a QuantStudio-3 Real-Time PCR System (Waltham, MA). Reactions (10 µl) contained sample cDNA (~ 10 ng), 5 µl of SYBR Green Master Mix (Applied Biosystems), and 500 nM of each primer (Supplementary Table 13). Thermal conditions were 50 °C (2 min), 95 °C (2 min), 40 cycles at 95 °C (1 s) and 60 °C (30 s). Melt-curve analysis was used to confirm amplification specificity using a ramp rate of 1.6 °C/s to 95 °C maintained for 15 s, 1.6 °C/s to 60 °C for 1 min, and 0.15 °C/s to 95 °C and sustained for 15 s. Primer efficiency was verified using Lin-RegPCR software and samples with values between 1.8 and 2 were selected for further analysis. Relative transcript abundance was determined by the 2^-ΔΔ*Ct*^ method^73^, using lotus *β*-actin as the endogenous reference gene. Transcript levels were normalized relative to the gene displaying the lowest expression level.

### NCS, OMT, and CNMT candidate gene expression and recombinant protein purification

Full-length coding regions corresponding to the sacred lotus *NnNCS*, *NnOMT,* and *NnCNMT* gene candidates were obtained by PCR using amplicons cloned into the pMiniT 2.0 vector as a template and corresponding specific primers (Supplementary Table 13). Amplicons were visualized in 1% (w/v) agarose gel and subsequently purified using a GeneJet Gel Extraction Kit (Thermo Fisher Scientific). Inserts were ligated into the Invitrogen pRSET-A plasmid (Waltham, MA) using one-step sequence and ligation-independent cloning^74^ to construct expression vectors. Open reading frames were cloned in-frame with sequences encoding an N-terminal His_6_ tag. The expression plasmids were used to transform the EMD Millipore Rosetta (DE3) pLysS *E. coli* strain (Burlington, MA), and single colonies were used to inoculate 50 ml of LB medium supplemented with ampicillin (100 µg/ml) and chloramphenicol (35 µg/ml). Culture growth, recombinant protein induction by the addition of isopropyl β-d-thiogalactoside (0.1 mM), and recombinant protein extraction were performed as previously described^2^. The cleared supernatant was loaded onto 1 ml of equilibrated Talon cobalt affinity resin (Clontech) and incubated at 4 °C for 30 min with gentle shaking. The resin was then washed with protein extraction buffer (100 mM Tris–HCl, pH 7.5, 300 mM NaCl, 10% (v/v) glycerol), subsequently rinsed with protein extraction buffer containing 20 mM imidazole and, finally, the purified protein was eluted using protein extraction buffer containing 200 mM imidazole. Subsequently, purified recombinant proteins were concentrated and desalted by repeated ultrafiltration on an Amicon Ultra 10 K (NCS) or 30 K (OMT and CNMT) column (EMD Millipore) in storage buffer (100 mM Tris–HCl, pH 7.5, 10% (v/v) glycerol, 1 mM β-mercaptoethanol). Protein concentration was determined using the Bradford reagent (Thermo-Fisher Scientific) and a BSA standard curve, and purity was assessed by SDS-PAGE electrophoresis with Coomassie staining. Purified His_6_-tagged proteins were confirmed by immunoblot analysis as described previously^2^, using an anti-His_6_ antibody and chemiluminescent detection with SuperSignal West Pico substrate (Thermo-Fisher Scientific), and visualized in a GE Healthcare Amersham Biosciences Imager 600 (Chicago, IL).

### Plant protein extraction

Sacred lotus leaves and embryos were ground to a fine powder under liquid nitrogen using a mortar and pestle, and proteins were isolated from ~ 1 g plant material in ice-cold extraction buffer (100 mM Tris–HCl, pH 7.5, 10% (v/v) glycerol, 1% (w/v) polyvinylpyrrolidone 40, 5 mM dithiothreitol, and 1 mM phenylmethylsulfonyl fluoride), as described previously^2^. The crude protein extract was desalted in a PD-10 column (GE Healthcare) and subsequently concentrated by repeated ultrafiltration in storage buffer containing 100 mM Tris–HCl, pH 7.5, 10% glycerol, and 1 mM β-mercaptoethanol and using an EMD Millipore Amicon Ultra 30 K column. Protein concentration was determined using the Bradford reagent and a BSA standard curve.

### In vitro enzyme assays

NCS assays were performed in 100 mM Tris–HCl, pH 7.5, using 500 µM dopamine (freshly prepared), 500 µM 4-HPAA or PAA, and 40 µg of purified recombinant protein in a reaction volume of 100 µl and incubated at 37 °C for 30 min. OMT assays were conducted in 100 mM Tris–HCl, pH 7.5, using 100 µM of various 1-benzylisoquinoline substrates including (*R,S*)/(*S*)norcoclaurine, (*S*)-coclaurine, (*S*)-*N*-methylcoclaurine, (*R*)-armepavine, and (*R*)/(*S*)-reticuline incubated with 200 µM SAM, and 20 µg of recombinant protein in a reaction volume of 50 µl incubated at 30 °C for 4 h or overnight. CNMT assays were performed using conditions similar to OMT assays, except that (*R,S*)-tetrahydropapaverine was also included as a potential substrate. Negative control assays were performed using purified recombinant protein denatured in boiling water for 10 min. Positive control assays were performed using purified, recombinant *Sanguinaria canadensis* NCS1, *Nelumbo nucifera* OMT1 and OMT5, *Papaver somniferum* 4′OMT2, or *Glaucium flavum* CNMT, prepared as previously described^2,11,75^. All assays were performed in triplicate. Reactions were quenched with acetonitrile (2:1, v/v), centrifuged at 17,000 × *g* for 40 min at 4 °C to precipitate proteins, and 5 µl of the supernatant was analyzed using positive-mode electrospray ionization [ESI( +)] high-performance liquid chromatography (HPLC)-tandem mass spectrometry (MS/MS). For NCS reactions, products were identified by comparison with norcoclaurine and 4′-deoxynorcoclaurine authentic standards. For the methyltransferase assays, reaction products showing a 14-Da increase compared with the corresponding substrate (equivalent to the incorporation of a methyl group) were identified by retention time and collision induced dissociation (CID) fragmentation comparison with available authentic standards or literature reports (Supplementary Table 8). Substrate conversion rates were calculated based on substrate loss. NnOMT7 pH and temperature optima assays and kinetic characterization were performed as previously described^2^ by incubating 1 µg of recombinant enzyme with (*R,S*)-norcoclaurine or (*S*)-*N*-methylcoclaurine (5–400 µM) at a fixed SAM concentration (400 µM) in 100 mM Tris–HCl, pH 7.5 buffer, at 30 °C for 5 min, and product formation was quantified by comparison with coclaurine or armepavine authentic standards, respectively. Saturation curves and kinetic parameters were determined based on the Michaelis–Menten equation using Prism 5 (GraphPad). Assays involving plant protein extracts were performed using similar conditions with 50 µg of protein; however, substrate concentration was increased to 1 mM for the NCS assays, and 200 µM and 400 µM for alkaloid substrates and SAM, respectively, for OMT and CNMT assays.

### Deuterium labeling experiment

Sacred lotus folded leaves were abraded using sandpaper to induce BIA biosynthesis^76,77^ and immediately submerged in a solution containing either water (negative control), 1 mM l-tyrosine-d4, or 1 mM l-phenylalanine-d5. Three independent replicates were performed for each treatment. After 3 days of incubation, the leaves were ground, and the alkaloids were extracted to investigate the incorporation of deuterated amino acids into sacred lotus BIAs. The alkaloid extraction method is described below. Identification was based on available authentic standards for deuterated amino acids, and non-deuterated l-tyrosine, l-phenylalanine, norcoclaurine, coclaurine, and *N*-methylcoclaurine, along with CID fragmentation data reported for pronuciferine and nuciferine (Supplementary Table 9). The relative amino acids and alkaloid content was determined as the average value of the specific peak area divided by the measured dry weight of the corresponding sample.

### *CYP* gene expression in engineered yeast

Full-length coding regions encoding sacred lotus CYPs were amplified by PCR using specific primers (Supplementary Table 13), inserted into the pMiniT 2.0 vector, visualized in 1% (w/v) agarose gel, and purified with the GeneJet Gel Extraction Kit (Thermo-Fisher Scientific). Inserts were ligated into the pEV2 plasmid (Agilent) using one-step sequence and ligation-independent cloning^74^ to obtain the expression vectors (Supplementary Note 1). Expression of the *CYP* open reading frames was driven by the strong THD3 promoter, and sequences encoding a C-terminal His_6_ tag (NnCYP80P1, NnCYP80Q1, and NnCYP80Q2) or HA tag (NnCYP719A22) were added. Expression plasmids were used to transiently transform *Saccharomyces cerevisiae* strain YNO-0, as previously described^78^ using the LiAc/PEG/single-stranded carrier DNA (ssDNA) method^79^. YNO-0 strain contains chromosomally integrated genes encoding *Papaver somniferum* BIA uptake permease 1 (PsBUP1) and CYP reductase 2 (PsCPR2), both C-terminal C_myc_-tagged (Supplementary Tables 11 and 14) and was constructed from the CEN.PK parent strain via CRISPR-Cas9 technology as previously described^80^.

### Yeast culture and substrate feeding

Four independent colonies corresponding to each yeast strain transformed with pEV2-NnCYP plasmids were used to inoculate individual wells of a 96-well plate, with each containing 500 µL of SD-His medium supplemented with 2% (w/v) glucose. As negative control, colonies transformed with the empty vector were used. Yeast cultures were grown overnight at 30 °C on a gyratory microplate shaker at 900 rpm. Subsequently, 25 µl of the culture was transferred to 500 µl of fresh medium containing either 5 µl (*S*)-*N*-methylcoclaurine (100 µM) or 20 µl lotus alkaloid extract and cultures were grown overnight at 30 °C on a gyratory microplate shaker at 900 rpm. Assays were quenched with 500 µl of acetonitrile, cells pelleted at 5000 × g for 30 min at 4 °C, and 5 µl of the supernatant were subjected to LC–MS/MS analysis.

### Plant alkaloid extraction and LC–MS analysis

Sacred lotus folded leaves were ground to a fine powder under liquid nitrogen using a mortar and pestle and alkaloids were extracted as previously described^2^. For deuterium labeling experiments, plant alkaloid samples (5 µl) were fractionated on a Zorbax C18 column, 2.1 × 50 mm, 1.8 µm (Agilent, CA) using a Dionex UltiMate 3000 HPLC system (Thermo-Fisher Scientific). The mobile phase consisted of solvent A (10 mM ammonium acetate, pH 5.5, 5% (v/v) acetonitrile) and solvent B (acetonitrile) at a flow rate of 500 µl/min, starting at 100% (v/v) solvent A, and ramping over 5 min from 0 to 20% (v/v) solvent B, over 3 min 20 to 50% (v/v) solvent B, over 3 min 50 to 100% (v/v) solvent B, remaining isocratic over 2 min at 100% (v/v) solvent B, ramping over 0.1 min from 100 to 0% (v/v) solvent B, and remaining isocratic over 1.9 min at 100% (v/v) solvent A, for a total run time of 15 min (data collected for the first 10 min). Heated ESI source and interface conditions were operated in positive ion mode as follows: vaporizer temperature 400 °C, source voltage 3 kV, sheath gas 60 au, auxiliary gas 20 au, capillary temperature 380 °C, capillary voltage 6 V, tube lens 45 V. The LTQ-Orbitrap-XL (ThermoFisher Scientific) mass spectrometer was operated using LTQ Tune Plus (version 2.5.5 SP1) and Xcalibur software (version 2.1.0.1140), with additional analyses using the QualBrowser feature of Xcalibur. Error was maintained below 2 ppm. Exact masses, retention times, and CID spectra of available authentic standards or previously published data were used for alkaloid identification (Supplementary Table 9).

Enzyme assays and yeast feeding experiments were analyzed by LC–MS/MS using an Agilent 1200 LC coupled to a 6410 triple quadrupole MS (Santa Clara, CA). Samples (5 µl) were injected onto a Poroshell 120 SB-C18 HPLC column, 2.1 × 50 mm, 2.7 µm particle size (Agilent). Analytes were eluted using a mobile phase gradient of solvent A (water/acetonitrile, 95/5 (v/v), 0.1% formic acid) and solvent B (acetonitrile, 0.1% formic acid), at a flow rate of 600 µl/min. The gradient started at 100% (v/v) solvent A, ramped linearly to 60% (v/v) solvent B by 8 min, further increased linearly to 99% (v/v) solvent B over 2 min, remained isocratic at 99% (v/v) solvent B from 10 to 11 min, and returned to 100% (v/v) solvent A at 11.1 min for a 3 min equilibration period. Analytes were applied to the mass analyzer using an electrospray ionization probe operating in positive mode and using previously described conditions^2^.

## Supplementary Information

Supplementary Information.

## Data Availability

The datasets generated and/or analysed during the current study are available in the NCBI repository, with accession numbers as follows: NnCYP719A22 (OP795716), NnCYP80Q1 (OP795717), NnCYP80Q2 (OP795718), NnCYP80P1 (OP795719), NnCNMT (OP795720), NnOMT1 (OP795721), NnOMT5 (OP795722), NnOMT6 (OP795723), NnOMT7 (OP795724), and NnOMT8 (OP795725).
