# Supplementary material for: Elucidation of the (R)-enantiospecific benzylisoquinoline alkaloid biosynthetic pathways in sacred lotus (Nelumbo nucifera)
Source: Sci Rep. 2023 Feb 20;13:2955. doi: 10.1038/s41598-023-29415-0 (PMC9940101; doi:10.1038/s41598-023-29415-0)
Supplement: Supplementary file 1 — Supplementary Information. [file 41598_2023_29415_MOESM1_ESM.pdf]

Supplementary Information

Elucidation of the (*R*)-enantiospecific benzylisoquinoline alkaloid biosynthesis in sacred lotus (*Nelumbo nucifera*)

Ivette M. Menéndez-Perdomo and Peter J. Facchini

Supplementary Figures

Supplementary Fig. 1: Summary of BIA biosynthetic pathways leading to the major alkaloids in opium poppy.
Supplementary Fig. 2: Phylogenetic analysis of sacred lotus BIA biosynthetic enzyme candidates and functionally characterized homologs from BIA-accumulating species in the Ranunculales.
Supplementary Fig. 3: Partial multiple sequence alignment of functionally characterized norcoclaurine synthases and sacred lotus homologs.
Supplementary Fig. 4: Partial multiple sequence alignment of functionally characterized *O*-methyltransferases and sacred lotus homologs.
Supplementary Fig. 5: Partial multiple sequence alignment of functionally characterized coclaurine *N*-methyltransferases and sacred lotus homologs.
Supplementary Fig. 6: Partial multiple sequence alignment of functionally characterized cytochromes P450 (CYP80 family) and sacred lotus homologs.
Supplementary Fig. 7: Partial multiple sequence alignment of functionally characterized cytochromes P450 (CYP719A subfamily) and sacred lotus homologs.
Supplementary Fig. 8: Immunoblot detection of recombinant proteins from sacred lotus tested as candidates for norcoclaurine synthase (NCS), *O*-methyltransferase (OMT), and *N*-methyltransferase.
Supplementary Fig. 9: *In vitro* screening of recombinant NnNCS candidates and plant protein extracts for norcoclaurine synthase activity.
Supplementary Fig. 10: Deuterated L-phenylalanine is not incorporated into alkaloids in sacred lotus folded leaves.
Supplementary Fig. 11: *In vitro* activity of sacred lotus NnOMT7 and NnCNMT.
Supplementary Fig. 12: Biochemical characterization of NnOMT7.
Supplementary Fig. 13: Activity of *O*-methyltransferases (NnOMTs) and *N*-methyltransferase (NnCNMT) candidates on potential substrates.
Supplementary Fig. 14: Activity of sacred lotus folded leaf protein extracts on exogenous norcoclaurine.
Supplementary Fig. 15: 6-*O*-Methyltransferase activity on sacred lotus folded leaf alkaloid extract.
Supplementary Fig. 16: (*S*)-*N*-Methylcoclaurine is not a substrate for sacred lotus cytochromes P450.
Supplementary Fig. 17: CID fragmentation of reaction products detected in yeast strains fed with sacred lotus alkaloid extract.
Supplementary Fig. 18: Proposed biosynthesis of proaporphines and aporphines in sacred lotus.
Supplementary Fig. 19: Proposed catalytic mechanism for proaporphine formation from a 1benzylisoquinoline substrate, and subsequent conversion to a corresponding aporphine.
Supplementary Fig. 20: Schematic representation of bisbenzylisoquinoline alkaloids biosynthesis in sacred lotus.
Supplementary Fig. 21: Proposed catalytic mechanism for the head-to-tail formation of bisbenzylisoquinolines in sacred lotus.
Supplementary Fig. 22: Proposed catalytic mechanism for methylenedioxy bridge formation in the isoquinoline moiety of aporphine alkaloids in sacred lotus.

Supplementary Information

Supplementary Tables

Supplementary Table 1: Coding sequences of BIA biosynthetic gene candidates from sacred lotus.
Supplementary Table 2: Predicted molecular masses and isoelectric points (pI) of biosynthetic enzyme candidates from sacred lotus.
Supplementary Table 3: Amino acid percent identity matrix for sacred lotus norcoclaurine synthase (NnNCS) candidates and several functionally characterized, single-domain NCS enzymes involved in BIA biosynthesis in the Ranunculales.
Supplementary Table 4: Amino acid percent identity matrix for sacred lotus *O*-methyltransferase (NnOMT) candidates and several functionally characterized OMTs involved in BIA biosynthesis in the Ranunculales.
Supplementary Table 5: Amino acid percent identity matrix for the sacred lotus coclaurine *N*-methyltransferase (NnCNMT) candidate and several functionally characterized NMTs involved in BIA biosynthesis in the Ranunculales.
Supplementary Table 6: Amino acid percent identity matrix for sacred lotus cytochrome P450 (NnCYP80) candidates and several functionally characterized CYP80s from BIA-accumulating species in the Ranunculales.
Supplementary Table 7: Amino acid percent identity matrix for the sacred lotus cytochrome P450 (NnCYP719A) candidate and several functionally characterized CYP719As from BIA-accumulating species in the Ranunculales.
Supplementary Table 8: Chromatographic and mass spectral data for BIAs detected in enzyme assays, in engineered yeast and in sacred lotus plants.
Supplementary Table 9: Chromatographic and mass spectral data for compounds detected in sacred lotus plants fed deuterium-labeled amino acids.
Supplementary Table 10: Kinetic parameters for recombinant NnOMT7.
Supplementary Table 11: Engineered yeast (*Saccharomyces cerevisiae*) strains used to assay the function of NnCYP candidates.
Supplementary Table 12: Amino acid sequences of sacred lotus candidate enzymes and functionally characterized homologs involved in BIA biosynthesis in the Ranunculales.
Supplementary Table 13: Sequence-specific primers used in this work.
Supplementary Table 14: Nucleotide sequences of opium poppy genes integrated into the YNO-0 yeast strain.

Supplementary Notes

Supplementary Note 1: Complete nucleotide sequence of pEV2.

Supplementary References

Supplementary Information

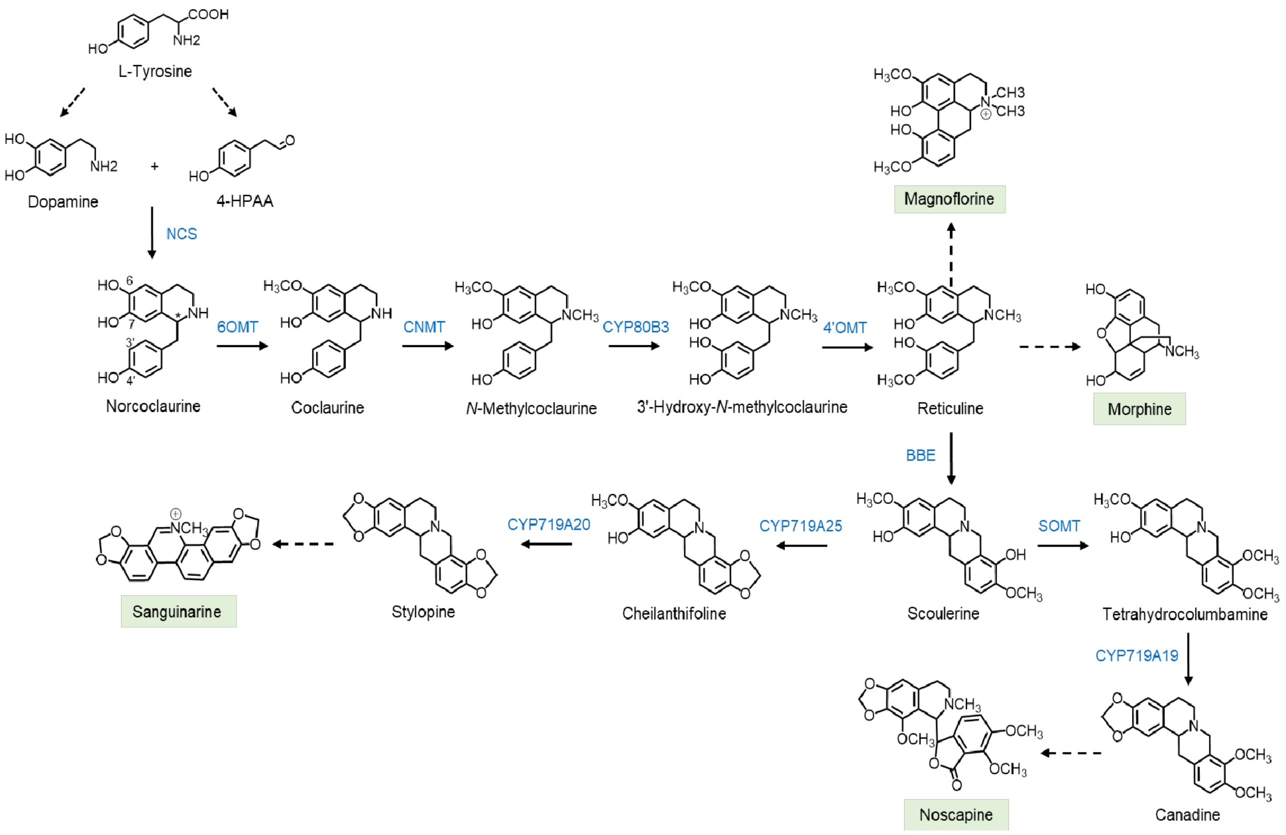

Supplementary Fig. 1: Summary of BIA biosynthetic pathways leading to major alkaloids in opium poppy. The enzymes shown are potential homologs of those involved in BIA biosynthesis in sacred lotus. Arrows with dashed lines indicate multiple reactions. Abbreviations: 4'OMT, 3'-hydroxy-*N*-methylcoclaurine 4'-*O*-methyltransferase; 6OMT, norcoclaurine 6-*O*-methyltransferase; BBE, berberine bridge enzyme; CNMT, coclaurine *N*-methyltransferase; CYP80B3, *N*-methylcoclaurine 3'-hydroxylase; CYP719A19, canadine synthase; CYP719A20, cheilanthifoline synthase; CYP719A25, stylopine synthase; NCS, norcoclaurine synthase; SOMT, scoulerine 9-*O*-methyltransferase. Asterisk denotes the chiral center in norcoclaurine, and numbers refer to carbon atoms.

Supplementary Information

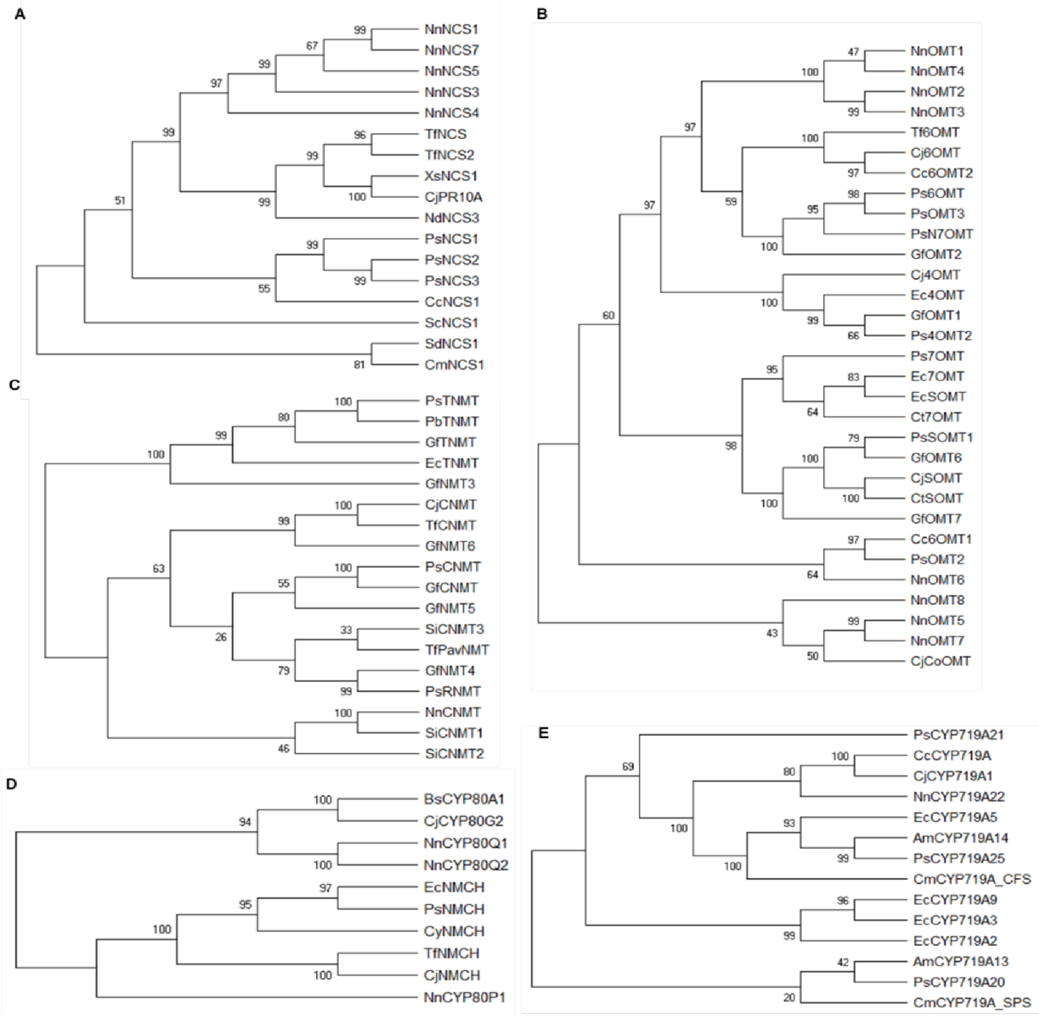

Supplementary Fig. 2: Phylogenetic analysis of sacred lotus BIA biosynthetic enzyme candidates and functionally characterized homologs from BIA-accumulating species in the Ranunculales. (**A**) Norcoclaurine synthases (NCS), (**B**) *O*-methyltransferases (OMT), (**C**) *N*-methyltransferases (NMT), (**D**) cytochromes P450 (CYP80) family, and (**E**) cytochromes P450 (CYP719A) subfamily. Evolutionary history was inferred using the Maximum Likelihood and the JTT matrix-based model. The bootstrap consensus tree inferred from 1000 replicates represents the phylogenetic relationship among proteins. The percentage of replicate trees in which the associated taxa clustered together in the bootstrap test is shown next to the branches. Evolutionary analyses were conducted in MEGAX. Am, *Argemone mexicana*; Bs, *Berberis stolonifera*; Cc, *Coptis chinensis*; Cj, *C. japonica*; Ct, *C. teeta*; Cm, *Chelidonium majus*; Cy, *Corydalis yanhusuo*; Ec, *Eschscholzia californica*; Gf, *Glaucium flavum*; Nd, *Nandina domestica*; Nn, *Nelumbo nucifera*; Pb, *Papaver bracteatum*; Ps, *P. somniferum*; Sc, *Sanguinaria canadensis*; Sd, *Stylophorum diphyllum*; Si, *Stephania intermedia*; Tf, *Thalictrum flavum*; Xs, *Xanthorhiza simplicissima.* 4OMT, 3'-hydroxy-*N*-methylcoclaurine 4'-*O*-methyltransferase; 6OMT, norcoclaurine 6-*O*-methyltransferase; 7OMT, reticuline 7-*O*-methyltransferase; CNMT: coclaurine *N*-methyltransferase; CoOMT, columbamine *O*-methyltransferase; CYP719A, methylenedioxy bridge-forming enzymes; CYP80A, bisbenzylisoquinoline synthases; CYP80B, 3'-hydroxylases; CYP80G, aporphine synthases; N7OMT, norreticuline 7-*O*-methyltransferase; OMT, *O*-methyltransferase; PavNMT, pavine *N*-methyltransferase; RNMT, reticuline *N*-methyltransferase; SOMT, scoulerine 9-*O*-methyltransferase; TNMT, tetrahydropapaverine *N*-methyltransferase.

Supplementary Information

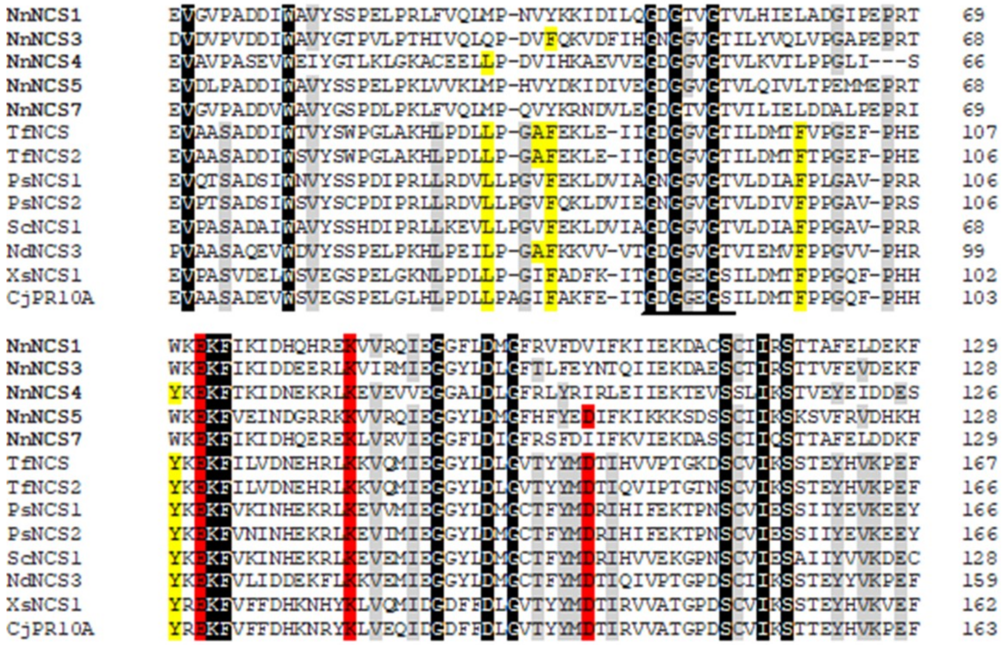

Supplementary Fig. 3: Partial multiple sequence alignment of functionally characterized norcoclaurine synthases and sacred lotus homologs. Alignment includes sacred lotus NCS candidates (NnNCS1, NnNCS3, NnNCS4, NnNCS5, and NnNCS7) and functionally characterized single-domain NCS enzymes from *Thalictrum flavum* (TfNCS and TfNCS2), *Papaver somniferum* (PsNCS1 and PsNCS2), *Sanguinaria canadensis* (ScNCS1), *Nandina domestica* (NdNCS3), *Xanthorhiza simplicissima* (XsNCS1), and *Coptis japonica* (CjPR10A). Fully conserved residues are shaded in black and those conserved among sacred lotus candidates and/or all functionally characterized NCS enzymes from the Ranunculales are shown in grey. The conserved glycine-rich loop characteristic of the PR10/Bet v1 protein family is underlined. TfNCS catalytic determinants (E110, K122, and D141) are shown in red, and key residues implicated in substrate binding (L76, G78, A79, F80, F99, and Y108) are indicated in yellow.

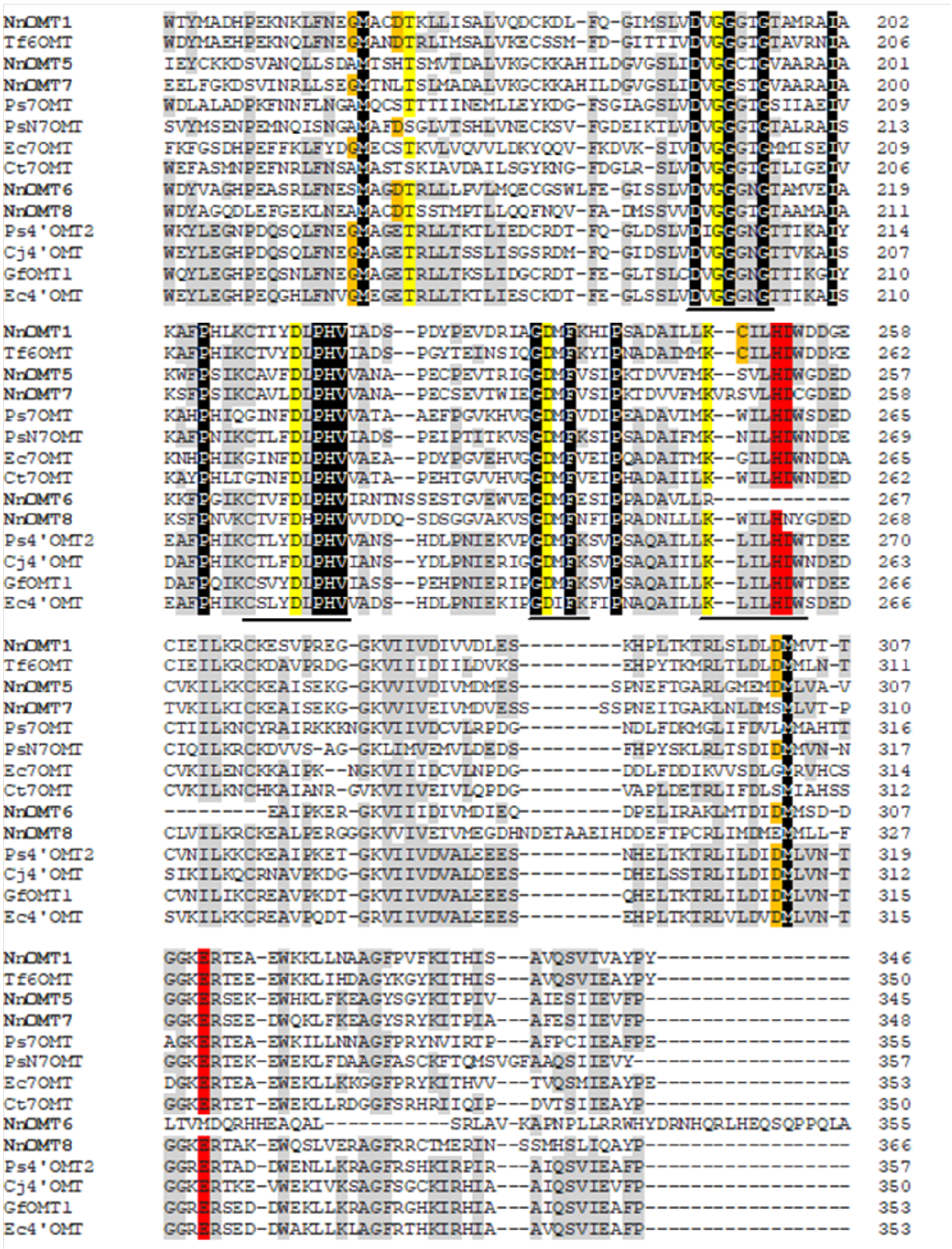

Supplementary Fig. 4: Partial multiple sequence alignment of functionally characterized *O*-methyltransferases and sacred lotus homologs. Alignment includes new (NnOMT6-8) and previously reported (NnOMT1-5) sacred lotus OMT candidates, and functionally characterized OMTs involved in 1benzylisoquinoline *O*-methylation from *Thalictrum flavum* (Tf6OMT), *Papaver somniferum* (Ps7OMT, PsN7OMT, and Ps4'OMT2), *Eschscholzia californica* (Ec7OMT and Ec4'OMT), *Coptis teeta* (Ct7OMT), *Coptis japonica* (Cj4'OMT), *Glaucium flavum* (GfOMT1), and *Nelumbo nucifera* (NnOMT1 and NnOMT5). Fully conserved residues are shaded in black and those conserved among sacred lotus candidates and/or all functionally characterized OMTs from the Ranunculales are shown in grey. Motifs conserved on all OMTs are underlined. Tf6OMT catalytic determinants (H256, D257, and E315) are shown in red, and key residues implicated in alkaloid binding (G165, D169, C253, and D306) and *S*-adenosyl-methionine binding (T170, G195, D218, D238, and K252) are indicated in orange and yellow, respectively.

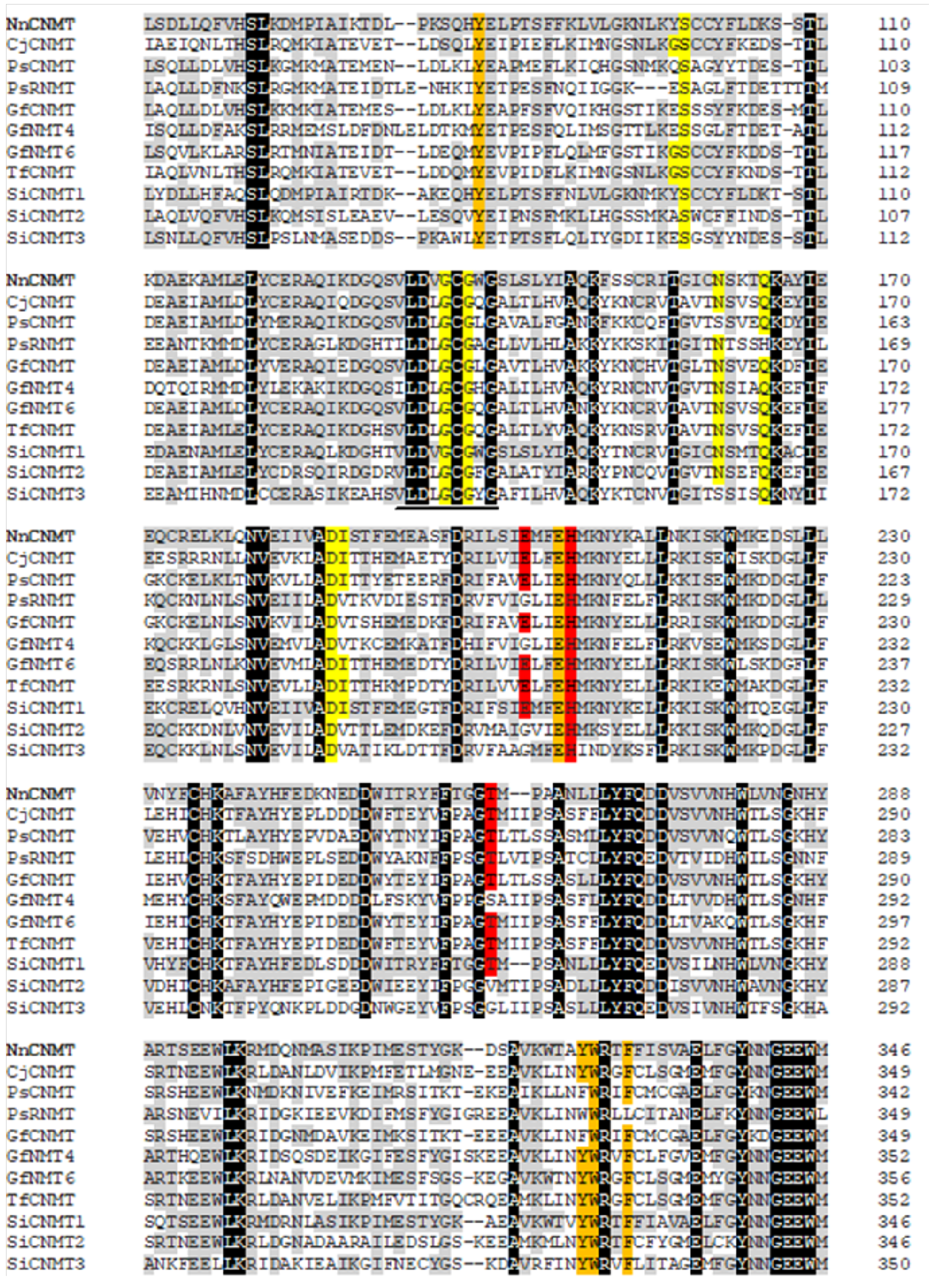

Supplementary Fig. 5: Partial multiple sequence alignment of functionally characterized coclaurine *N*-methyltransferases and sacred lotus homologs. Alignment includes the sacred lotus CNMT candidate (NnCNMT), and functionally characterized NMTs involved in 1-benzylisoquinoline *N*methylation from *Coptis japonica* (CjCNMT), *Papaver somniferum* (PsCNMT and PsRNMT), *Glaucium flavum* (GfCNMT, GfNMT4, and GfNMT6), *Stephania intermedia* (SiCNMT1, SiCNMT2, and SiCNMT3), and *Thalictrum flavum* (TfCNMT). Fully conserved residues are shaded in black and those conserved among sacred lotus candidates and/or all functionally characterized NMTs from the Ranunculales are shown in grey. Motif I conserved in all related NMTs is underlined. CjCNMT catalytic determinants (E204, H208, and T261) are shown in red, and key residues implicated in alkaloid binding (Y81, E207, Y328, W329, and F332 and *S*-adenosylmethionine binding (G98, S99, G137, G139, N161, Q165, D187, and I188) are indicated in orange and yellow, respectively.

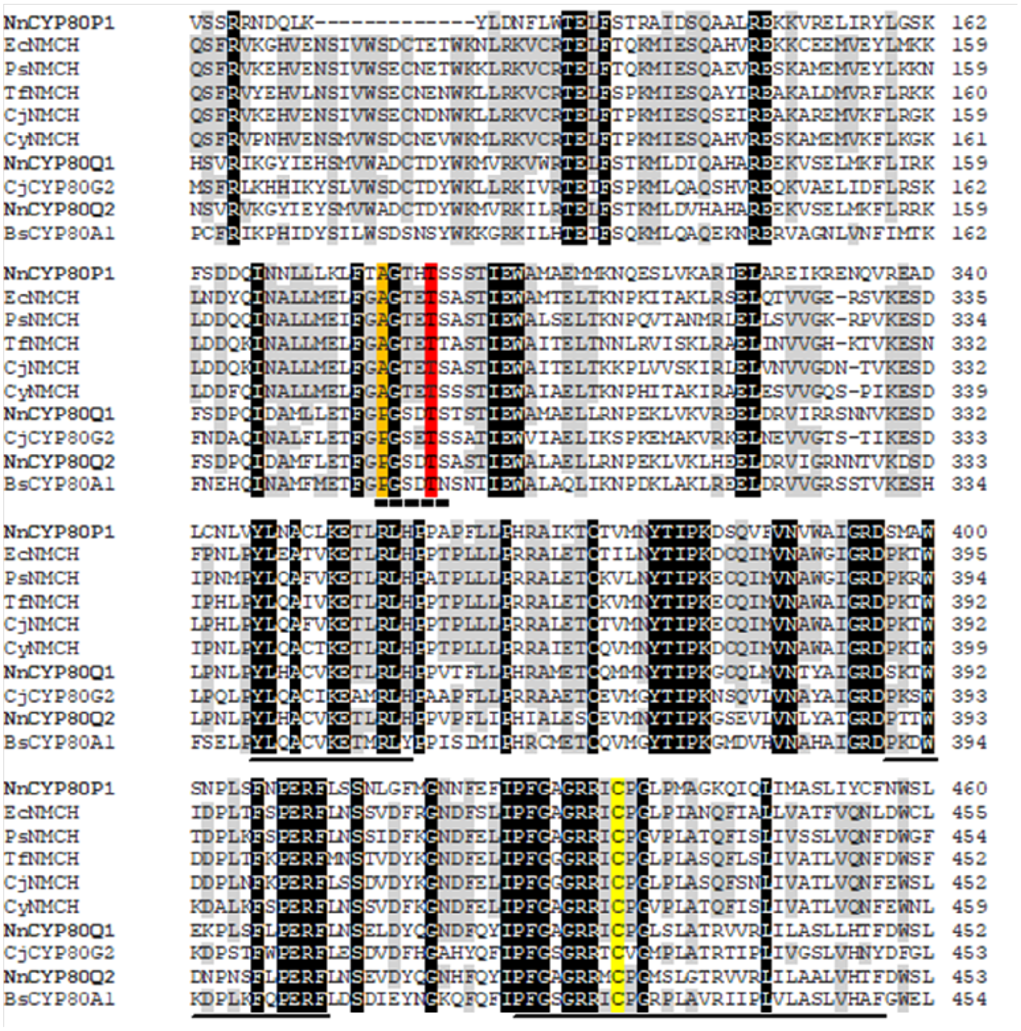

Supplementary Fig. 6: Partial multiple sequence alignment of functionally characterized cytochromes P450 (CYP80 family) and sacred lotus homologs. Alignment includes sacred lotus CYP80 candidates (NnCYP80P1, NnCYP80Q1, and NnCYP80Q2), and functionally characterized 1benzylisoquinoline 3'-hydroxylases from *Coptis japonica* (CjNMCH), *Corydalis yanhusuo* (CyNMCH), *Eschscholzia californica* (EcNMCH), *Papaver somniferum* (PsNMCH), and *Thalictrum flavum* (TfNMCH), an aporphine synthase from *C. japonica* (CjCYP80Q2), and a bisbenzylisoquinoline synthase from *Berberis stolonifera* (BsCYP80A1). Fully conserved residues are shaded in black and those conserved among sacred lotus candidates and/or all functionally characterized CYP80 family enzymes from the Ranunculales are shown in grey. Conserved motifs in eukaryotic cytochromes P450, including helix K, aromatic, and heme-binding regions are indicated with a solid line, and the consensus sequence (A/G)GX(D/E)T(T/S) with a dashed line. The CjCYP80G2 catalytic determinant (T294) is shown in red, and key residues (P290 and C428) implicated in substrate binding and heme-group interactions are indicated in orange and yellow, respectively.

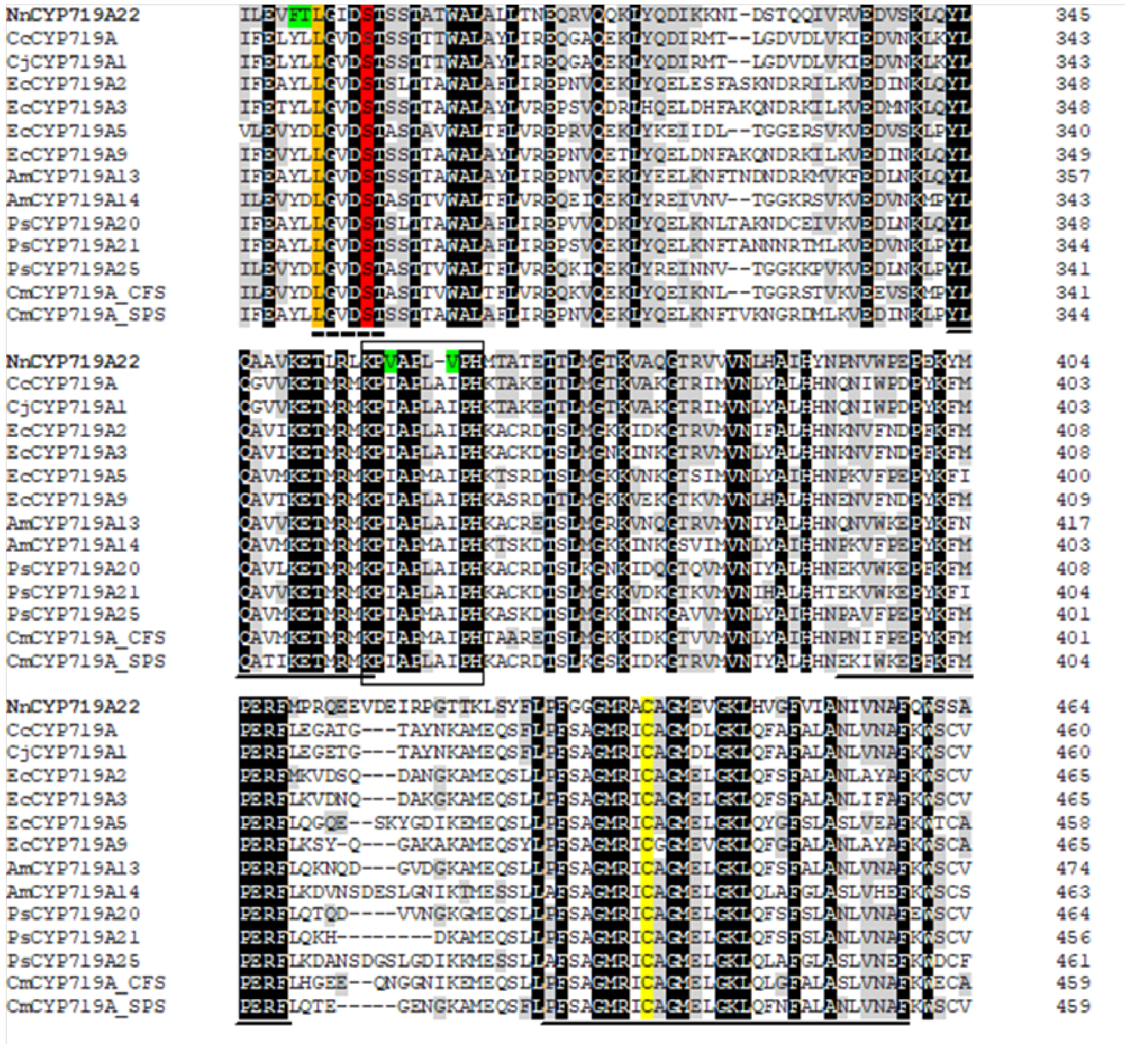

Supplementary Fig. 7: Partial multiple sequence alignment of functionally characterized cytochromes P450 (CYP719A subfamily) and sacred lotus homologs. Alignment includes the sacred lotus CYP719A candidate (NnCYP719A22), and functionally characterized methylenedioxy bridge-forming enzymes from *Coptis chinensis* (CcCYP719A), *C. japonica* (CjCYP719A1), *Eschscholzia californica* (EcCYP719A2, EcCYP719A3, EcCYP719A5, and EcCYP719A9), *Argemone mexicana* (AmCYP719A13 and AmCYP719A14), *Papaver somniferum* (PsCYP719A20, PsCYP719A21, and PsCYP719A25), and *Chelidonium majus* (CmCYP719A_CFS and CmCYP719A_SPS). Fully conserved residues are shaded in black and those conserved among sacred lotus candidates and/or all functionally characterized CYP719A subfamily enzymes from the Ranunculales are shown in grey. Conserved motifs in eukaryotic cytochromes P450, including helix K, aromatic, and heme-binding regions are indicated with a solid line, and the consensus sequences (A/G)GX(D/E)T(T/S) and KPIAPXXXPH with a dashed line and a box, respectively. Functional members of the CYP719A subfamily exhibit a replacement of the conserved catalytic threonine with serine (S296) shown in red. A conserved leucine (L292) is indicated in orange, the phenylalanine, threonine, and two valine replacing conserved residues tyrosine, leucine/aspartic acid, and isoleucine residues (F291, T292, V358, and V362), respectively are represented in green, and cysteine involved in heme-group interaction (C434) is represented in yellow.

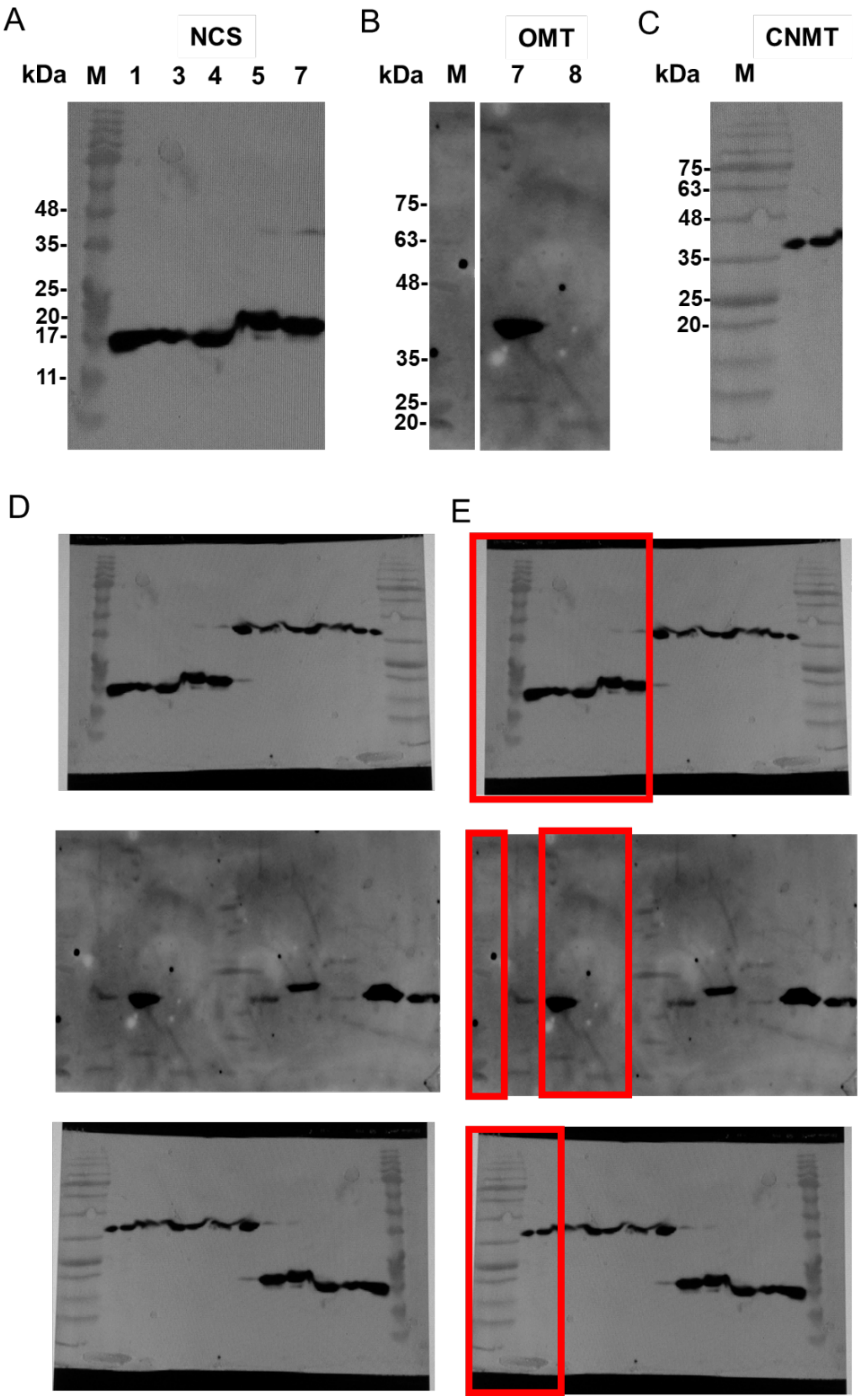

Supplementary Fig. 8: Immunoblot detection of recombinant proteins from sacred lotus tested as candidates for (A) norcoclaurine synthase (NCS), (B) *O*-methyltransferase (OMT), and (C) *N*-methyltransferase. Soluble, recombinant His_6-tagged proteins were produced in *Escherichia coli* and isolated by cobalt-affinity purification. Molecular weights of marker proteins (L) are indicated. Uncropped gel images (**D**) are shown with red rectangles highlighting the parts used in panels **A**, **B**, and **C** (**E**).

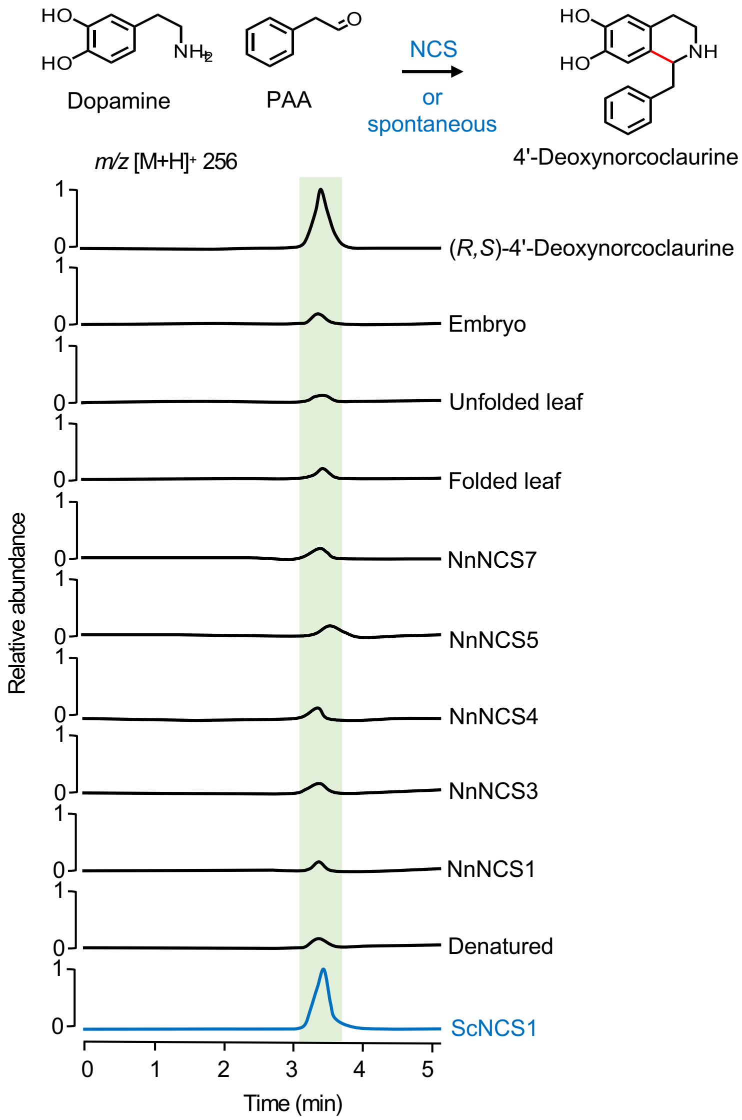

Supplementary Fig. 9: *In vitro* screening of recombinant NnNCS candidates and plant protein extracts for norcoclaurine synthase activity. Chromatograms show the relative formation of 4'-deoxynorcoclaurine (*m/z* [M+H]$^+$ 256) from dopamine and phenylacetaldehyde (PAA). Authentic (*R,S*)-4'-deoxynorcoclaurine was used for retention time and mass spectrometric data comparisons. *Sanguinaria canadensis* NCS1 (in blue) was used as a positive control for the enzyme-catalyzed reaction. Spontaneous, non-enzymatic condensation was detected using denatured ScNCS1 protein as a negative control.

Supplementary Information

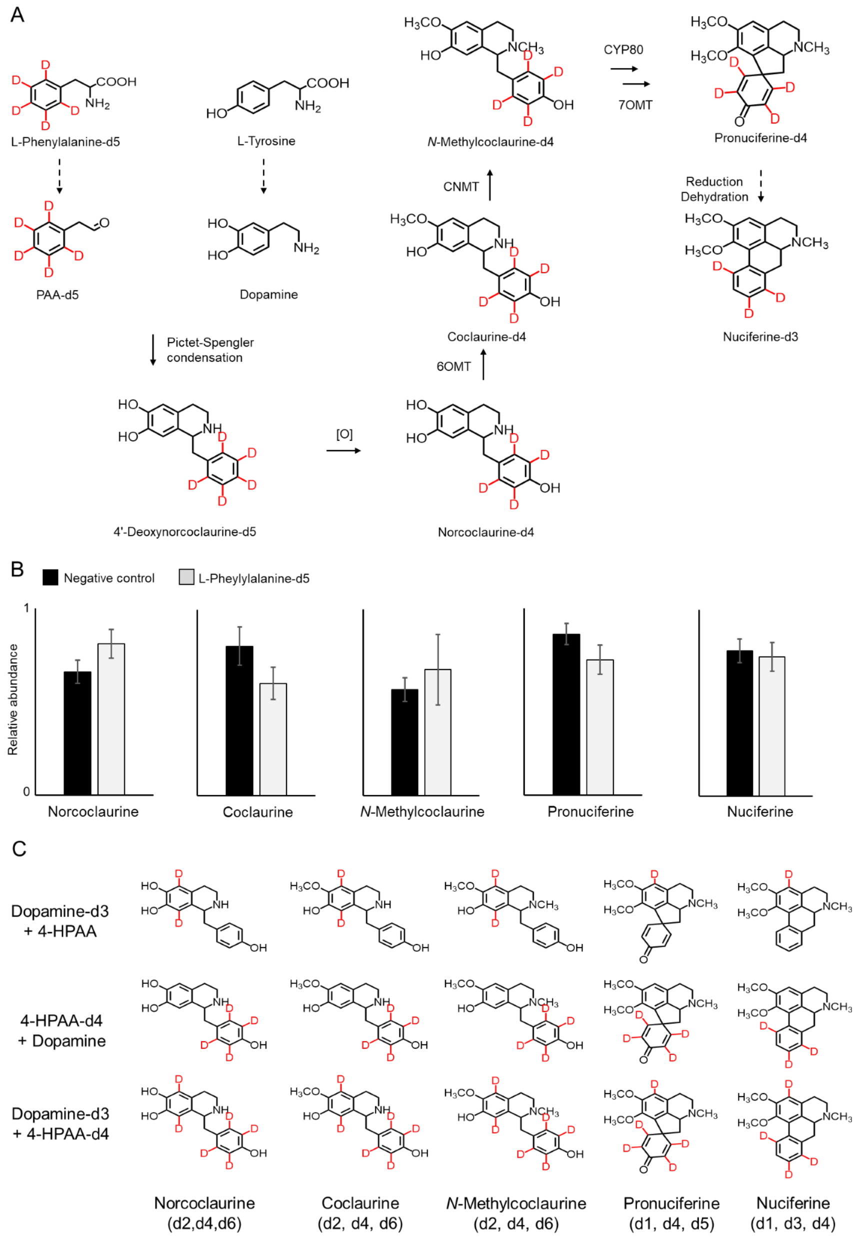

Supplementary Fig. 10: Deuterated L-phenylalanine is not incorporated into alkaloids in sacred lotus folded leaves. (**A**) If phenylalanine is a precursor to BIAs in sacred lotus, dopamine (derived from tyrosine) and phenylacetaldehyde (PAA)-d5 (derived from phenylalanine-d5) should condense and form 4'-deoxynorcoclaurine-d5. Subsequent 4'-hydroxylation would generate norcoclaurine-d4 and downstream products, such as coclaurine-d4, *N*-methylcoclaurine-d4, pronuciferine-d4, and nuciferine-d3. The red deuterium (D) atoms show the labeled positions in each structure. Dashed arrows indicate more than one reaction. (**B**) Graphs represent the relative abundance of naturally occurring alkaloids in the negative control and in L-phenylalanine-d5 fed plants. (**C**) All possible intermediates resulting from diverse combinations of labeled and non-labeled dopamine and 4-HPAA detected in the L-tyrosyne-d4 treatment. Values represent the mean ± SD of three independent measurements. Abbreviations: PAA, phenylacetaldehyde; CNMT, coclaurine *N*-methyltransferase; CYP80, cytochrome P450 monooxygenase; NCS, norcoclaurine synthase; OMT, *O*-methyltransferase.

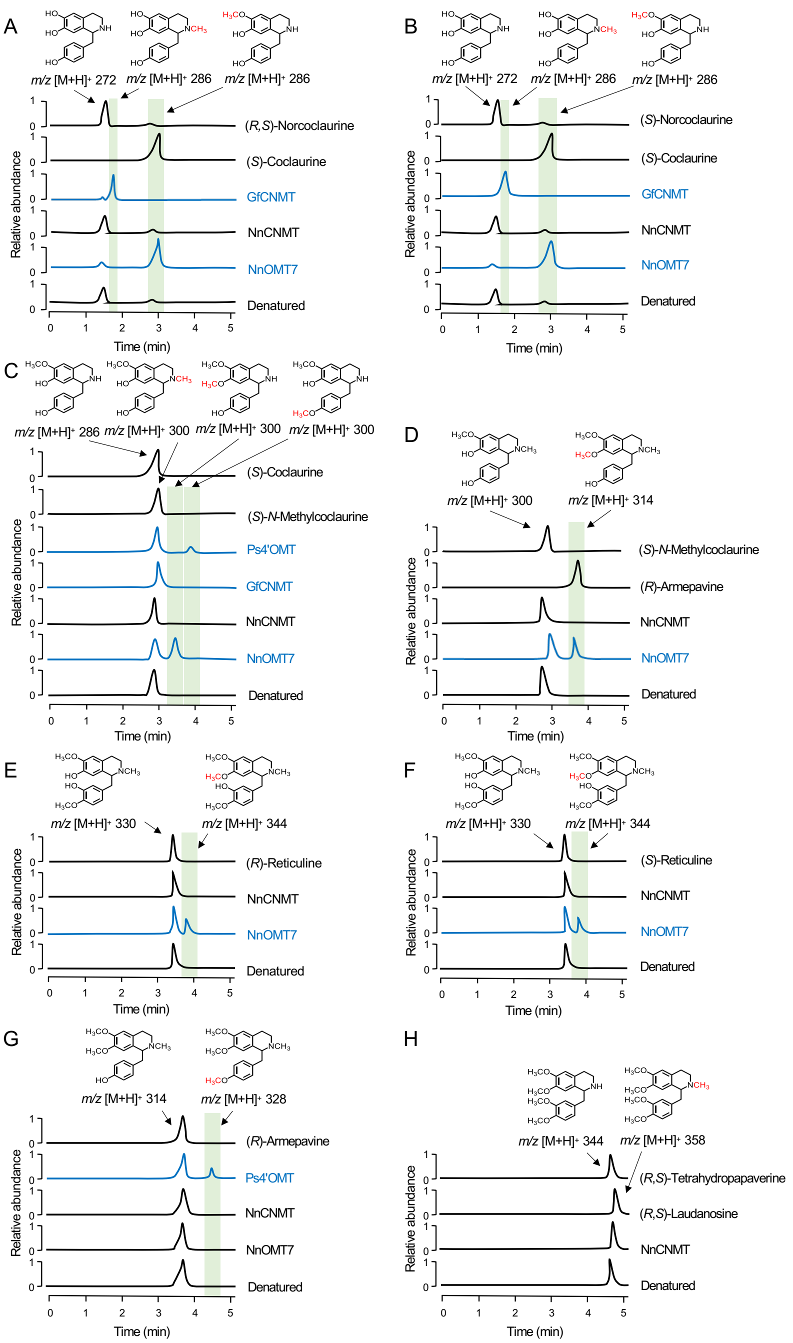

Supplementary Fig. 11: *In vitro* activity of sacred lotus NnOMT7 and NnCNMT. Chromatograms represent catalytic activity on the following substrates: (**A**) (*R,S*)-norcoclaurine, (**B**) (*S*)-norcoclaurine, (**C**) (*S*)-coclaurine, (**D**) (*S*)-*N*-methylcoclaurine, (**E**) (*R*)-reticuline, (**F**) (*S*)-reticuline, (**G**) (*R*)-armepavine, and (**H**) (*R,S*)-tetrahydropapaverine. Authentic standards were used for retention time and mass spectrometric data comparisons. Active enzyme chromatograms, including positive *Glaucium flavum* CNMT and *Papaver somniferum* 4'OMT controls, are indicated in blue and the signal corresponding to the reaction product(s) is highlighted in green. Structures corresponding to reaction substrate and products are shown.

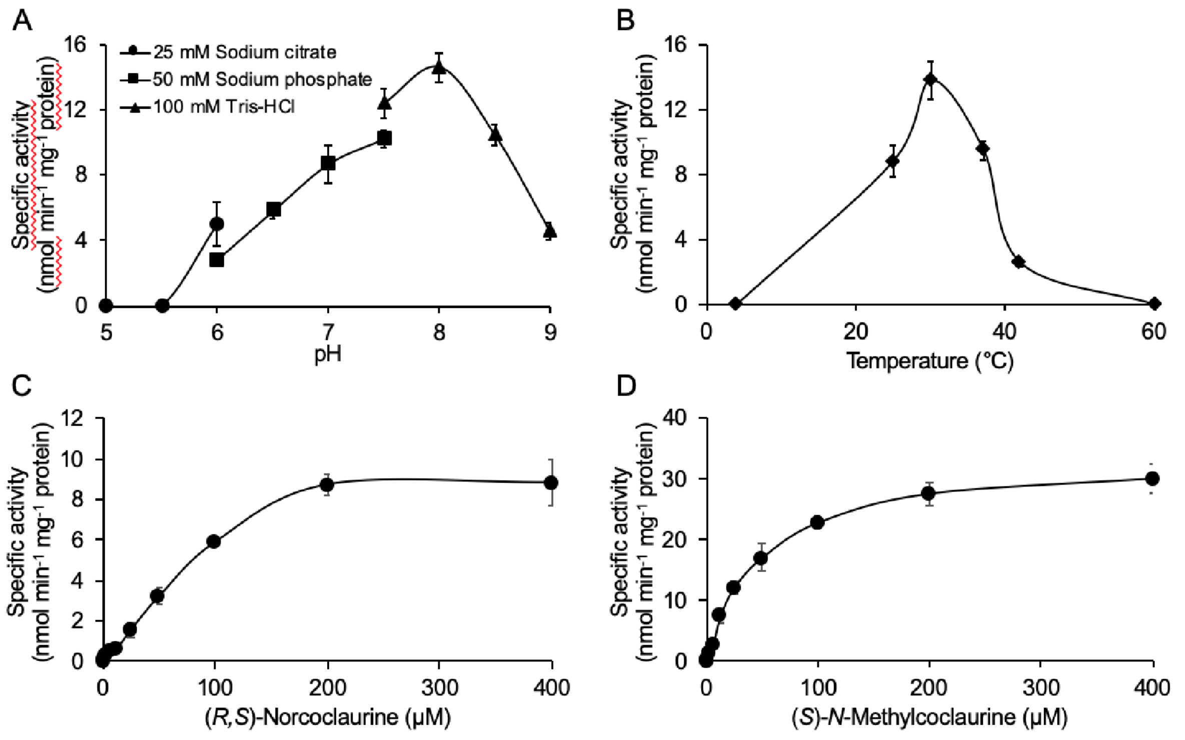

Supplementary Fig. 12: Biochemical characterization of NnOMT7. Effect of (**A**) pH and (**B**) temperature on the 7-*O*-methylation activity of recombinant NnOMT7 using (*S*)-*N*-methylcoclaurine and *S*-adenosylmethionine (SAM) as substrates. (**C**) Steady-state enzyme kinetics for NnOMT7 at fixed SAM concentration and using various concentrations of (*R,S*)-norcoclaurine, to detect 6-*O*-methylation activity, and (*S*)-*N*-methylcoclaurine, to measure 7-*O*-methylation activity. Values represent the mean ± standard deviation of three independent measurements.

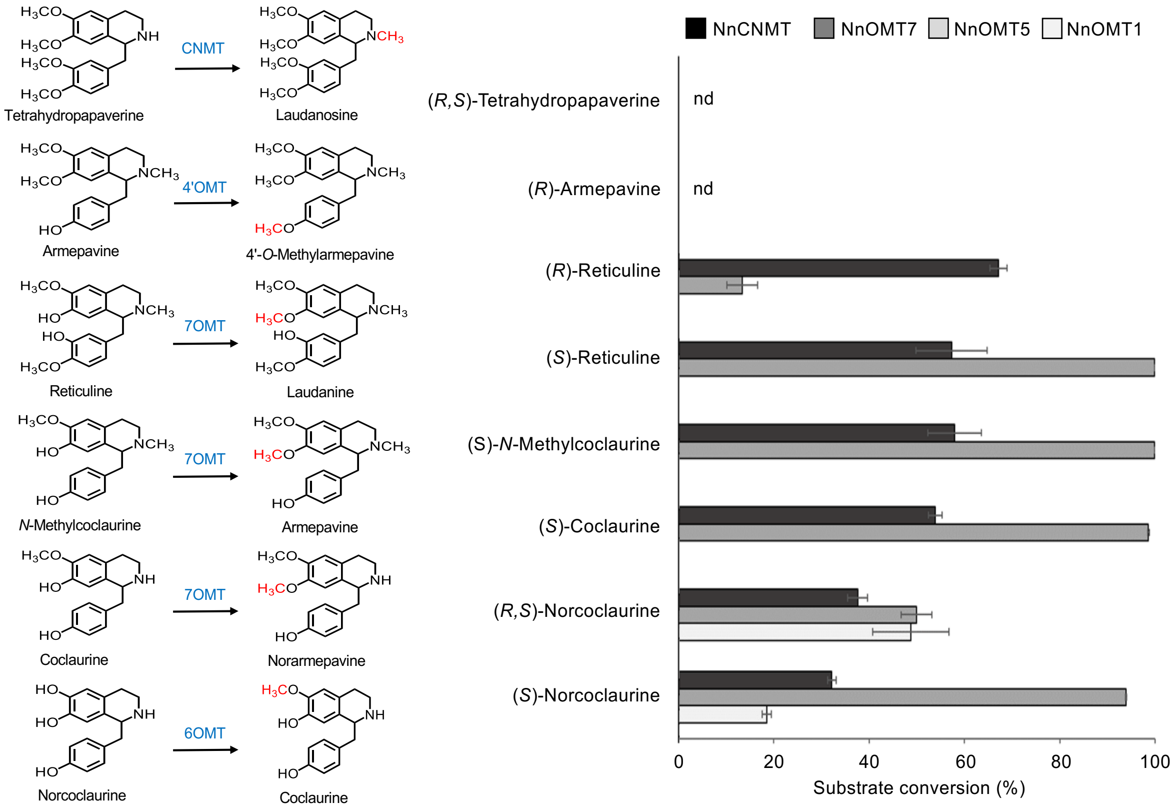

Supplementary Fig. 13: Activity of *O*-methyltransferases (NnOMTs) and *N*-methyltransferase (NnCNMT) candidates on potential substrates. Substrates and predicted reaction products, and relative substrate conversion rates of recombinant NnCNMT, NnOMT1, NnOMT5, and NnOMT7 in assays incubated overnight. Reaction products corresponding to the only possible methylations of (*R*)-armepavine and (*R,S*)-tetrahydropapaverine were not detected. Bars represent the mean ± standard deviation of three independent measurements. n.d., not detected.

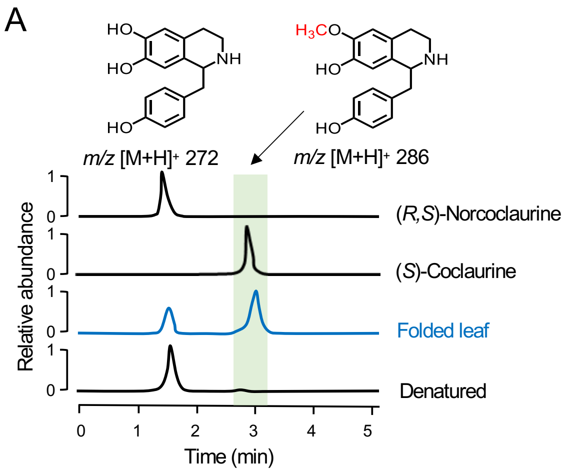

Supplementary Fig. 14: Activity of sacred lotus folded leaf protein extracts on exogenous norcoclaurine. Enantiomers tested were (**A**) (*R,S*)-norcoclaurine and (**B**) (*S*)-norcoclaurine. Authentic standards were used for retention time and mass spectrometric data comparison. Chromatograms obtained from active enzymes are indicated in blue and the signal corresponding to the reaction product is highlighted in green.

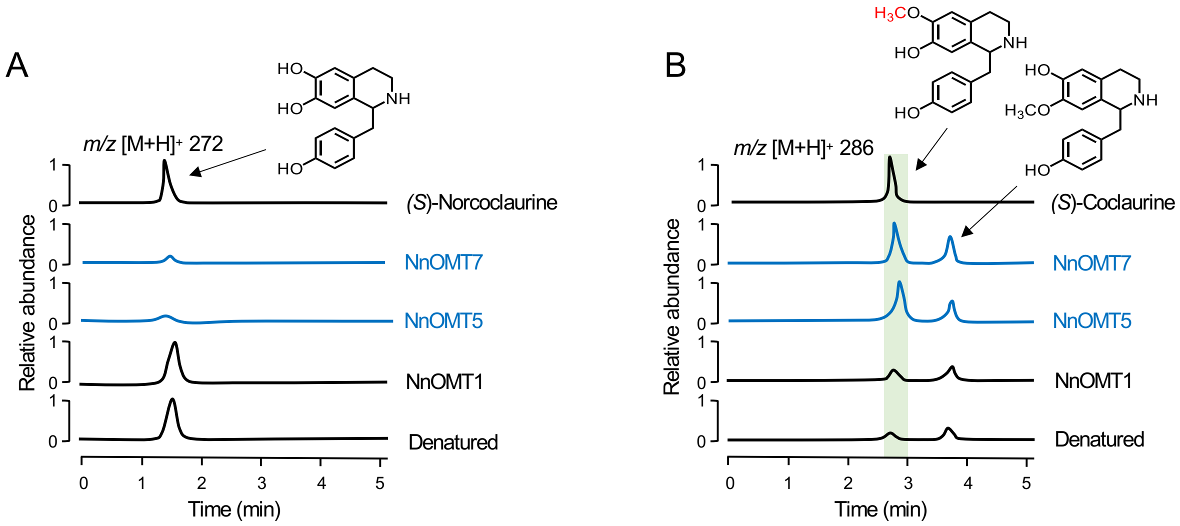

Supplementary Fig. 15: 6-*O*-Methyltransferase activity on sacred lotus folded leaf alkaloid extract. Chromatograms show variation in the signals for (**A**) norcoclaurine and (**B**) coclaurine when folded leaf alkaloid extract was incubated with sacred lotus OMTs. A peak corresponding to naturally occurring isococlaurine (Supplementary Table 8) was also detected for *m/z* $[M+H]^+$ 286 at 3.6 min. Authentic standards were used for retention time and mass spectrometric data comparison. Chromatograms obtained from active enzymes are indicated in blue and the signal corresponding to the reaction product is highlighted in green.

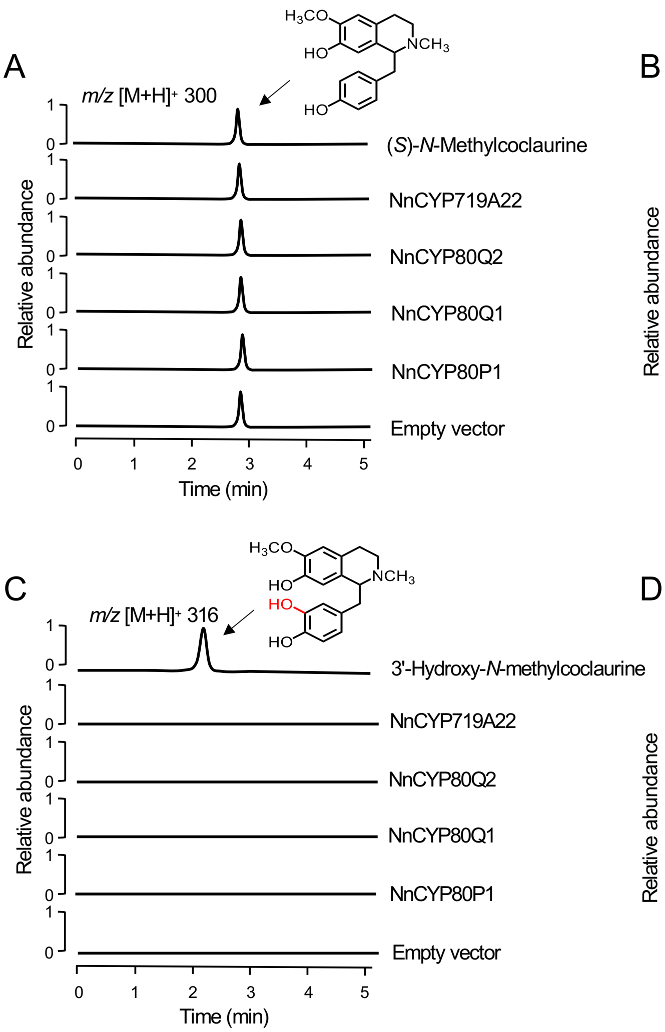

Supplementary Fig. 16: (*S*)-*N*-Methylcoclaurine is not a substrate for sacred lotus cytochromes P450. Chromatograms show (**A**) consumption of (*S*)-*N*-methylcoclaurine (*m/z* $[M+H]^+$ 300) and (**B-D**) absence of the expected reaction products: proaporphine *N*-methylcrotsparine (*m/z* $[M+H]^+$ 298), bisbenzylisoquinoline nelumboferine (*m/z* $[M+H]^+$ 597), and 1-benzylisoquinoline 3'-hydroxy-*N*-methylcoclaurine (*m/z* $[M+H]^+$ 316) in yeast strains expressing sacred lotus CYP candidates.

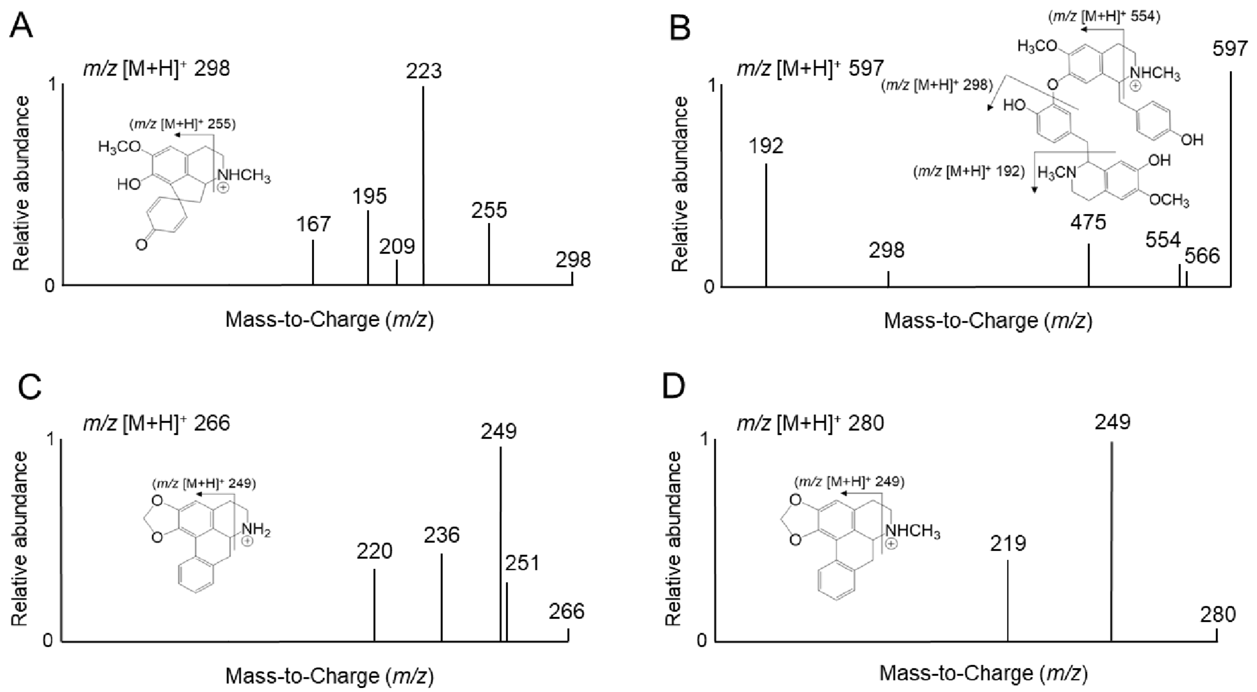

Supplementary Fig. 17: CID fragmentation of reaction products detected in yeast strains fed with sacred lotus alkaloid extract.

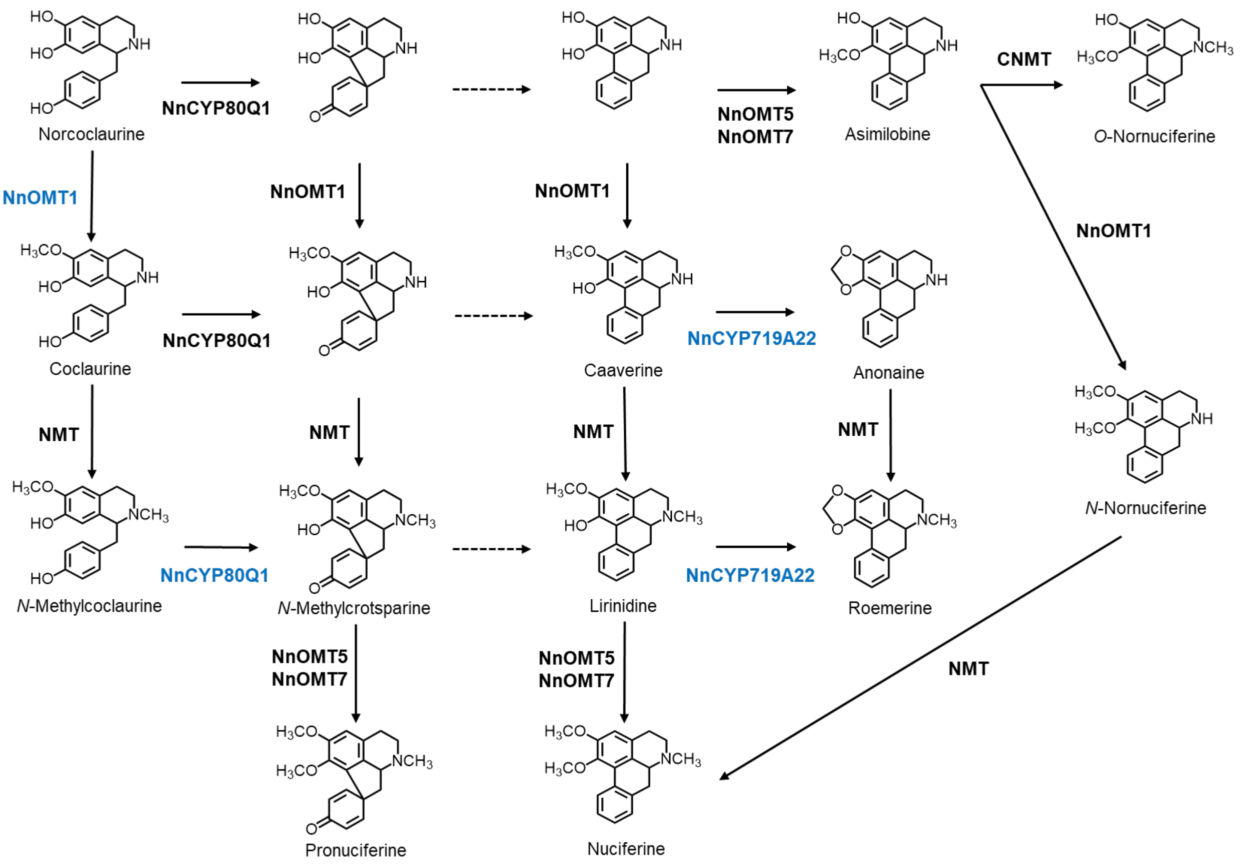

Supplementary Fig. 18: Proposed biosynthesis of proaporphines and aporphines in sacred lotus. Detected NnOMT1, NnCYP80Q1, and NnCYP719A22 activities are shown in blue, whereas expected activities are indicated in black. Only compounds previously reported in sacred lotus are named, including *N*-methylcrotsparine reported in this work. Dashed lines represent proposed reduction, dehydration, and aromatic ring rearrangement steps required to convert proaporphines into aporphines.

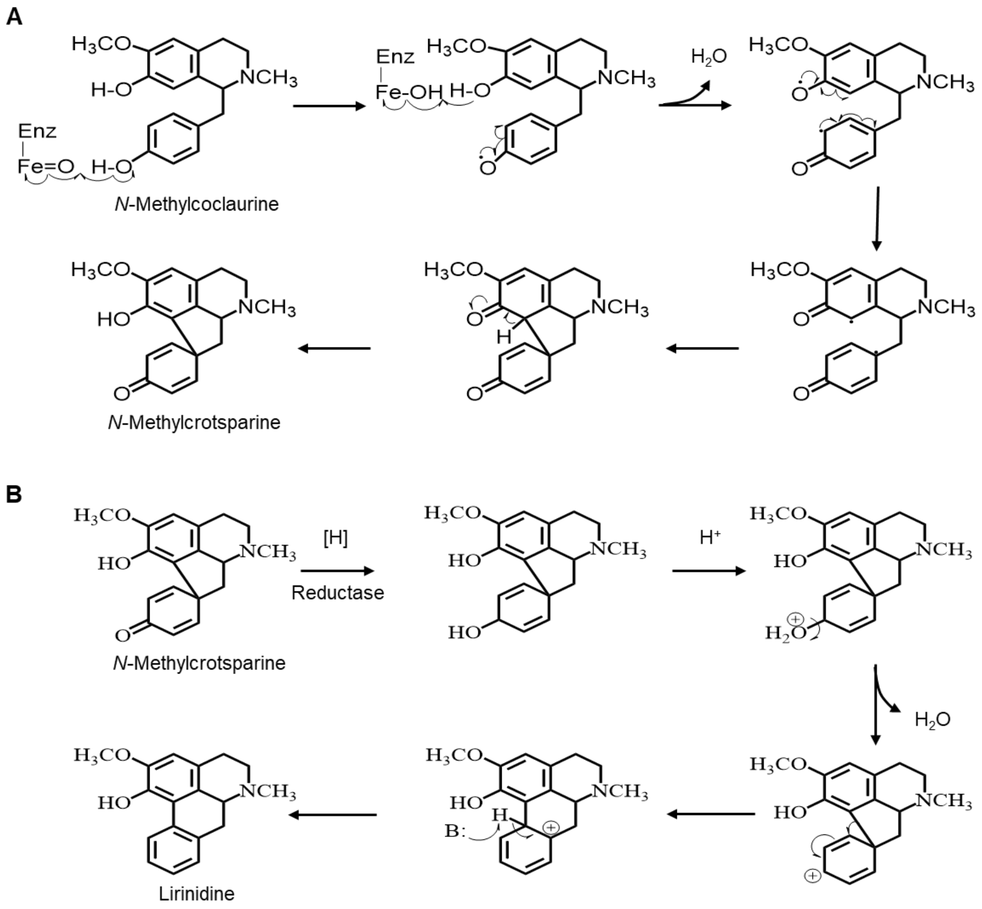

Supplementary Fig. 19: Proposed catalytic mechanism for proaporphine formation from a 1-benzylisoquinoline substrate, and subsequent conversion to a corresponding aporphine. (**A**) Schematic representation for the proposed biradical reaction mechanism corresponding to the intramolecular C-C phenol coupling catalyzed by NnCYP80Q1. An initial enzyme-catalyzed hydrogen abstraction occurs at the C4'-hydroxyl to generate a phenoxy radical and subsequent oxidation at the C7 hydroxy substituent generates the second phenoxy radical, concomitant with the removal of the reduced hydroxyl function as water. The resulting unpaired electrons are delocalized via resonance structures, forming radicals at C1' and C8, respectively, leading to the corresponding bond formation and the restitution of the C7 hydroxyl. (**B**) Suggested formation of corresponding aporphines by consecutive reduction of the proaporphine dienone to dienol, followed by dehydration and rearrangement to the fully aromatic ring. Parts of the figure have been adapted from previously reported schemes[1,2].

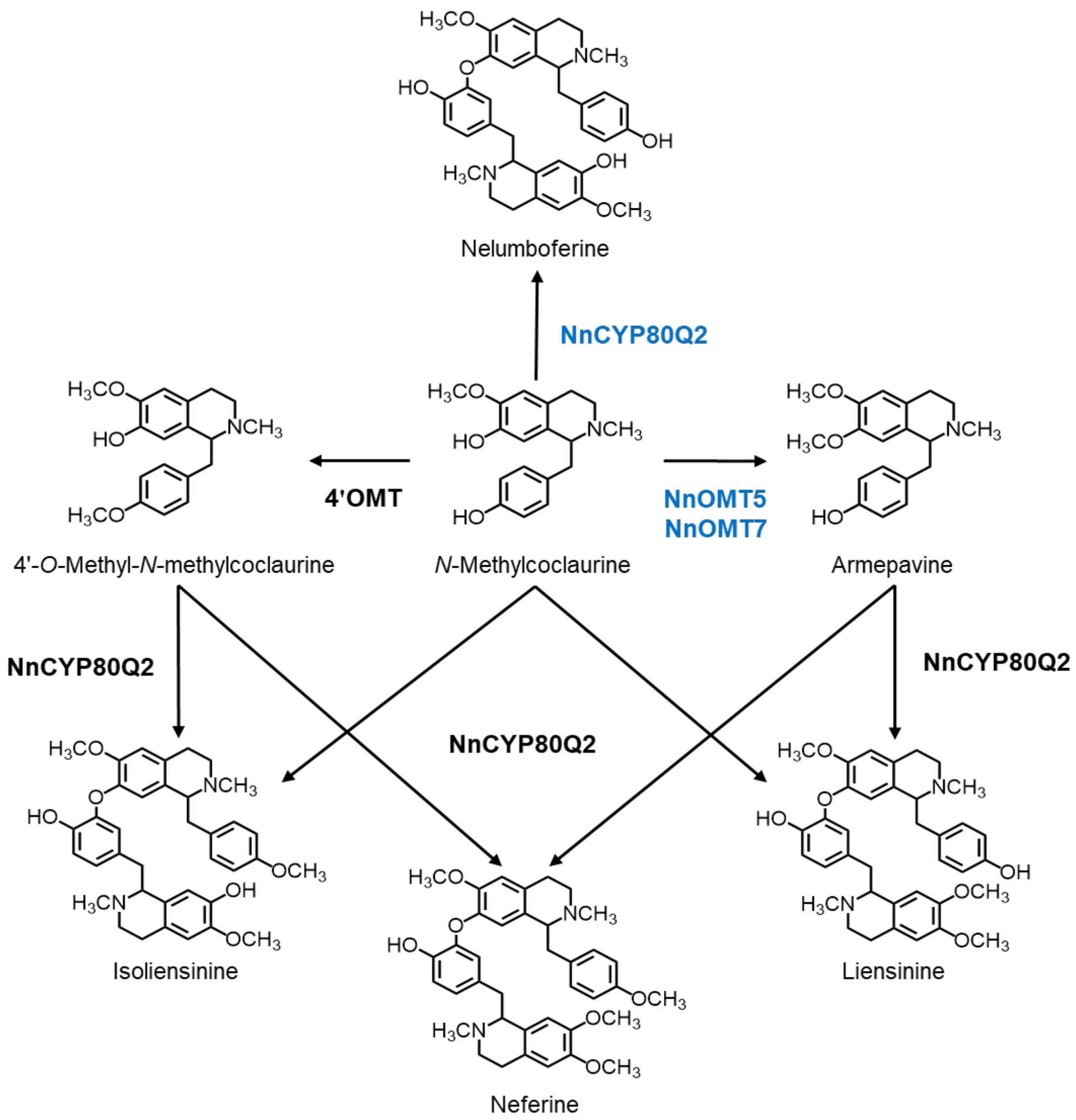

Supplementary Fig. 20: Schematic representation of bisbenzylisoquinoline alkaloids biosynthesis in sacred lotus. Detected activities of NnOMT5, NnOMT7, and NnCYP80Q2 are shown in blue, whereas expected conversions are indicated in black.

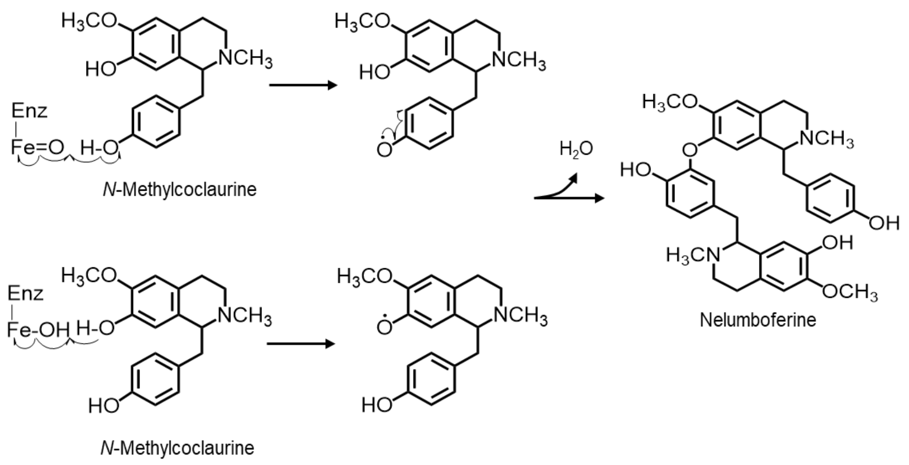

Supplementary Fig. 21: Proposed catalytic mechanism for the head-to-tail formation of bisbenzylisoquinolines in sacred lotus. Schematic representation of a biradical reaction mechanism corresponding to the intermolecular C-O phenol coupling catalyzed by NnCYP80Q2. The reaction begins with formation of the first radical at C4' and delocalization into C3' in the benzylic moiety of one *N*-methylcoclaurine molecule. A second radical is formed by hydrogen abstraction from the C7 hydroxyl in the second *N*-methylcoclaurine molecule, and both radicals react to form a biphenyl ether bond. Parts of the figure have been adapted from previously reported schemes[1,3].

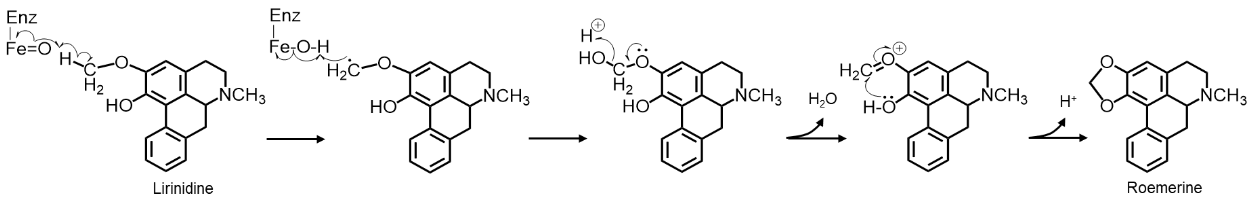

Supplementary Fig. 22: Proposed catalytic mechanism for methylenedioxy bridge formation in the isoquinoline moiety of aporphine alkaloids in sacred lotus. Schematic representation of a proposed reaction mechanism catalyzed by NnCYP719A22. The reaction is initiated by CYP-catalyzed hydrogen abstraction from the methoxy group, followed by enzyme-dependent hydroxylation and further cyclization via a methylene oxonium ion intermediate, and the associated release of water. Parts of the figure have been adapted from previously reported schemes[1,4].

Supplementary Information

Supplementary Table 1: Coding sequences of BIA biosynthetic gene candidates from sacred lotus. GenBank accession numbers are provided for each candidate.

Gene (GenBank ID)	mRNA coding sequence (5'-3')
**NCS**	
NnNCS1 (KT963033)	ATGATGATCGGACGTGTAGTTAACGAGATGGAGGTAGGCGTGCCTGCTGACGATAT<br>TTGGGCTGTGTATAGCTCGCCGGAGCTGCCCAGACTCTTCGTCCAGCTTATGCCCA<br>ATGTCTACAAGAAAATTGATATTCTTCAAGGTGATGGAACGGTCGGCACTGTCTTAC<br>ACATCGAACTCGCCGATGGGATACCGGAGCCACGTACATGGAAGGAGAAGTTCATA<br>AAGATTGATCATCAACACCGGGAGAAGGTAGTCCGACAAATTGAAGGAGGATTTCTT<br>GATATGGGGTTTCGTGTGTTCGATGTTATCTTCAAAATCATAGAGAAAGACGCCTGTT<br>CGTGCATCATTCGATCCACCACTGCTTTCGAGCTCGATGAGAAGTTCGAAAACAACG<br>CTAATCTTATTACTGCTGGTAATTTATGGGGAGCGGCCAAAGCAATTTCAAACTATGT<br>CATTCAGAACAAATCAAAGAGGAGAAACCACTAA
NnNCS3 (KT963034)	ATGCGTGGGCAAGTAACGAATGAATTAGATGTGGACGTGCCCGTCGACGATATCTG<br>GGCGGTGTACGGCACCCCTGTGCTACCCACCCACATCGTCCAGCTTCAGCCCGATG<br>TCTTTCAGAAGGTCGACTTTATCCATGGCAATGGAGGCGTTGGCACCATTCTATACG<br>TCCAACTTGTCCCAGGGGCACCGGAACCACGTACTTGGAAGGAGAAATTCATTAAG<br>ATAGACGACGAGGAGCGCTTAAAAGTGATACGAATGATCGAAGGAGGATACCTAGA<br>TCTTGGATTTACCTTGTTTGAGTACAACACACAAATTATAGAGAAAGACGCTGAATCG<br>TGCACAATCAGATCGACCACAGTTTTTGAAGTTGATGAGAAATTTGAGGCCAATGCG<br>GCTCTGATTAATGCTACTTCTGCATATGGATTGGCTAAGGCAGTTGCAAACTATGTCA<br>TTCAGAAGAAAGCTAAAGCTTGTGACGTCTAA
NnNCS4 (KT963035)	ATGCATGCTGGGCAACTATCACACGAACTAGAGGTAGCTGTGCCTGCCAGCGAGGT<br>GTGGGAAATCTATGGTACCCTGAAACTGGGGAAGGCTTGCGAAGAACTTCTCCCGG<br>ATGTCATCCACAAGGCAGAAGTAGTTGAAGGCGATGGAGGGGTTGGCACAGTGCTC<br>AAAGTTACACTTCCACCAGGTCTGATTTCTTATAAAGAGAAATTCACCAAGATTGATA<br>ATGAAAAGCGTTTGAAGGAGGTAGAAGTGGTGGAAGGAGGAGCCTTGGACCTTGGG<br>TTTCGTTTGTATCGAATTCGCTTGGAGATTATTGAGAAAACTGAAGTTTCATCGCTAA<br>TCAAATCAACCGTAGAGTATGAAATTGATGATGAGTCAGCCAACAACGCTTCGTTTG<br>CGACCACTAAGCCACTGGAGCAAATTGCAATGGCCATGGGGAAGTATCTCACTGAA<br>CTTAAAACAGAGTAG
NnNCS5 (KU234431)	ATGATTCACAGTGTGTCTACTGAATTGGAGGTTGATCTACCGGCTGATGATATCTGG<br>GCAGTATACAGCTCGCCGGAGCTGCCAAAGCTCGTCGTCAAATTGATGCCTCATGT<br>CTACGACAAGATTGACATCGTTGAAGGTGATGGTGGGGTTGGCACAGTTTTGCAGAT<br>CGTACTTACCCCAGAAATGATGGAGCCGCGTACATGGAAAGAGAAGTTCGTAGAGA<br>TAAATGACGGAAGGCGTAAGAAGGTGGTGCGACAAATCGAGGGAGGATATTTGGAC<br>ATGGGATTTCATTTCTATGAAGATATATTCAAAATCAAAAAGAAATCCGATTCATCGT<br>GCATCATTAAATCAAAATCGGTCTTTCGAGTTGACCACAAACACAAGGCCAATGCTT<br>CTCTAGTCACCCCCGATGCATCAGCGGAGATGGCCAAGGCAGTTGCTGAATATGCC<br>AAACAGAAGAAAGCTAATAGCAGCAGCAGCAGCAAAGATAAAGCTAAAGCATGCTAT<br>GAATGA
NnNCS7 (KU234432)	ATGATGACTGCGCGTGTAACTAACGAGATGGAAGTAGGGGTGCCTGCAGATGATGT<br>GTGGGCTGTGTATGGCTCGCCCGATCTGCCCAAACTCTTCGTCCAACTTATGCCCC<br>AAGTTTACAAGAGAAATGATGTTCTTGAGGGTGATGGAACGGTCGGCACTGTCATTC<br>TCATTGAACTCGACGACGCGCTTCCAGAGCCACGTATATGGAAGGAGAAGTTCATAA<br>AGATTGATCATCAAGAACGCGAGAAGTTGGTCCGAGTGATTGAAGGAGGATTTCTTG<br>ATATTGGGTTTCGTTCATTCGATATTATTTTCAAAGTCATAGAGAAAGACGCCTCCTC<br>ATGCATCATTCAATCCACCACTGCCTTTGAGCTCGACGACAAGTTTGAAGACAACGC<br>TAACCGTATTACCGCTGGTACTTTGTGGTGGGTGGCCAAAGCAATTTCAAACTATGT<br>CATACAGAACAAATCCAAATCCAAGAGCGACAACAACTAA
**OMT**	
NnOMT6 (XM_010276073)	ATGGAGTACAAGCAAGAATTGCAAAATCCCTTGATGGACCAAGCAATGGAGAAGGCA<br>AAGGAAGAGAAAATGGAGGAGTTGAGAGCACAGGTGCGCATCAGAAACTACATGAA

TGGTTATGCAGACTCAATGGCGTTGAAATGTGCTGTGGAGAATGGAATTGCAGACAT
CATCCAGAAACACGCCAAGCCCATAGCTCTCTCCGAGCTAGCGAAGGCTCTTCCCT
TGCCTACTGTTAGCACAGAGCACCTGGAGAGGCTGATGAAGTACTGGGTCCAATTG
GGACTCTTCACGCATGAGAAGGATGGCTCGCACGGGCTGACTCGGTGCTCTAAGTA
CTTGCTACGAGAAGAGAATACTACAATGGCCGCCATTATTCTTGGGCTAGTAACAGA
GTGGACCATAGGGCCATGGCACTGTTTGGCTAGAAGCTTGGAAGGAGGTCCGACG
GCATTCCGAAGATATCATGGGAGGGATATGTGGGATTATGTGGCTGGTCACCCGGA
GGCGAGTCGACTGTTCAACGAGTCTATGGCAGGCGATACAAGACTGTTGTTGCCTG
TGCTGATGCAAGAATGTGGGTCATGGTTGTTTGAAGGGATTTCGTCGTTGGTGGAC
GTGGGAGGAGGGAATGGGACGGCCATGGTGGAGATAGCAAAGAAGTTTCCGGGAA
TTAAGTGCACTGTGTTTGATCTCCCACACGTGATAAGGAACACGAACTCGAGTGAGT
CAACTGGGGTGGAATGGGTTGAAGGGGACATGTTTGAATCAATACCTCCAGCAGAT
GCAGTTTTGCTCAGAGAGGCAATTCCCAAAGAGCGAGGGAAAGTAATCATCATCGA
CATAGTAATGGATATTGAGCAGGATCCTGAGTTAATCCGGGCAAAGTTGATGACAGA
TATTGACATGATGTCCGATGATCTAACAGTGATGGATCAGAGACATCACGAGGCCCA
GGCATTATCCAGATTGGCAGTAAAGGCCCCAAACCCCCTTCTTCGAAGATGGCATTA
CGATAGGAATCATCAGAGGTTGCATGAACAAAGTCAACCACCGCAGCTTGCTCTCAG
TGATTACCTTGACTGCGATAATGTGTGTAGCTGA

NnOMT7 (XM_019200018)

ATGGAGGACATGAAAGCTCAAGCTCAAGTGTGGAAACACATATACGGCTTCGCCGA
GTCACTCGCTCTCAAATGCGCGATCGAACTCGGGATCGCGGACATACTCTACGAAC
ATGGTCAGCCCATGACTCTCTCCGAGTTAGCCTCCTCCATCCCTCTTCCCTCGGTCA
GCCAAGACGGATTGTACAGGGTTCTGCGTTACCTCGTCCACATGAAACTCTTCGACC
TGCAGGTCGATTCCGACGGGTTAAAGAAGTACTGGCTCACTCCCGCGTCCAAGCTC
TTGGTCAAAAGCCAAGAGAAGAACCTCGCATCCTTCGCTCTCCTGATCTTCTACGAG
ATGGACGCTTGGCACCACCTAAGTGCCGCAGTGGAAGGTACCGTGACACCCTTTGA
GAAGTGTCACGAGGGCGAGGACCTCGAGGAATTATTTGGCAAAGACTCGGTGATCA
ATCGATTGTTGAGCGAGGGGATGACGAATCTAACGAGTTTGATGGCGGACGCGCTG
GTGAAGGGCTGCAAGAAGGCGCACATCCTTGATGGAGTAGGGTCCCTAATTGACGT
GGGCGGGAGTACTGGGGTAGCTGCGCGTGCCATCGCTAAGTCGTTCCCCAGTATAA
AATGTGCGGTGCTTGATCTGCCCCACGTAGTAGCCAACGCGCCCGAGTGTTCCGAG
GTGACTTGGATCGAAGGCGATATGTTTGTATCCATTCCGAAAACGGATGTGGTGTTC
ATGAAGGTGAGGTCGGTCTTACACGACTGTGGAGACGAAGACTCAGTGAAGATTCT
GAAGATATGCAAGGAGGCCATATCGGAGAAAGGAGGAAAAGTAGTGATCGTGGAGA
TTGTCATGGATGTAGAATCATCTTCATCACCGAACGAGATTACCGGTGCAAAGCTGA
ATCTGGACATGTCTATGCTGGTCACACCCGGTGGTAAAGAAAGAAGCGAAGAAGAC
TGGCAGAAGCTCTTCAAGGAGGCCGGTTACAGCCGGTACAAGATCACGCCCATCGC
TGCATTCGAATCAATTATAGAAGTCTTCCCTTGA

NnOMT8 (XM_010274082)

ATGGGGCAAGGCGACGAGATGGAATTGGAGGCTCAGGCTCAGATATGGAAATACAT
CTTCTCCTTTGTTGATTCCTTTACTCTGAGGTCTGCAGTTGAACTAGGAATCCCAGAC
ATCATACATAGTCAGGGGCGTCCCATCACACTATCACAGCTCTCTGCATGTCTCCCG
ATCGATTATGCCAACCCAGATCGCTTGAATCGCTTAATGCGATATCTGGTGAGCATA
GGAATCTTCAGTCGAGAACATGGTAGCGCTGATGATGAAGATGATCAAGAAGACAA
GTTTGGACTAACTTGTCTATCAAAACTTCTAGTACGAAAGCTGGAGAACAATATGGTT
CCATTCTCCATGTTGGACTTCAAGGTGTTAATGGAGCCATGGTATCACTTGACATGG
AGCTTGGATGGCAGAGCAAGCGGCGTTACAGCATTTGAGAGAGTTTATGGGACAAA
ATTTTGGGATTATGCAGGGCAGGATCTGGAGTTTGGTGAGAAGCTGAATGAGGCCA
TGGCTTGTGATACAAGCTCAACGATGCCTACACTACTCCAACAATTCAATCAAGTGTT
TGCAGACATGAGTTCAGTCGTAGATGTTGGTGGGGGGACTGGCACGGCTGCTATGG
CCATAGCCAAGTCCTTTCCAAATGTGAAGTGCACCGTCTTTGACCATCCACATGTGG
TAGTGGACGATCAATCAGACTCTGGTGGTGTGGCCAAGGTCTCCGGCGACATGTTC
AACTTCATCCCTCGTGCAGATAATCTTCTATTAAAGTGGATCCTGCATAACTATGGAG
ACGAAGACTGTCTGGTTATCCTCAAACGGTGCAAGGAAGCACTGCCAGAAAGGGGA
GGAGGAAAGGTTGTAATCGTTGAAACTGTGATGGAGGGTGATCACAATGATGAAACT
GCAGCGGAGATCCATGATGATGAATTTACACCTTGTAGACTTATCATGGACATGGAA
ATGATGTTGCTGTTCGGGGGTAAAGAGAGGACTGCCAAAGAATGGCAGAGTCTGGT
TGAACGAGCTGGGTTTAGGCGCTGCACAATGGAACGAATCAACTCATCTATGCACTC
TCTAATTCAGGCCTATCCTTGA

Supplementary Information

CNMT

NnCNMT (XM_010263690)

ATGGATGCGTTGATCCAGGTACCATACGATGCAACTATACGTTTAATGCTGTCGTCT
CTCGAGCGTAACCTCCTCCCCGACGTCGTCATAAGGAGGCTCACGCGGCTGCTGTT
GGCTAGCCGTCTTCGTTGGGGATACAAGCCGTCCTCTCAACTCCAACTTTCTGATCT
TCTCCAATTTGTTCACTCGCTAAAAGAAATGCCCATTGCCATCAAGACCGACTTACCA
AAGTCCCAACATTATGAATTACCCACTTCCTTCTTCAAGCTGGTTTTAGGGAAGAATC
TCAAATACAGCTGCTGTTACTTCCTTGACAAGTCAAGCACCTTAGAGGATGCAGAGA
AAGCTATGCTGGAGCTGTACTGTGAGAGGGCACAGATCAAAGATGGCCAATCTGTG
CTTGATGTTGGTTGTGGCTGGGGATCATTGTCCTTGTATATTGCACAAAAGTTTTCTA
GCTGCAGGATAACAGGGATTTGCAATTCAAAGACACAGAAAGCATATATAGAGGAGC
AATGTAGGGAACTGAAGCTGCAAAATGTGGAGATCATTGTTGCAGATATCAGCACTT
TTGAAATGGAGGCATCATTTGATAGGATTTTATCCATAGAAATGTTTGAACACATGAA
GAACTACAAGGCACTTCTTAATAAGATATCAAAATGGATGAAGGAGGATAGCCTCCT
TTTTGTTAACTACTTCTGCCATAAAGCATTTGCTTACCACTTTGAGGACAAGAATGAA
GATGACTGGATTACCAGGTACTTCTTCACTGGAGGGACAATGCCTGCTGCAAACCTT
CTCCTCTATTTCCAGGATGATGTTTCTGTTGTCAACCATTGGCTTGTAAATGGGAACC
ATTATGCAAGAACAAGTGAGGAGTGGCTTAAAAGAATGGACCAGAACATGGCTTCTA
TTAAGCCAATAATGGAGTCAACTTATGGCAAGGATTCCGCTGTTAAGTGGACTGCCT
ATTGGCGTACATTCTTCATCTCAGTGGCAGAACTGTTTGGCTATAACAATGGAGAAG
AATGGATGGTTGCACTGTTCCTATTCAAGAAAAAAATAAATTAA

CYP

NnCYP80P1 (XP_010253989)

ATGGAAATCATGGCTCAAGCAGCTTTAGCAGGAGAGGTCATCAATCTCTTATTTCCT
GTCTTCCTCTCACTTTGCCTCTTCTTTCTCTTCATCAAGCTCATTAAATCTGCATCATC
AGCTACAGGTCCACCACTTCCACCAGGCCCACATCCATGGCCTGTCGTCGGCAACA
TTTTTCGCCTGGGAAGGAAGCCCCATGTTAGACTCAGCCAATTAGCGCAAGTTCATG
GTCCTCTCATGTCACTAAGTCTAGGCAGACAACTTATCGTTGTTGCATCATCGCCTG
CAACTGCAACCCAAATTCTCAAGACCCATGACAAAATACTCTGTGGTCGCTATGCTC
CGGTCTCTAGCAGACGCAATGATCAATTGAAGTATCTGGATAACTTCTTGTGGACTG
AGTTGTTCTCAACCAGAGCAATTGATTCTCAGGCAGCTTTGAGGGAGAAGAAGGTCA
GGGAGCTGATACGCTATCTGGGTTCAAAGGAAGGGGAAGTAGTGAACATTGGAGAA
GTAATGTTTGCTACTGTTTTCAACATCCTGTGTAATCTTTTTCTGTCAAAAGACTTCAT
TAGCCTGGAGGATGATGAGATTATGAAGGGAGGGATTAAGAGACTTCTGAGGAGTA
TTTCGGAGGTGGCGTCTACTCCAAACTTGGCTGATCTTTTCCCCATATTAGGTCCATT
GGATATTCAAGGGCTAAACAAGAAGGCTAGAGAACTGTTTGTGAAAATTACTGCCAT
GTGGGAAGATATCATCAAAGAAAGAAGAGAAGCACAGAGTGCTGGTGGCCATGTTT
CAAGGCAAAGGGATCTTCTGGATGTTCTAATCGACAACAACTTCAGTGATGATCAGA
TCAACAATTTACTACTGAAGTTGTTCACTGCTGGTACACACACTAGTAGCTCAACAAT
TGAATGGGCAATGGCGGAGATGATGAAAAATCAAGAATCCCTGGTCAAAGCTCGTAT
AGAACTAGCAAGAGAGATCAAAAGAGAAAACCAAGTAAGAGAGGCTGATCTCTGTAA
CCTAGTATACCTGAATGCCTGTCTGAAAGAGACACTGAGACTACACCCTCCTGCGCC
ATTCCTCCTCCCTCACCGAGCCATCAAAACATGCACAGTGATGAATTACACCATTCC
AAAGGACTCTCAGGTCTTTGTGAACGTATGGGCAATTGGGCGGGACTCCATGGCTT
GGAGCAACCCATTATCCTTCAATCCAGAGCGCTTCCTCTCGTCAAATTTGGGTTTCA
TGGGGAATAATTTTGAGTTCATACCGTTTGGTGCTGGAAGGAGAATCTGCCCGGGA
CTACCCATGGCTGGCAAGCAAATTCAACTAATCATGGCCTCCTTGATCTACTGCTGT
AATTGGTCTCTCCCAAATGGCACTCACCCATCCACGCTAAACATGAATGACAAGTTG
GGGGTTGTGTTGCAGAGGGAACAACCTCTACTCCTCATTCCTAAACTGAGAAGGAG
AAATTATTAA

NnCYP80Q1 (MG517489)

ATGGCTCTACTAGTCTCGTTTATTTTCTTCTTACTGTCCATTCTCTCAGTAGTACTCTT
CCTTAAACCATCTCCTAATAACCTTCCCCCAGGACCCTTTTCATGGCCAATTATAGGG
ACCCTGTTGCCCAAGCTGAAGAAGCAACCCCATGTCGAGCTAAGCAAATTGGCACA
GAGATTTGGTCCACTAATGCTCCTAAAATTTGGGGTTGAGCCCGTTGTTGTGGCTTC
GAGCCATGTTGCAGCCGTGGAGGTGCTCAAGAATCAGGATCGATTGCTTTCAGGCC
GCTTTGCACCACACAGCGTCCGAATTAAGGGCTATATTGAACACTCCATGGTGTGGG
CTGATTGCACTGATTACTGGAAGATGGTCAGGAAGGTATGGAGGACTGAATTGTTCT
CTACTAAGATGTTGGACATTCAGGCACACGCCAGAGAGGAGAAAGTATCGGAACTG
ATGAAATTTCTCATACGGAAGGAAGGAGAGAAGGTGAATTTTGCTGATGTGATATTC

GGTTCCATTTTGAACATATTAGGTGCACTTATTTTCTCCAAAGACGTGTATGATTTTG
AAGACAGGACGGATAATAACTTGGGTATGAAGGGCATGATTCGGCAGCTGATGATAT
TGGCAGCCATCCCAAATCTAGCCGACCTCTACCCAATTCTTGGCGGATCAGACTTTC
AGGGCTTGAGGAAGGCATCGGCAGCGTGTGTCAAGCGGATGAATGAGTCATGGGC
CGCCATTGTTAAACAAAGGAGGAAGAATGATGACCACTCCAAGAATGATTTCTTGCA
AGTTTTGCTCGATTCTGGGTTCAGTGATCCCCAGATTGACGCCATGCTTCTGGAAAC
GTTTGGACCTGGTTCAGACACTAGTACCTCCACGATTGAATGGGCAATGGCAGAATT
GCTGCGGAACCCAGAGAAGCTGGTAAAGGTCCGCGAGGAACTCGACAGGGTGATC
AGAAGAAGCAACAATGTGAAGGAGTCTGATCTGCCAAACCTACCTTATCTCCATGCC
TGTGTCAAAGAGACCCTCAGGTTACACCCTCCGGTCACCTTCCTTCTTCCACACCGA
GCAATGGAAACTTGTCAAATGATGAATTACACGATTCCAAAGGGATGCCAACTGATG
GTAAACACATATGCAATTGGAAGAGATTCCAAGACATGGGAGAAACCCTTGTCTTTC
TTGCCAGAACGATTTCTGAACTCAGAACTTGATTACCAAGGTAACGATTTCCAGTACA
TACCATTTGGTGCCGGCAGAAGAATCTGTCCAGGGTTGTCATTGGCAACTCGAGTTG
TTCGACTGATACTCGCTTCTCTTCTCCACACTTTTGATTGGAGCCTTCCTGATGGAAT
GCACCCAGATGAGCTAGACATGAACGATAAGTTTGGGCTGGCTCTCCAGAAGGACA
TCCCTCTGGTAGTCATTCCCAAGTTGAGGAAGTAA

NnCYP80Q2
(XM_010255687)

ATGGCTCTACTAGCCTTGTTTATTCTCTTCTTACTGTCCATTCTCTCACTAGTACTCTT
CCTTAAACCATCTTCTAAGAAGCTTCCTCCAGGACCCTTTTCATGGCCAATTATAGGA
ACCCAGTTGCCCAGCCCGACGATGAAGCCCAATTTGGAGCTATTCAAATTAGCACAG
AGATATGGTCCACTGATGCTTTTCAGATTTGGGTTTGAGAACGTTGTTGTGGCTTCAA
ACCATGTTGCAGCCATGGAGGTGCTCAAGAACCAGGACCGCGTGCTTTCAGGCCGG
TTTAAAGCAAACAGCGTCCGAGTTAAGGGCTATATCGAATACTCCATGGTGTGGGCT
GATTGCACTGATTACTGGAAGATGGTCAGGAAGATATTGAGGACTGAATTGTTCTCT
ACTAAGATGTTGGACGTTCACGCACACGTCAGAGAGGAGAAAGTATCGGAACTGAT
GAAATTTCTCAGACGGAAGGAAGGAGAGGAGGTGAATTTTGTTGATGTGATATTCGG
TTGTATTTTGAACATGTTGGGTGCACTTATATACTCTAAAGACGTGTATGATTTTGAA
GACAAGACGGATATTAACTTGGGTATGAAGGGCATGATTCGGCAGCTGATGATATTG
GCAGCCACCCCAAATATAGCCGACCTCTACCCAATATTTTTTGACGGATCAGACTTT
CAGGGACTGAGGAAGGAATCGGCAGCTTGTGTCAAGCGGATGAGTGAGTCTTGGG
CGGCCATTATTAATGAAAGAAGGAAGAATAATGACCACACCAAGAACGATTTATTGC
AAGTTTTGCTAGATTCTGGGTTCAGTGATCCCCAGATTGACGCCATTTTTCTGGAAAC
GTTTGGACCTGGTTCAGACACTAGTGCCTCCACGATTGAATGGGCATTGGCAGAACT
GCTGCGGAACCCAGAGAAGCTGGTGAAGCTCCACGAGGAACTCGACAGGGTAATC
GGAAGAAACAACACTGTGAAGGACTCTGATCTGCCTAACCTACCTTATCTCCATGCC
TGTGTCAAGGAGACCCTCAGGTTACACCCTCCGGTCCCATTCCTTATTCCTCACATA
GCATTGGAAAGTTGTGAAGTGATGAATTACACGATTCCAAAGGGATCCGAAGTGTTG
GTTAACTTATATGCAATTGGAAGAGACCCCACCACATGGGATAACCCCAATTCTTTCT
TGCCAGAACGCTTTCTGAATTCAGAAGTTGATTACCAAGGCAACCATTTCCAGTACAT
ACCCTTTGGTGCTGGCAGAAGAATGTGTCCTGGCATGTCATTGGGAACCCGAGTTG
TTCGACTGATACTTGCTGCTCTTGTCCACACTTTTGATTGGAGCCTTCCTGGCGGGA
TGCACCAAGATGAGTTAGACATGGCCGATAGGTTTGGGGTGGGTTTCCAGAAAGAA
ACCCCTCTCGTCGTCATTCCCACCTTGAGAAAATAA

NnCYP719A22
(XM_010268782)

ATGCAGGGCAATCAGGGTTTAATTCTTGCCAGTGTAATCTTCGTGGTCGCAATTGTA
CAGATGATGATTCGAAAGCGAAGAACGTCGCCCACTGCCATGAAATGGCCGGCAGG
ACCCAGGAAATTGCCCATCATAGGTAACATGCACCAACTATCTAGAGCCGATGGCTT
GTTTCATGTGGCCCTAACCAAACTGGCCAAAGTTCATGGGAGTGTGATGACCATCTG
GCTTGGTAGCTGGCGGCCCACTATTGTTGTGAGCGACGACGAGGTGGCTTGGGAG
GTGTTGGTGAACAAGTCCTCAGACTACGCTGCTCGTGATCATCCTTACATTGACAAG
ATCATGTGGGCAGGGGCTCGCACCATCCACACATCTGACGCCAGCCCCCACTGGCA
CAGTCTTCGCAAGGGCCTTCAAAGCGGTGGCCTAGGCCCCCTCAGCATCTCCGGCC
AAACCCACTTGCAAGAAAAAGATATTGCACAGATGCTCCGAGACATGCGTGAAGAG
GCCTCCCTCAACGGTGGCCTTGTCAAGCCTTTTCACCATATTCGCCGTACCTCGGTG
CGTTTACTATGCCGCCTTTGCTTCGGCCCCAACTTTGAAGATGCCAAGTTCTCCGAA
GCAATTGATAAAGCCATTGAAGATATCATCCGCATCAGTGGCGTTGGCTATCTGGCC
GACGCCTTCTTCTTCGGCCGACACTTCCCCGGCCTCAAGCACACATTCCAGGAGGC
ATGCGACCTGAAACGCCGAGTCGAGGATTTGATACGCCCTTTTCTCAGAGCAGTTCC
TCCTCCAAACTGCTATTTGCACTTCCTTCTTTCCAATAACATCCCCGAAGATGTCACC

ATATTCACCATCTTAGAGGTATTCACACTCGGCATCGATAGCACCTCCTCCACCGCA
ACTTGGGCACTTGCTCTTTTGACCAATGAGCAACGAGTCCAACAAAAACTCTACCAA
GACATCAAGAAGAACATAGACAGTACCCAACAAATTGTGAGGGTTGAGGATGTAAGC
AAGCTGCAATATTTACAAGCGGCTGTGAAGGAGACGTTGAGATTGAAGCCTGTTGCT
CCTTTGGTGCCTCACATGACTGCCACAGAGACCACTCTGATGGGAACAAAGGTGGC
ACAAGGTACCAGAGTGGTGGTAAACCTTCACGCTATACACTATAATCCAAATGTGTG
GCCAGAACCGGAGAAGTACATGCCTGAGCGGTTCATGCCTCGTCAAGAAGAGGTTG
ATGAAATACGGCCGGGAACCACGAAACTATCTTATTTCCTTCCATTTGGAGGTGGAA
TGAGAGCTTGTGCAGGAATGGAGGTAGGGAAGCTTCATGTAGGGTTTGTAATAGCT
AACATAGTTAATGCCTTTCAATGGTCTAGTGCTGTCGAGGGGCAACCCCCCGATTTG
ACCGAAGACTTCAAGTTTGTGCTCCTCATGAAAAATCCACTTACAGTGCGCATCACT
GCTCGTCACCCTTGA

Supplementary Table 2: Predicted molecular masses and isoelectric points (pI) of biosynthetic enzyme candidates from sacred lotus. Abbreviations: CNMT: coclaurine *N*-methyltransferase; CYP: cytochrome P450 monooxygenase; NCS: norcoclaurine synthase; OMT: *O*-methyltransferase.

Enzyme	Mass (kDa)	pI
**NCS**		
NnNCS1	18.5	6.4
NnNCS3	18.0	4.7
NnNCS4	17.1	4.8
NnNCS5	19.2	7.7
NnNCS7	18.6	4.8
**OMT**		
NnOMT6	41.6	5.4
NnOMT7	38.2	5.1
NnOMT8	41.1	4.8
**CNMT**		
NnCNMT	41.7	7.0
**CYP**		
NnCYP80P1	55.8	9.3
NnCYP80Q1	55.1	8.9
NnCYP80Q2	55.2	6.6
NnCYP719A22	55.7	9.2

Supplementary Table 3: Amino acid percent identity matrix for sacred lotus norcoclaurine synthase (NnNCS) candidates and several functionally characterized, single-domain NCS enzymes involved in BIA biosynthesis in the Ranunculales. Proteins sharing more than 40% amino acid sequence identity are highlighted. Abbreviations: Cj, *Coptis japonica*; Nd, *Nandina domestica*; Nn, *Nelumbo nucifera*; Ps, *Papaver somniferum*; Sc, *Sanguinaria canadensis*; Tf, *Thalictrum flavum*; Xs, *Xanthorhiza simplicissima.*

NCS	NnNCS1	NnNCS3	NnNCS4	NnNCS5	NnNCS7
TfNCS	38	40	41	36	37
TfNCS2	39	42	42	38	38
PsNCS1	41	41	39	38	36
PsNCS2	39	40	41	39	34
ScNCS1	41	39	41	39	40
NdNCS3	36	39	43	40	34
XsNCS1	29	31	34	30	29
CjPR10A	32	32	38	32	32
NnNCS7	77	54	42	50	
NnNCS5	56	51	38		
NnNCS4	40	42			
NnNCS3	60				

Supplementary Information

Supplementary Table 4: Amino acid percent identity matrix for sacred lotus *O*-methyltransferase (NnOMT) candidates and several functionally characterized OMTs involved in BIA biosynthesis in the Ranunculales. Proteins sharing more than 40% amino acid sequence identity are highlighted. Abbreviations: Cc, *Coptis chinensis*; Cj, *C. japonica*; Ct, *C. teeta*; Ec, *Eschscholzia californica*; Gf, *Glaucium flavum*; Nn, *Nelumbo nucifera*; Ps, *Papaver somniferum*; Tf, *Thalictrum flavum*; 4'OMT, 3'-hydroxy-*N*-methylcoclaurine 4'-*O*-methyltransferase; 6OMT, norcoclaurine 6-*O*-methyltransferase; 7OMT, reticuline 7-*O*-methyltransferase; CoOMT, columbamine *O*-methyltransferase; N7OMT, norreticuline 7-*O*-methyltransferase; SOMT, scoulerine 9-*O*-methyltransferase.

Type	OMT	NnOMT1 (6OMT)	NnOMT2	NnOMT3	NnOMT4	NnOMT5 (7OMT)	NnOMT6	NnOMT7 (7OMT)	NnOMT8
6OMT	Tf6OMT	69	64	62	62	47	40	48	41
	Ps6OMT	63	58	57	59	46	40	45	42
	Cj6OMT	69	64	63	62	47	41	48	42
	GfOMT2	69	64	63	63	48	40	47	42
	Cc6OMT1	43	42	41	42	43	40	42	37
	Cc6OMT2	69	64	63	62	46	41	47	42
7OMT	Ps7OMT	41	42	40	38	42	35	40	41
	PsN7OMT	55	53	50	51	44	36	42	40
	Ec7OMT	43	43	42	41	41	35	38	38
	Ct7OMT	43	42	41	39	38	37	38	40
4'OMT	Ps4'OMT2	52	50	49	49	42	39	41	41
	PsOMT2	41	39	38	40	42	36	43	37
	PsOMT3	59	53	52	54	44	36	44	39
	Cj4'OMT	54	51	51	50	45	39	44	39
	Ec4'OMT	52	50	51	50	43	37	44	40
	GfOMT1	54	51	51	50	43	39	43	38
SOMT	PsSOMT1	32	31	31	29	31	25	30	29
	EcSOMT	42	41	39	38	43	35	42	39
	CjSOMT	34	33	32	31	30	23	30	30
	CtSOMT	35	33	33	32	29	25	29	31
	GfOMT6	35	34	33	32	29	25	28	33
	GfOMT7	36	36	34	32	34	27	33	32
CoOMT	CjCoOMT	42	40	39	40	46	31	43	42
-	NnOMT8	45	43	44	43	42	40	41	
-	NnOMT7	47	44	45	45	85	39		
	NnOMT6	44	44	43	44	37			
-	NnOMT5	46	44	44	45				
-	NnOMT4	81	77	74					
-	NnOMT3	83	84						
-	NnOMT2	84							

Supplementary Table 5: Amino acid percent identity matrix for *Nelumbo nucifera* coclaurine *N*-methyltransferase (NnCNMT) candidate and several functionally characterized NMTs involved in BIA biosynthesis in the Ranunculales. Proteins sharing more than 40% amino acid sequence identity are highlighted. Abbreviations: Cj, *Coptis japonica*; Ec, *Eschscholzia californica*; Gf, *Glaucium flavum*; Nn, *Nelumbo nucifera*; Pb, *Papaver bracteatum*; Ps, *P. somniferum*; Si, *Stephania intermedia*; Tf, *Thalictrum flavum*. NMT, *N*-methyltransferase; PavNMT, pavine *N*-methyltransferase; RNMT, reticuline *N*-methyltransferase; TNMT, tetrahydroprotoberberine *N*-methyltransferase.

Type	NMT	NnCNMT
CNMT	PsCNMT	52
	CjCNMT	54
	GfCNMT	53
	GfNMT6	55
	TfCNMT	53
	SiCNMT1	80
	SiCNMT3	48
RNMT	PsRNMT	44
	GfNMT4	47
	GfNMT5	50
	TfPavNMT	47
	SiCNMT2	54
TNMT	PsTNMT	46
	GfTNMT	45
	GfNMT3	47
	PbTNMT	46
	EcTNMT	46

Supplementary Table 6: Amino acid percent identity matrix for *Nelumbo nucifera* cytochrome P450 (NnCYP80) candidates and several functionally characterized CYP80s from BIA-accumulating species in the Ranunculales. CYP80s sharing more than 40 % amino acid sequence identity are highlighted. Bs, *Berberis stolonifera*; Cj, *Coptis japonica*; Cy, *Corydalis yanhusuo*; Ec, *Eschscholzia californica*; Nn, *Nelumbo nucifera*; Ps, *Papaver somniferum*; Tf, *Thalictrum flavum.* NMCH: *N*-methylcoclaurine 3'-hydroxylase.

Type	CYP80	NnCYP80P1	NnCYP80Q1	NnCYP80Q2
3'-Hydroxylase	PsNMCH	45	49	45
	EcNMCH	45	50	47
	TfNMCH	45	49	46
	CjNMCH	48	52	48
	CyNMCH	48	51	46
Aporphine synthase	CjCYP80G2	45	49	48
Bisbenzylisoquinoline synthase	BsCYP80A1	37	47	45
	NnCYP80Q2	46	80	
Proaporphine synthase	NnCYP80Q1	50		

Supplementary Table 7: Amino acid percent identity matrix for *Nelumbo nucifera* cytochrome P450 (NnCYP719A) candidate and several functionally characterized CYP719As from BIA-accumulating species in the Ranunculales. CYP719As sharing more than 40 % amino acid sequence identity are highlighted. Am, *Argemone mexicana*; Cc, *Coptis chinensis*; Cj, *C. japonica*; Cm, *Chelidonium majus*; Ec, *Eschscholzia californica*; Nn, *Nelumbo nucifera*; Ps, *Papaver somniferum.*

Type	CYP719	NnCYP719A22
Canadine synthase	CjCYP719A1	55
	PsCYP719A21	50
	CcCYP719A	55
Stylopine synthase	EcCYP719A2	49
	EcCYP719A3	49
	AmCYP719A13	48
	PsCYP719A20	49
	CmCYP719_SPS	49
Cheilanthifoline synthase	EcCYP719A5	50
	AmCYP719A14	49
	PsCYP719A25	48
	CmCYP719A_CFS	52
1-BIA methylenedioxy synthase	EcCYP719A9	48

Supplementary Table 8: Chromatographic and mass spectral data for BIAs detected in enzyme assays, in engineered yeast, and in sacred lotus plants. Abbreviation: AS, authentic standard.

Alkaloid	Elemental Formula	*m/z* $[M + H]^+$	Rt (min)	Major product ions *m/z*	Reference CID
**1-Benzylisoquinolines**					
4'-Deoxynorcoclaurine	$C_{16}H_{17}NO_2$	256	3.1	256; 239; 193; 178; 161; 143; 91	AS
Norcoclaurine	$C_{16}H_{17}NO_3$	272	1.4	272; 255; 161; 123; 107	AS
Coclaurine	$C_{17}H_{19}NO_3$	286	2.8	286; 269; 237; 209; 175; 145; 107	AS
Isococlaurine	$C_{17}H_{19}NO_3$	286	3.6	286; 269; 237; 209; 107	inferred
N-Methylnorcoclaurine	$C_{17}H_{19}NO_3$	286	1.5	286; 255; 161; 123; 107	inferred
N-Methylcoclaurine	$C_{18}H_{21}NO_3$	300	2.8	300; 269; 237; 209; 175; 145; 137; 107	AS
Norarmepavine	$C_{18}H_{21}NO_3$	300	3.5	300; 283; 268; 189; 107	[5]
N-Methylisococlaurine	$C_{18}H_{21}NO_3$	300	2.6	300; 269; 237; 192; 175; 145; 107	[6]
4'-*O*-Methylcoclaurine	$C_{18}H_{21}NO_3$	300	3.9	300; 175; 143; 121	[7]
Armepavine	$C_{19}H_{23}NO_3$	314	3.4	314; 283; 252; 206; 190; 151; 145; 107	AS
3'-Hydroxy-*N*-methylcoclaurine	$C_{18}H_{21}NO_4$	316	2.2	316; 285; 192; 175; 143; 123	AS
4'-*O*-Methylarmepavine	$C_{20}H_{25}NO_3$	328	4.5	328; 297; 206; 121	[8]
Reticuline	$C_{19}H_{23}NO_4$	330	3.4	330; 299; 192; 175; 137	AS
Laudanine	$C_{20}H_{25}NO_4$	344	3.8	344; 206; 189; 174; 137	[6]
Tetrahydropapaverine	$C_{20}H_{25}NO_4$	344	4.6	344; 327; 192; 175; 151	AS
Laudanosine	$C_{21}H_{27}NO_4$	358	4.7	358; 227; 206; 189; 151	AS
**Proaporphines**					
N-Methylcrotsparine	$C_{18}H_{19}NO_3$	298	1.1	298; 255; 223; 209; 195, 167	inferred
N-Methylcrotonosine/Stepharine	$C_{18}H_{19}NO_3$	298	4.8	298; 267; 252; 237; 223; 208	inferred
**Aporphines**					
Anonaine	$C_{17}H_{15}NO_2$	266	4.6	266; 251; 249; 236; 220	[9]
Caaverine/Asimilobine	$C_{17}H_{17}NO_2$	268	3.6	268; 251; 236; 208	inferred
Roemerine	$C_{18}H_{17}NO_2$	280	4.6	280; 249; 219	[10]
Lirinidine	$C_{19}H_{21}NO_2$	282	3.7	282; 251; 219	inferred
N-Nornuciferine	$C_{18}H_{19}NO_2$	282	4.0	282; 251; 219	[10]
O-Nornuciferine	$C_{18}H_{19}NO_2$	282	4.6	282; 265	[10]
**Bisbenzylisoquinolines**					
Nelumboferine	$C_{36}H_{40}N_2O_6$	597	3.0	597; 566; 475; 192	[11]

Supplementary Table 9: Chromatographic and mass spectral data for compounds detected in sacred lotus plants fed deuterium-labeled amino acids. Expected *m/z* $[M+H]^+$ for BIAs resulting from all possible combinations of deuterated and non-deuterated dopamine and 4-HPAA/PAA are shown. L-Tyrosine-d4, L-phenylalanine-d5, and non-deuterated compounds were identified by comparison of retention times (Rt) and CID fragmentation with corresponding authentic standards (AS) or based on previously reported data. Deuterated alkaloids were identified by comparison of Rt with their corresponding non-deuterated BIAs, owing to the lack of authentic standards (NS).

Compound	*m/z* $[M+H]^+$	Rt (min)	Reference CID
**Primary Precursors**			
L-Tyrosine	182.08172	0.4	AS
L-Tyrosine-d4	186.10683	0.4	AS
L-Phenylalanine	166.08680	0.6	AS
L-Phenylalanine-d5	171.11818	0.6	AS
**1-Benzylisoquinolines**			
Norcoclaurine	272.12867	3.3	AS
Norcoclaurine-d2	274.14123	3.3	NS
Norcoclaurine-d4	276.15378	3.3	NS
Norcoclaurine-d6	278.16633	3.3	NS
Coclaurine	286.14432	4.7	AS
Coclaurine-d2	288.15688	4.7	NS
Coclaurine-d4	290.16943	4.7	NS
Coclaurine-d6	292.18198	4.7	NS
N-Methylcoclaurine	300.15997	4.8	AS
N-Methylcoclaurine-d2	302.17253	4.8	NS
N-Methylcoclaurine-d4	304.18508	4.8	NS
N-Methylcoclaurine-d6	306.19763	4.8	NS
**Proaporphines**			
Pronuciferine	312.15997	5.5	[11]
Pronuciferine-d1	313.16626	5.5	NS
Pronuciferine-d4	316.18508	5.5	NS
Pronuciferine-d5	317.19136	5.5	NS
**Aporphines**			
Nuciferine	296.16505	8.4	[11]
Nuciferine-d1	297.17134	8.4	NS
Nuciferine-d3	299.18389	8.4	NS
Nuciferine-d4	300.19016	8.4	NS

Supplementary Table 10: Kinetic parameters for recombinant NnOMT7. Assays were performed at a fixed saturating concentration of S-adenosylmethionine. Values represent the mean ± standard deviation of three independent measurements.

Substrate	K_m (μM)	V_{max} (nmol min^{-1} mg^{-1} protein)	k_{cat} (s^{-1})	k_{cat}/K_m (M^{-1} s^{-1})
(*R,S*)-Norcoclaurine	143 ± 30	12 ± 1	0.0076	53
(*S*)-*N*-Methylcoclaurine	48 ± 4	34 ± 1	0.0215	451

Supplementary Table 11: Engineered yeast (*Saccharomyces cerevisiae*) strains used to assay the function of NnCYP candidates.

Strain	Genotype
CEN.PK	*MATα; ura3-52; trp1-289; leu2-3,112; his3Δ1; MAL2-8C; SUC2*
YNO-0	CEN.PK *XII-4::* P_{TDH3}-*PsBUP1-myc*-T_{CYC1} P_{PGK1}-*PsCPR2-myc*-T_{ADH1}

Supplementary Table 12: Amino acid sequences of sacred lotus candidate enzymes and functionally characterized homologs involved in BIA biosynthesis in the Ranunculales. For each enzyme, the reference or GenBank accession code is provided. Sequences from sacred lotus are highlighted.

Specie	Name	Amino acid sequence
**NCS**		
Chelidonium majus	CmNCS1 ([12])	MIEGGYLDMGCTFYMDRIHVVKKGPNSCVIASAIIYEVKEEFVDVVVPLITTE<br>PLASMAEVISNYVLKKQRRVRKELTYEMEVPTSADSIWAVYSSHDIPRLLKE<br>VLLPGVFEKLDVIEGDGGVGTVLDIAFPPGAVPRTYKEKFVKINHEKRLKEV<br>VMIEGGYLDMGCTFYMDRIHVLEKSPNSCVIESSIIYEVKEEFADVVGPLITT<br>EPLASMSEVISNYVLKKQIRMFGYVIKPKLGLSLLLCFILCLVLLGVLLIGGVP<br>L
Coptis japonica	CjPR10A (BAF45338)	MRMEVVLVVFLMFIGTINCERLIFNGRPLLHRVTKEETVMLYHELEVAASAD<br>EVWSVEGSPELGLHLPDLLPAGIFAKFEITGDGGEGSILDMTFPPGQFPHH<br>YREKFVFFDHKNRYKLVEQIDGDFFDLGVTYYMDTIRVVATGPDSCVIKSTT<br>EYHVKPEFAKIVKPLIDTVPLAIMSEAIAKVVLENKHKSSE
Corydalis chelanthifolia	CcNCS1 ([12])	MYYFLEFFEKLDVIEGNGGVGTVLDIAFPPGAVPRSYKEKFVKVDHKNRLK<br>EVVMIEGGYLDLGCTFYMDRIHVLPKGANSCVIKSTLIYEIPDELVDSVGSL<br>MSTEPLASMAKVISDYVLKQRKMTANKILRKELKTEMEVATSADSIWAVYG<br>SPDIPRLLRDVLLPGVFEKLDVIEGNGGVGTVLDIAFPPGAVPRTYKEKFVK<br>VDHKNRLKEVVMIEGGYLDLGCTFYMDRIHVLPKGPNTCVIKSTLIYEVPDE<br>FADAVGSLISVEPLASMAEVISGYVLKQKKEAKKILRKELTHELEVPTSADSI<br>WAVYGSPDIPRLLRDVLLPGVFEKLDIVEGNGGVGTVLDIAFPPGAVPRSY<br>KEKFVKVDHDKHLKEVVMIEGGYLDLGCTFYMDRIHVLPKGPNSCVIESSLI<br>YEVREELADVVGSLISIEPLASMAEVISSYVLKQQLRVFGVVVQPRVGLSLL<br>LCLILCLVILGGLLIGGVSI
Nandina domestica	NdNCS3 ([12])	MRSGIVFLVLFFLGCEISQGRQLLESRLFRKSTIRKVLHHELPVAASAQEVW<br>DVYSSPELPKHLPEILPGAFKKVVVTGDGGVGTVIEMVFPPGVVPHRYKEK<br>FVLIDDEKFLKKVEMIEGGYLDMGCTFYMDTIQIVPTGPDSCIIKSSTEYYVK<br>PEFADKVVPLISTVPLQAMAEAIAKIVLEFKAKHKGFIEI
Nelumbo nucifera	NnNCS1 (ANI26411)	MMIGRVVNEMEVGVPADDIWAVYSSPELPRLFVQLMPNVYKKIDILQGDGT<br>VGTVLHIELADGIPEPRTWKEKFIKIDHQHREKVVRQIEGGFLDMGFRVFDV<br>IFKIIEKDACSCIIRSTTAFELDEKFENNANLITAGNLWGAAKAISNYVIQNKS<br>KRRNH
	NnNCS3 (ANI26412)	MRGQVTNELDVDVPVDDIWAVYGTPVLPTHIVQLQPDVFQKVDFIHGNGG<br>VGTILYVQLVPGAPEPRTWKEKFIKIDDEERLKVIRMIEGGYLDLGFTLFEYN<br>TQIIEKDAESCTIRSTTVFEVDEKFEANAALINATSAYGLAKAVANYVIQKKA<br>KACDV
	NnNCS4 (ANI26413)	MHAGQLSHELEVAVPASEVWEIYGTLKLGKACEELLPDVIHKAEVVEGDGG<br>VGTVLKVTLPPGLISYKEKFTKIDNEKRLKEVEVVEGGALDLGFRLYRIRLEII<br>EKTEVSSLIKSTVEYEIDDESANNASFATTKPLEQIAMAMGKYLTELKTE
	NnNCS5 (AND61511)	MIHSVSTELEVDLPADDIWAVYSSPELPKLVVKLMPHVYDKIDIVEGDGGVG<br>TVLQIVLTPEMMEPRTWKEKFVEINDGRRKKVVRQIEGGYLDMGFHFYEDI<br>FKIKKKSDSSCIIKSKSVFRVDHKHKANASLVTPDASAEMAKAVAEYAKQKK<br>ANSSSSSKDKAKACYE
	NnNCS7 (AND61512)	MMTARVTNEMEVGVPADDVWAVYGSPDLPKLFVQLMPQVYKRNDVLEGD<br>GTVGTVILIELDDALPEPRIWKEKFIKIDHQEREKLVRVIEGGFLDIGFRSFDII<br>FKVIEKDASSCIIQSTTAFELDDKFEDNANRITAGTLWWVAKAISNYVIQNKS<br>KSKSDNN
Papaver somniferum	PsNCS1 (AAX56303)	MSKLITTEPLKSMAEVISNYAMKQQSVSERNIPKKQSLLRKEITYETEVQTS<br>ADSIWNVYSSPDIPRLLRDVLLPGVFEKLDVIAGNGGVGTVLDIAFPLGAVP<br>RRYKEKFVKINHEKRLKEVVMIEGGYLDMGCTFYMDRIHIFEKTPNSCVIES<br>SIIYEVKEEYAGKMAKLITTEPLESMAEVISGYVLKKRLQVFGFEIKPKLRFNL<br>LLCLIICLVIAGGMFVAGVPL
	PsNCS2 (AAX56304)	MSKLITTEPLKSMAEVISNYVIQRESFSARNILNKNSLVKKEIRYDLEVPTSA<br>DSIWSVYSCPDIPRLLRDVLLPGVFQKLDVIEGNGGVGTVLDIVFPPGAVPR

		SYKEKFVNINHEKRLKEVIMIEGGYLDMGCTFYMDRIHIFEKTPNSCVIESSII<br>YEVKEEYAGKMAKLITTEPLESMAEVISGYVLKKRLQVFGFEIKPKLRFNLLL<br>CLIICLVIAGGMFVAGVPL
	PsNCS3<br>(XP_026441272)	MRKVIKYDMEVAVSADSVWAVYSSPDIPRLLRDVLLPGVFEKLDVIEGNGG<br>VGTVLDIVFPPGAVPRSYKEKFVNIDREKRLKEVIMIEGGYLDMGCTFYLDR<br>IHVVEKTKSSCVIESSIVYDVKEECADAMSKLITTEPLKSMAEVISNYVIQKES<br>FSARNILSKQSVVKKEIRYDLEVPISADSIWSVYSCPDIPRLLRDVLLPGVFE<br>KLDVIEGDGGVGTVLDIVFPPGAVPRSYKEKFVNIDREKRLKEVIMIEGGYL<br>DMGCTFYLDRIHVVEKSLSSCVIESSIVYEVKEEYADAMSKLITTEPLKSMA<br>EVISNYVIQRESFSARNILNKNSLVKKEIRYDLEVPTSADSIWSVYSCPDIPR<br>LLRDVLLPGVFQKLDVIEGNGGVGTVLDIVFPPGAVPRSYKEKFVNINHEKR<br>LKEVIMIEGGYLDMGCTSYLDRIHVVEKTSKSCIIKSSVVYEVKQECVEAMS<br>KLITTEPLKSMAEVISNYAMKQQSVSERNIPKKQSLLRKEITYETEVQTSAD<br>SIWNVYSSPDIPRLLRDVLLPGVFEKLDVIAGNGGVGTVLDIAFPLGAVPRR<br>YKEKFVKINHEKRLKEVVMIEGGYLDMGCTFYMDRIHVFEKTPNSCVIESSII<br>YEVKEEYAGKMAKLITTEPLESMAEVISGYVLKKRLQVFGFEIKPKLRFNLLL<br>CLIICLVIAGGMFVAGVPL
Sanguinaria canadensis	ScNCS1 ([12])	MRKELTHEMEVPASADAIWAVYSSHDIPRLLKEVLLPGVFEKLDVIAGDGG<br>VGTVLDIAFPPGAVPRRYKEKFVKINHEKRLKEVEMIEGGYLDMGCTFYMD<br>RIHVVEKGPNSCVIESAIIYVVKDECADVVVPLITTEPLASMAEVISNYVLRK<br>QIRLFGYVIKPKLGLSILLSLILCLVILGVLLIGGVPF
Stylophorum diphyllum	SdNCS1 ([12])	MRKEVRYEMEVPTSADSIWAVYSSHDIPRLLKEVLLPGVFEKLDVIEGDGG<br>VGTVLDIAFPPGAVPRTYKEKFVTINHEKRLKEVIMIEGGYLDMGCTFYMDR<br>IHVLEKGPKSCIIASAIIYEVKEEFADVVVPLITTEPLASMAEVISNYVLKKQRR<br>VRKELTYEMEVPTSADSIWAVYSSHDIPRLLKEVLLPGVFEKLDVIEGDGGV<br>GTV
Thalictrum flavum	TfNCS<br>(ACO90248)	MMKMEVVFVFLMLLGTINCQKLILTGRPFLHHQGIINQVSTVTKVIHHELEVA<br>ASADDIWTVYSWPGLAKHLPDLLPGAFEKLEIIGDGGVGTILDMTFVPGEFP<br>HEYKEKFILVDNEHRLKKVQMIEGGYLDLGVTYYMDTIHVVPTGKDSCVIKS<br>STEYHVKPEFVKIVEPLITTGPLAAMADAISKLVLEHKSKSNSDEIEAAIITV
	TfNCS2 ([12])	MKMEVVFVFFMILGTINCQKLILTGRPFLNRQGIINQVSTVTKGVHHELEVA<br>ASADDIWSVYSWPGLAKHLPDLLPGAFEKLEIIGDGGVGTILDMTFTPGEFP<br>HEYKEKFILVDNEHRLKKVQMIEGGYLDLGVTYYMDTIQVIPTGTNSCVIKS<br>STEYHVKPEFVKIVEPLITTGPLAAMAEAISKLVLEHKYKSNSDEIDASKNNL<br>KMVINM
Xanthorhiza simplicissima	XsNCS1 ([12])	MRMEVVLVVFLLFIGTVNCERMIFSGRPLLHRVTNEETVILYHELEVPASVD<br>ELWSVEGSPELGKNLPDLLPGIFADFKITGDGGEGSILDMTFPPGQFPHHY<br>REKFVFFDHKNHYKLVQMIDGDFFDLGVTYYMDTIRVVATGPDSCVIKSST<br>EYHVKVEFAKIVKPLIDTVPLAIMSEAIAKVVLEKKYKRSE
**OMT**		
Coptis chinensis	Cc6OMT1<br>(AXC09385)	MQIQNEEQEQDMKSHAQILNHMCGIVDSVVLKCAVELNLFDVISNNKDSKP<br>IALSSLATSPTLVSIKPNNLYRLLRYLVHMNLLTIHVEGNDETFSLTELSKLLL<br>RDQNRSLVDWALAIIDETVIDGWHELSGCCTSPTGPTPFERVHGKSVWEL<br>AGENAGMNQVINDAMVSDTILVMPVFVQCCDKLLNGITSMVDIGGGVGMT<br>MSYIVKAFPHIKCTVFDLPHVIASSAQLPGVEMVGGDMFKFIPPADAIFLKF<br>MLHNWHDKECITILKKCKEVIPQDKGKVIILDIVTDQNEQDDDLTRAKMNLDI<br>DMMVTSGGRERTENEWEVLLKLAGFSRHEIIPIMAVQSVIVAYP
	Cc6OMT2<br>(AXC09386)	MVKKDNLSSQAKLWNFIYGFAESLVLKCAVQLDLANIIHNNGTSMTLSELSS<br>RLPSQPVNEDALYRVMRYLVHMKLFTKASIDGELRYGLAPPAKFLVKGWD<br>KCMVGSILAITDKDFMAPWHYLKDGLAGESGTAFEKALGMNIWGYMAEHP<br>EKNQLFNEAMANDSRLIMSALVKECGNIFNGITTLVDVGGGTGTAVRNIAN<br>AFPHIKCTVYDLPHVIADSPGYSEVHCVAGDMFKFIPKADAIMMKCILHDWD<br>DKECIEILKRCKEAVPIEGGKVIIVDIVLNVQSEHPYTKMRLTLDLDMMLNTG<br>GKERTEEEWKNLIHDAGYKGHKITQITAVQSVIEAYPY
Coptis japonica	Cj6OMT<br>(BAB08004)	MEVKKDNLSSQAKLWNFIYGFAESLVLKCAVQLDLANIIHNSGTSMTLSELS<br>SRLPSQPVNEDALYRVMRYLVHMKLFTKASIDGELRYGLAPPAKYLVKGW<br>DKCMVGSILAITDKDFMAPWHYLKDGLSGESGTAFEKALGTNIWGYMAEH<br>PEKNQLFNEAMANDSRLIMSALVKECGNIFNGITTLVDVGGGTGTAVRNIA<br>NAFPHIKCTVYDLPHVIADSPGYSEVHCVAGDMFKFIPKADAIMMKCILHDW

		DDKECIEILKRCKEAVPVKGGKVIIVDIVLNVQSEHPYTKMRLTLDLDMMLNT<br>GGKERTEEEWKKLIHDAGYKGHKITQITAVQSVIEAYPY
	Cj4'OMT<br>(BAB08005)	MAFHGKDDVLDIKAQAHVWKIIYGFADSLVLRCAVELGIVDIIDNNNQPMAL<br>ADLASKLPVSDVNCDNLYRILRYLVKMEILRVEKSDDGQKKYALEPIATLLS<br>RNAKRSMVPMILGMTQKDFMTPWHSMKDGLSDNGTAFEKAMGMTIWEYL<br>EGHPDQSQLFNEGMAGETRLLTSSLISGSRDMFQGIDSLVDVGGGNGTTV<br>KAISDAFPHIKCTLFDLPHVIANSYDLPNIERIGGDMFKSVPSAQAIILKLILHD<br>WNDEDSIKILKQCRNAVPKDGGKVIIVDVALDEESDHELSSTRLILDIDMLVN<br>TGGKERTKEVWEKIVKSAGFSGCKIRHIAAIQSVIEVFP
	CjSOMT<br>(BAA06192)	MCTSLSELKCPVFSTKRKLLLEFALRTSVDMAAQEGVNYLSGLGLSRLICLP<br>MALRAAIELNVFEIISQAGPDAQLSPSDIVAKIPTKNPSAAISLDRILRMLGAS<br>SILSVSTTKSGRVYGLNEESRCLVASEDKVSVVPMLLFTSDKAVVESFYNIK<br>DVVLEEGVIPFDRTHGMDFFQYAGKEERVNKSFNQAMGAGSTIAFDEVFK<br>VYKGFDNLKELVDVGGGIGTSLSNIVAKHPHIRGINFELPHVIGDAPDYPGV<br>EHVPGDMFEGVPNAQNILLKWVLHDWDDDRSIKILKNCWKALPENGTVIVI<br>EFVLPQVLGNNAESFNALTPDLLMMALNPGGKERTTIEFDGLAKAAGFAET<br>KFFPISQGLHVMEFHKINC
	CjCoOMT<br>(BAC22084)	MDTPNTFQNDDEIKAQAQVWKHMFGFAETIMLRSTVSLGIPDIIHNNGPVTL<br>SQLVTHLPLKSTSIDRFHHFMRYLVHMQLFTISTDQITKEDKYELTPASKLLV<br>HGHQKSLAPYVMLQTHPEEFSVWSHVINVLDGKKPYWESNDTSMYEKTE<br>GDPEINEILNDAMTSHSTFMLPALVSGLMKENVLDGVASIVDVGGNSGVVA<br>KGIVDAFPHVKCSVMDLNHVIERVIKNPKLDYVAGDMFTSIPNADAILLKSTL<br>HNYEDDDCIKILNIAKEALPSTGGKVILVEIVVDTENLPLFTSARLSMGMDM<br>MLMSGKERTKKEWEDLLRKANFTSHQVIPIMAIESIIVAYS
Coptis teeta	Ct7OMT<br>(AXC09387)	METILQGQQNITKLLFAFADTMALKCVVELRIADIINSHGLPISLSEIAAGIQST<br>SSSSSPPNINYLFRIMRLLVRKGVFSSHAPNQNEETLYGLTNSSKWLLRDA<br>NFSMTPIIQALTHHCSMDSFHKLNKCVEEGGYAFAKANGCEIWEFASMNPE<br>FNRLFNSAMASTSKIAVDAILSGYKNGFDGLRSLVDVGGGTGTLIGEIVKAY<br>PHLTGTNFDLPHVVATAPEHTGVVHVGGDMFVEIPHADAIILKWILHDWND<br>EDCVKILKNCHKAIANRGVKVIIVEIVLQPDGVAPLDETRLIFDLSMIAHSSGG<br>KERTETEWEKLLRDGGFSRHRIIQIPDVTSIIEAYP
	CtSOMT<br>(AXC09384)	MAAQEGVNYLSGLGLSRLICLPMALRAAIELNVFEIISKAGPDAQLSPSDIVA<br>KIATKNPSAAISLDRILRMLGASSILSVSTTKSGRVYGLNEESRCLVASEDKV<br>SVVPMLLFTSDKAVVESFYNIKDVVLEEGVIPFDRTHGMDFFQYAGKEQRV<br>NKSFNQAMGAGSTIAFDEVFEVYKGFNNLKELVDVGGGIGTSLSNIVAKYP<br>HIRGINFELPHVIGDAPDYPGVEHVPGDMFEGVPNAQNILLKWVLHDWDD<br>DRSIKILKNCWKALPENGTVIVIEFVLPQVLGNNAESFNALTPDLLMMALNP<br>GGKERTTIEFDGLAKAAGFAETKFFPISQGLHVMEFHKINC
Eschscholzia californica	Ec7OMT<br>(BAE79723)	MDEEIILGQADICKYMYGFVDSMTLRCVVELGIPDIIHSHGRPITLTEILNGIP<br>NLSSSFDINYLQGIMTILVRRRVFAVHKFDPKDGTNLTEIRYGLTPSSKCLLK<br>DSKFNLAPFVLLETHPWITDPWNYLGKCVQEGGSGFVKAHGSDVFKFGSD<br>HPEFFKLFYDGMECSTKVLVQVVLDKYQQVFKDVKSIVDVGGGTGMMISEI<br>VKNHPHIKGINFDLPHVVAEAPDYPGVEHVGGDMFVEIPQADAITMKGILHD<br>WNDDACVKILENCKKAIPKNGKVIIIDCVLNPDGDDLFDDIKVVSDLGMRVH<br>CSDGKERTEAEWEKLLKKGGFPRYKITHVVTVQSMIEAYPE
	Ec4'OMT<br>(BAM37633)	MGLEFNEEVDIKAQAHLWNIIYGFADSLVLRSAVELGIADIIKNNNGSITVSEL<br>ASKLPISNVNSDNLYRVLRYLVHMGILKETKSTINGGEIKKLYSLEPVGSLLV<br>KDAERNMVPIVLGMTQQDFMIPWHYIKEGLGEGSTAFEKGMGMTLWEYLE<br>GHPEQGHLFNVGMEGETRLLTKTLIESCKDTFEGLSSLVDVGGGNGTTIKA<br>ISEAFPHIKCSLYDLPHVVADSHDLPNIEKIPGDIFKFIPNAQAILLKLILHDWS<br>DEDSVKILKKCREAVPQDTGRVIIVDVALEEESEHPLTKTRLVLDVDMLVNT<br>GGRERSEDDWAKLLKLAGFRTHKIRHIAAVQSVIEAFP
	EcSOMT<br>(BBA20643)	MEKGKLEVGEEMELQGQADICKLMLAFIDSMALKCAVELGIPDIIHSQGQPI<br>TLSEIINGIPNLSPSFDINYLFRIMRLLVRNRVFSAYEPDLKDGSSGTKTLYGL<br>TPSSKWLVKDSKISLAPLVLAENHPWLLDPWHYLGKCVQEGGFAFAKAHG<br>SEIWKFGSENPEFNKLFSVGMACSSTLVVDAILDDYHEGFGDLESIVDVGG<br>AIGTLINEIVKKYPHIRGTNFDLPHVVAEALENPGVAHVGGDMFVEIPSADA<br>VILKWVLHDWNDEDCVKILKNCNKAISNKGKLIIIECVLKPDGEGLFDGLGLA<br>FDLLMIAHSSGGRERTEAEWKKLLKAGGFSRYKITPIKGIPSIIEAYPDI

Glaucium flavum	GfOMT2<br>(AKO60153)	MEATKSDQANQANIWKLIYGFAESLVLKCAIQLEIADTIHNHGEPMSLSELA<br>SKLPVQPVNSDRLYRVMRYLVHMKLFNKEKTSINGEFKYSLAPPAKFLIKG<br>WEKSMVASILAINDKDFLAPWHHLKDGLSGDCDAFEKALGKSIWVYMSEN<br>PEKNQLFNEAMACDTRLVTSALVNDCQSVFKGINTLVDVGGGTGTAVKAIS<br>KAFPHIKCSIYDLPHVIADSPEIPNVVKIEGDMFKAIPSADAILMKCILHDWND<br>DECIQILKKCKEAVPQEGGKVIIVDVVLNMDLTHPYSKIRLTLDLDMMLNTG<br>GKERTVEEWKKLIDAAGFASFKITEISAVQSVIEAFPY
	GfOMT1<br>(AKO60152)	MGVSDNKPESQEVDIKAQAHLWNIIYGFADSLVLRCAVEIGIADIIKSNNGSI<br>SVTELASKLPITNVNSDNLYRVLRYLVHMGILKEVSDSNEVKLYSLQPVATL<br>LLRDAERSMVPIILGMTQKDFMIPWHFMKEGLGNDTTAFEKGMGMTIWQY<br>LEGHPEQSNLFNEGMAGETRLLTKSLIDGCRDTFEGLTSLCDVGGGNGTTI<br>KGIYDAFPQIKCSVYDLPHVIASSPEHPNIERIPGDMFKSVPSAQAILLKLILH<br>DWTDEECVNILIKCREAVPKDTGKVIIVDVALEEESQHELTKTRLILDIDMLV<br>NTGGRERSEDDWEKLLKRAGFRGHKIRHIAAIQSVIEAFP
	GfOMT6<br>(AKO60157)	METKGEARMNSCYISEAGHLGRLICLPMALRAAVELNVFNIISEFGPGAQLS<br>SRDLVAKIPTTNPNAHVYLERILRLLAASSFLSVSTRTSSSPESITNTNGHHN<br>GDTNGVVNHDNEKVTERVYGLTKESHCLVPRKDDGVSLVPMLMFVADKIV<br>VESFYNLKEVVLQEGRVPFDMTHGASIFEYAGKDPRMNKVFNEAMGDFSV<br>IAFDEVLKVYNGFLDMKELVDVGGGIGTSLSNIVTKYPFIRGINFDLSHVISS<br>APNYTGVEHVAGDMFEELPKAQNILLKWVLHDWDDKQCLKLLKTCWNSLP<br>AEGGKVIVIEFVVPSKIADNPESYNALTPDLLMMALNPGGKERTLLEFYDLA<br>NAAGFAKAKPFPISEGLHVIEFHK
	GfOMT7<br>(AKO60158)	MGSTQDQFKPTPIEEEEEACMYAMQLASASVPVMVLKAAVELNVLEIIAKH<br>GPGAQISASAIAAHIPNIKNPNAPVMLDRMLRLLASYKILTCTVKDLDDQGLV<br>QQRLYGLALVCKFLVKNEDGCSMAPLLLMNQDKVFLESWYHLKDAVLDGG<br>IPFNKAYGMNAFEYQGADPRFNKIFNRGMSDHTTITMKKILETYKGFEGLTS<br>LVDVGGGIGVTVDMIVSKYPSIKGINFDLPHVIKDAPSYPGVEHVGGDMFAS<br>IPKGDAIFMKWILHDWSDEHGIKILKNCYEALPDHGKVILVECIIPPYPETSLA<br>GLGVFHVDNIMLAHNPGGKERTEKEFEALAKGTGFAGFRVICSAFNTWIME<br>FSKN
Nelumbo nucifera	NnOMT1<br>(AXJ91466)	MEIQKEGQAAAAKIWKFVYGFADCLVLRCAIDLGIADIIHKQGEPLTLSELGA<br>QIPVQPVNTDHLHRLMRYLVHMKIFTKETLDGEARYGLAPPAKFIVKGWDK<br>SIVSIILVVTDKDFMAPWHCLKDSLCGEGTAFEKALGRSIWTYMADHPEKN<br>KLFNEGMACDTKLLISALVQDCKDLFQGIMSLVDVGGGTGTAMRAIAKAFP<br>HLKCTIYDLPHVIADSPDYPEVDRIAGDMFKHIPSADAILLKCILHDWDDGEC<br>IEILKRCKESVPREGGKVIIVDIVVDLESKHPLTKTRLSLDLDMMVTTGGKER<br>TEAEWKKLLNAAGFPVFKITHISAVQSVIVAYPY
	NnOMT2<br>(AXJ91465)	MEIPKEVQADEVEIWKFGYDFADTLVLRCAIEFGIADIIHKQGEPLTLFELGA<br>QIPVQPVNTDHLHRLMRYMVHMKIFTKETLGGEEXYGLSPHGKFLVKGWD<br>KSMASAILAITDEDFFAPWHCLKDVLAGEGTAFEKALGKSIWAYVADHPEK<br>NKLFNEVMACDTSFITSVLIQDCKDVFQGIKSVVDVGGGTGTAMRDIAKAFP<br>HLKCTIYDLPHVIADSPDYPEVDRIAGDMFKHIPSADAILLKWILHDWDDGE<br>CIEILKRCKESVPREGGKVIIVDIVLDPESKDPLTKARLRLDLDMMVYTGGKE<br>RSEAEWKKLLNAAGFPGYKILHVAAVQSVIMAYPY
	NnOMT3<br>(AXJ91464)	MEIQKEVQAADVEIRKFGYGFADILVIRCAIQLGIADIIHKQGEPLTLSELEAQI<br>PVKPVNTDHLHRLMRYMVHMKIFTKETPDGEERYGLAPLGKFLVNGWDRN<br>MVSAILAVTDKDFMVPWYRLKDSLVGEGTAFEKALGKTICECMADHPEKKK<br>PFNEAMACDTTRLLTSALIQDCKDLFQGIMSLVDVGGGTGTAMRDIAKTFP<br>HLKCTIYDLPHVIADSPDYPEVDRIAGNMFKHIPSADGILLKCILHDLGDRQCI<br>EILQRCKESVPREGGKVIIVDIVLDPESTDPLTKARLRLDLDMMVYTGGKER<br>SEAEWKKLLNAAGFPRYKILHIAAVQSVIEAYPY
	NnOMT4<br>(AXJ91463)	MENQKEVQAAEAKIWNFVYGFADTLVLRCAIELGIADIIHKQGEPLTLSELG<br>AQIPLKSVNTDHLHRLMRYLVHMKLFTKETLDGEARYGLAPPAKLLVKWW<br>EDKGLASIIFGITDKDFIAPWHHLKDSLAGDGEETTFEKVLGKSISTYMADHL<br>EKSMLFNESMVHDTRLFTSVLIQDFKDVFQGIKSLVDVGGGSGTDMGAIAK<br>AFPHLKCTIYGLPHVIADSPDYPEVDRISGDMFKHIPSADAILLKCILHYWGD<br>GQCIEILKRCKESVPREGGIVIIADAVVDLESKHPYLTKTLLSTDLDMMLNTG<br>GKERTEAEWKKLFNAAGFPAYKITHVADVEYSVIEAYPY

	NnOMT5 (XP_010276063)	MEEDMKAQAQLWKHIYGFVESFTLKCAIELGIADILYEHGQPMTLSELASSI PLPSVSQDGLYRVLRYLVHMKLFDLQVDSDGLKKYRLTPASKLLVKNQEKN LASFVLLQLYEIDTWNHLSAAVEGTVTPWEKCHGGVDYIEYCKKDSVANQL LSDAMTSHTSMVTDALVKGCKKAHILDGVGSLIDVGGCTGVAARAIAKWFP SIKCAVFDLPHVVANAPECPEVTRIGGDMFVSIPKTDVVFMKSVLHDWGDE DCVKILKKCKEAISEKGGKVVIVDIVMDMESSPNEFTGARLGMEMDMLVAV GGKERSEKEWHKLFKEAGYSGYKITPIVAIESIIEVFP
	NnOMT6 (XP_010274375)	MEYKQELQNPLMDQAMEKAKEEKMEELRAQVRIRNYMNGYADSMALKCA VENGIADIIQKHAKPIALSELAKALPLPTVSTEHLERLMKYWVQLGLFTHEKD GSHGLTRCSKYLLREENTTMAAIILGLVTEWTIGPWHCLARSLEGGPTAFR RYHGRDMWDYVAGHPEASRLFNESMAGDTRLLLPVLMQECGSWLFEGIS SLVDVGGGNGTAMVEIAKKFPGIKCTVFDLPHVIRNTNSSESTGVEWVEGD MFESIPPADAVLLREAIPKERGKVIIIDIVMDIEQDPELIRAKLMTDIDMMSDD LTVMDQRHHEAQALSRLAVKAPNPLLRRWHYDRNHQRLHEQSQPPQLAL SDYLDCDNVCS
	NnOMT7 (XP_019055563)	MEDMKAQAQVWKHIYGFAESLALKCAIELGIADILYEHGQPMTLSELASSIP LPSVSQDGLYRVLRYLVHMKLFDLQVDSDGLKKYWLTPASKLLVKSQEKN LASFALLIFYEMDAWHHLSAAVEGTVTPFEKCHEGEDLEELFGKDSVINRLL SEGMTNLTSLMADALVKGCKKAHILDGVGSLIDVGGSTGVAARAIAKSFPSI KCAVLDLPHVVANAPECSEVTWIEGDMFVSIPKTDVVFMKVRSVLHDCGD EDTVKILKICKEAISEKGGKVVIVEIVMDVESSSSPNEITGAKLNLDMSMLVT PGGKERSEEDWQKLFKEAGYSRYKITPIAAFESIIEVFP
	NnOMT8 (XP_010272384)	MGQGDEMELEAQAQIWKYIFSFVDSFTLRSAVELGIPDIIHSQGRPITLSQL SACLPIDYANPDRLNRLMRYLVSIGIFSREHGSADDEDDQEDKFGLTCLSKL LVRKLENNMVPFSMLDFKVLMEPWYHLTWSLDGRASGVTAFERVYGTKF WDYAGQDLEFGEKLNEAMACDTSSTMPTLLQQFNQVFADMSSVVDVGGG TGTAAMAIAKSFPNVKCTVFDHPHVVVDDQSDSGGVAKVSGDMFNFIPRA DNLLLKWILHNYGDEDCLVILKRCKEALPERGGGKVVIVETVMEGDHNDET AAEIHDDEFTPCRLIMDMEMMLLFGGKERTAKEWQSLVERAGFRRCTMER INSSMHSLIQAYP
Papaver somniferum	Ps6OMT (AAQ01669)	METVSKIDQQNQAKIWKQIYGFAESLVLKCAVQLEIAETLHNNVKPMSLSEL ASKLPVAQPVNEDRLFRIMRYLVHMELFKIDATTQKYSLAPPAKYLLRGWE KSMVDSILCINDKDFLAPWHHLGDGLTGNCDAFEKALGKSIWVYMSVNPE KNQLFNAAMACDTRLVTSALANECKSIFSDGISTLVDVGGGTGTAVKAISKA FPDIKCTIYDLPHVIADSXEIPNITKISGDMFKSIPSADAIFMKCILHDWNDDE CIQILKRCKEALPKGGKVIIVDVVIDMDSTHPYAKIRLTLDLDMMLNTGGKER TKEEWKTLFDAAGFASHKVTQISAVQSVIEAYPY
	Ps7OMT (AAQ01668)	MDTAEERLKGQAEIWEHMFAFVDSMALKCAVELGIPDIINSHGRPVTISEIV DSLKTNTPSSSPNIDYLTRIMRLLVHKRLFTSELHQESNQLLYNLTRSSKWL LKDSKFNLSPLVLWETNPILLKPWQYLGKCAQEKSSPFERAHGCEIWDLAL ADPKFNNFLNGAMQCSTTTIINEMLLEYKDGFSGIAGSLVDVGGGTGSIIAEI VKAHPHIQGINFDLPHVVATAAEFPGVKHVGGDMFVDIPEADAVIMKWILHD WSDEDCTIILKNCYRAIRKKKNGKVIIVDCVLRPDGNDLFDKMGLIFDVLMM AHTTAGKERTEAEWKILLNNAGFPRYNVIRTPAFPCIIEAFPE
	PsN7OMT (XP_026431254)	MEVVSQIDQENQAIIWKQIYGFSESLLLKCAVQCEIAETIHNHGTPMSILELA AKLPIDQPVNIDRLYRVMRYLVHQKLFNKEVISTLNGGTVQVTEKYWLAPP AKYLIRGSQQSMVPSVLGIIDEDMFAPWHILKDSLTGECNIFETALGKSISVY MSENPEMNQISNGAMAFDSGLVTSHLVNECKSVFGDEIKTLVDVGGGTGT ALRAISKAFPNIKCTLFDLPHVIADSPEIPTITKVSGDMFKSIPSADAIFMKNIL HDWNDDECIQILKRCKDVVSAGGKLIMVEMVLDEDSFHPYSKLRLTSDIDM MVNNGGKERTEKEWEKLFDAAGFASCKFTQMSVGFAAQSIIEVY
	Ps4'OMT2 (AAP45314)	MGSLDAKPAAATQEVSIKDQAQLWNIIYGFADSLVLRCAVEIGIADIIKNNDG AITLAQLAAKLPITNVSSDYLYRMVRYLVHLNIIEQETCNGGVEKVYSLKPVG TLLLRDAERSMVPMILGMTQKDFMVSWHFMKEGLGNGSTTAFEKGMGMD IWKYLEGNPDQSQLFNEGMAGETRLLTKTLIEDCRDTFQGLDSLVDIGGGN GTTIKAIYEAFPHIKCTLYDLPHVVANSHDLPNIEKVPGDMFKSVPSAQAILL KLILHDWTDEECVNILKKCKEAIPKETGKVIIVDVALEEESNHELTKTRLILDID MLVNTGGRERTADDWENLLKRAGFRSHKIRPIRAIQSVIEAFP

	PsOMT2<br>(AFB74612)	MEIHLESQEQEMKYQSQIWNQICGTVDTSVLRCAIQLGIFDAIHNSGKPMIT<br>LTELSSIVSSPSSSSIEPCNLYRLVRYLSQMDLISIGECLNEATVSLTGTSKLL<br>LRNQEKSLIDWVLAISCEMMVVVWHELSSSVSTPADEPPIFQKVHGKNALE<br>LAGEFPEWNDLINNAMTSDTSVTKPALIQGCGKILNGVTSLIDVGGGHGAT<br>MAYIVEAFPHIKGAVIDLPHVVEAAPERPGVEFISGDIFKSISNADAVLLKYVL<br>HNWEDTECVNLLKRCKEAVPADKGKVIIMDLVIDDDDNSILTQAKLSLDLTV<br>MNHGGGRERTKEDWRNLIEMSGFSRHEIIPISAMPSIIVAYP
	PsOMT3<br>(AFB74613)	MEVVSKIDQENQAKIWKQIFGFAESLVLKCAVQLEIAETLHNNVKPMSLSEL<br>ASKLPAQPVNEDRLYRILHFLVHMKLFNKDATTQKYSLAPPAKYLLKGWEK<br>SMVPSILSVTDKDFTAPWNHLGDGLTGNCNAFEKALGKGIRVYMRENPEK<br>DQLFNEGMACDTRLFASALVNECKSIFSDGINTLAGVGRGTGTAVKAISKA<br>FPDIKCTIHDLPEVTSKNSKIPRDVFKSVPSADAIFMKSILHEWNDEECIQILK<br>RCKEAIPKGGKVIIADVVIDMDSTHPYSKSRLAMDLAMMLHTGGKERTEED<br>WKKLIDAAGFASCKITKLSALQSVIEAYPH
	PsSOMT1<br>(AFK73709)	MATNGEIFNTYGHNRQTATVTKITASNESSNGVCYLSETANLGKLICIPMAL<br>RAAMELNVFQLISKFGTDAKVSASEIASKMPNAKNNPEAAMYLDRILRLLGA<br>SSILSVSTTKKSINRGGDDVVVHEKLYGLTNSSCCLVPRQEDGVSLVEELLF<br>TSDKVVVDSFFKLKCVVEEKDSVPFEVAHGAKIFEYAATEPRMNQVFNDG<br>MAVFSIVVFEAVFRFYDGFLDMKELLDVGGGIGTSVSKIVAKYPLIRGVNFD<br>LPHVISVAPQYPGVEHVAGDMFEEVPKGQNMLLKWVLHDWGDERCVKLL<br>KNCWNSLPVGGKVLIIEFVLPNELGNNAESFNALIPDLLLMALNPGGKERTI<br>SEYDDLGKAAGFIKTIPIPISNGLHVIEFHK
Thalictrum flavum	Tf6OMT<br>(AAU20765)	MEMINKENLSSQAKLWNFIYGFADSLVLKSAVQLDLANIIHNHGSPMTLSEL<br>SLHLPSQPVNQDALYRVLRYLVHMKLFTKSSIDGELRYGLAPPAKFLVKGW<br>DKCMLGAILTITDKDFMAPWHYLKEGILNDGSTSTAFEKALGTNIWDYMAE<br>HPEKNQLFNEGMANDTRLIMSALVKECSSMFDGITTIVDVGGGTGTAVRNI<br>AKAFPHIKCTVYDLPHVIADSPGYTEINSIQGDMFKYIPNADAIMMKCILHDW<br>DDKECIEILKRCKDAVPRDGGKVIIIDIILDVKSEHPYTKMRLTLDLDMMLNT<br>GGKERTEEEWKKLIHDAGYKGYKITHISAVQSVIEAYPY
**NMT**		
Coptis japonica	CjCNMT<br>(BAB71802)	MAVEAKQTKKAAIVELLKQLELGLVPYDDIKQLIRRELARRLQWGYKPTYEE<br>QIAEIQNLTHSLRQMKIATEVETLDSQLYEIPIEFLKIMNGSNLKGSCCYFKE<br>DSTTLDEAEIAMLDLYCERAQIQDGQSVLDLGCGQGALTLHVAQKYKNCR<br>VTAVTNSVSQKEYIEEESRRRNLLNVEVKLADITTHEMAETYDRILVIELFEH<br>MKNYELLLRKISEWISKDGLLFLEHICHKTFAYHYEPLDDDDWFTEYVFPAG<br>TMIIPSASFFLYFQDDVSVVNHWTLSGKHFSRTNEEWLKRLDANLDVIKPM<br>FETLMGNEEEAVKLINYWRGFCLSGMEMFGYNNGEEWMASHVLFKKK
Eschscholzia californica	EcTNMT<br>(ACO90222)	MGSSAGEIMGRLMKGEIEDEELKKLIRHQWDRRIEWGYKPTHEKQLAFNL<br>DFIKGLKEMVMSGEIDTMNKETYELPTAFLEAVFGKTVKQSCCYFKDENSTI<br>DEAEEAAHELYCERAQIKDGQTVLDIGCGQGGLVLYIAEKYKNCHVTGLTN<br>SKAQANYIEQQAEKLELTNVDVIFADVTKFDTDKTYDRILVVETIEHMKNIQL<br>FMKKLSTWMTEDSLLFVDHISHKTFNHNFEALDEDDWYSGFIFPKGCVTILS<br>SSTLLYFQDDVSALDHWVVNGMHMARSVEAWRKKLDETIEAAREILEPGL<br>GSKEAVNQVITHIRTFCIGGYEQFSYNNGEEWMITQILFKKK
Glaucium flavum	GfCNMT ([13])	MDLMATSKQVKKKEELLKNMELGLVPDEEIRRLIRIELEKRLKWGYKPTHQ<br>QQLAQLLDLVHSLKKMKIATEMESLDLKLYEAPFSFVQIKHGSTIKESSSYF<br>KDESMTLDEAEIAMLDLYVERAQIEDGQSVLDLGCGLGAVTLHVAKKYKNC<br>HVTGLTNSVEQKDFIEGKCKELNLSNVKVILADVTSHEMEDKFDRIFAVELIE<br>HMKNYELLLRRISKWMKDDGLLFIEHVCHKTFAYHYEPIDEDDWYTEYIFPA<br>GTLTLSSASLLLYFQDDVSVVNHWTLSGKHYSRSHEEWLKRIDGNMDAVK<br>EIMKSITKTEEEAVKLINFWRIFCMCGAELFGYKDGEEWMMSHVLFKKKQL<br>LQQC
	GfNMT6 ([13])	MGVEITESSSTTMANKKAEVGELLRKLEHGLLPYDEIRRLMRIELGRRLQW<br>GYKPTHEQQLSQVLKLARSLRTMNIATEIDTLDEQMYEVPIPFLQLMFGSTI<br>KGSCCYFKDDSTTLDEAEIAMLDLYCERAQIKDGQSVLDLGCGQGALTLHV<br>ANKYKNCRVTAVTNSVSQKEFIEEQSRRLNLKNVEVMLADITTHEMEDTYD<br>RILVIELFEHMKNYELLLRKISKWLSKDGFLFIEHICHKTFAYHYEPIDEDDW<br>YTEYIFPAGTMIIPSASFFLYFQDDLTVAKQWTLSGKHFARTKEEWLKRLNA

		NVDEVMKIMESFSGSKEGAVKWTNYWRGFCLSGMEMYGYNNGEEWMAC<br>HVLFKK
	GfNMT4 ([13])	MTMEANNAKKEAIENLWEQMMMGLVPDHEITRLMKSELQKRLNWGYKPT<br>HQQQISQLLDFAKSLRRMEMSLDFDNLELDTKMYETPESFQLIMSGTTLKE<br>SSGLFTDETATLDQTQIRMMDLYLEKAKIKDGQSILDLGCGHGALILHVAQK<br>YRNCNVTGVTNSIAQKEFIFKQCKKLGLSNVEMVLADVTKCEMKATFDHIF<br>VIGLIEHMKNFELFLRKVSEWMKSDGLLFMEHYCHKSFAYQWEPMDDDDL<br>FSKYVFPPGSAIIPSASFLLYFQDDLTVVDHWTLSGNHFARTHQEWLKRIDS<br>QSDEIKGIFESFYGISKEEAVKLINYWRVFCLFGVEMFGYNNGEEWMISHLL<br>FKKK
	GfNMT5 ([13])	MGSNETNGELKTKEMVPDLLKRLESGLVADEELRKLIRFELERRLKWGYKP<br>THEQQLAELLKLAHSTKQMKIATETDSLNSTMYEVPIPFLQLQFGSAIKESC<br>CYFKDESTTLDEAEVAMMDLYLERTQIKDGQSILDLGCGLGALAFHIVQKYP<br>NCNVLAITNSVEQKEFIEEKCKIRKVENVKVSLADICTLEMKTTFDRIFAIGLL<br>EHMKNYQLLLKKFSNWMKQDGLLFIEHLCHKTLAYHYEPLDEDDWYTEYF<br>FPAGTLTIISSSFLLYFQDDVSIVNHWSLSGKHFSRSNEEWLKRMDMKIDEV<br>KEILEAAFENKDHDITKLINHWRFLAINATEMFGYNNGEEWMVSQVLFKKK
	GfTNMT ([13])	MGSNEAQVKKESIGEIMGKLMQGEIGDEELSKRIKEIFGKRLQWGYKPTHQ<br>QQLAFNLDFIKSLKEMDMSGEIDTMNEETYELPSAFLEAAFGKTIKQSGCYF<br>KDETTTIDEAEEASHELYCERAQIKDGQTVLDIGCGQGGLVLHIAQKYKNC<br>HVTGLTNSKAQKNYILMQAEKLQLSNVDVILADVTKHESDKTYDRILVIETIE<br>HMKNIQLFMKKLSTWMTEDSLLFVDHICHKTFSHHFEAIDEDDWYSGFIFPK<br>GCVTILSASALLYFQDDVTILDHWVVNGMHMARSVDAWRKKLDKNMELAR<br>EILLPGLGSKEAVNGVITHIRTFCMGGYEQFSYNNGEEWMVAQMLFKKK
	GfNMT3 ([13])	MDSKEVGIMKKESAEEILGRVMKGQIEDEELRELIKFQFERLLQWGYKPTL<br>QQQLAFNLDFLKSLKELEMSGETEAMNKETYELPTAFLEAIFGKTIKISACYF<br>KDELMTLDEAEEACHELYCERAQIRDGQTILDIGCGQGSLILHIAQKYKNCHI<br>TGITNSNGQKNYITTQAEKLQLSNVDVILADVTKFDMEKTFDRVSVIGTIEHM<br>KNMALFMKKVSSWMKEDGLLFVDHVCNKTFSHHYEALDEDDWYSGYKFP<br>KGSVTILWANALLYFQDDVSVVDHWLLNGMHMARTQVEWGKKLNKNIEAV<br>KEILEAGLGSKEAANQVITHMRTCGIGGYEQFSYNNGEEWMISHVLFKKK
Nelumbo nucifera	NnCNMT (AXJ91467)	MDALIQVPYDATVRLMLSSLERNLLPDVVIRRLTRLLLASRLRWGYKPSSQL<br>QLSDLLQFVHSLKDMPIAIKTDLPKSQHYELPTSFFKLVLGKNLKYSCCYFL<br>DKSSTLKDAEKAMLELYCERAQIKDGQSVLDVGCGWGSLSLYIAQKFSSC<br>RITGICNSKTQKAYIEEQCRELKLQNVEIIVADISTFEMEASFDRILSIEMFEH<br>MKNYKALLNKISKWMKEDSLLLVNYFCHKAFAYHFEDKNEDDWITRYFFTG<br>GTMPAANLLLYFQDDVSVVNHWLVNGNHYARTSEEWLKRMDQNMASIKPI<br>MESTYGKDSAVKWTAYWRTFFISVAELFGYNNGEEWMVALFLFKKKIN
Papaver bracteatum	PbTNMT (ACO90237)	MGSIDEVKKESAGETLGRLLKGEIKDEELKKLIKFQFEKRLQWGYKSSHQE<br>QLSFNLDFIKSLKKMEMSGEIETMNKETYELPSEFLEAVFGKTVKQSMCYF<br>KHESATIDEAEEAAHELYCERAQIKDGQTVLDIGCGQGGLVLYIARKYKKCH<br>VTGLTNSKAQVNYLLKQAEKLGLTNVDAILADVTQYESDKTYDRLLMIEAIE<br>HMKNLQLFMKKLSTWMTEESLLFVDHVCHKTFAHFFEAVDEDDWYSGFIF<br>PPGCATILAANSLLYFQDDVSVVDHWVVNGMHMARSVDIWRKALDKNME<br>AAKEILLPGLGGSHEAVNGVVTHIRTFCMGGYEQFSMNDGDEWMVAQLLF<br>KKK
Papaver somniferum	PsCNMT (AAP45316)	MQLKAKEELLRNMELGLIPDQEIRQLIRVELEKRLQWGYKETHEEQLSQLL<br>DLVHSLKGMKMATEMENLDLKLYEAPMEFLKIQHGSNMKQSAGYYTDEST<br>TLDEAEIAMLDLYMERAQIKDGQSVLDLGCGLGAVALFGANKFKKCQFTGV<br>TSSVEQKDYIEGKCKELKLTNVKVLLADITTYETEERFDRIFAVELIEHMKNY<br>QLLLKKISEWMKDDGLLFVEHVCHKTLAYHYEPVDAEDWYTNYIFPAGTLT<br>LSSASMLLYFQDDVSVVNQWTLSGKHYSRSHEEWLKNMDKNIVEFKEIMR<br>SITKTEKEAIKLLNFWRIFCMCGAELFGYKNGEEWMLTHLLFKKK
	PsRNMT (AOR51552)	MSTTMETTKISQQDDLWKNMELGQISDEEVRRLMKIGIEKRIKWGTKPTQQ<br>EQLAQLLDFNKSLRGMKMATEIDTLENHKIYETPESFNQIIGGKESAGLFTD<br>ETTTTMEEANTKMMDLYCERAGLKDGHTILDLGCGAGLLVLHLAKKYKKSK<br>ITGITNTSSHKEYILKQCKNLNLSNVEIILADVTKVDIESTFDRVFVIGLIEHMK<br>NFELFLRKISKWMKDDGLLLLEHLCHKSFSDHWEPLSEDDWYAKNFFPSG

		TLVIPSATCLLYFQEDVTVIDHWILSGNNFARSNEVILKRIDGKIEEVKDIFMS<br>FYGIGREEAVKLINWWRLLCITANELFKYNNGEEWLISQLLFKKKLMTCI
	PsTNMT<br>(AAY79177)	MGSIDEVKKESAGETLGRLLKGEIKDEELKKLIKFQFEKRLQWGYKSSHQE<br>QLSFNLDFIKSLKKMEMSGEIETMNKETYELPSEFLEAVFGKTVKQSMCYF<br>THESATIDEAEEAAHELYCERAQIKDGQTVLDIGCGQGGLVLYIAQKYKNC<br>HVTGLTNSKAQVNYLLKQAEKLGLTNVDAILADVTQYESDKTYDRLLMIEAI<br>EHMKNLQLFMKKLSTWMTKESLLFVDHVCHKTFAHFFEAVDEDDWYSGFI<br>FPPGCATILAANSLLYFQDDVSVVDHWVVNGMHMARSVDIWRKALDKNME<br>AAKEILLPGLGGSHETVNGVVTHIRTFCMGGYEQFSMNNGDEWMVAQLLF<br>KKK
Stephania intermedia	SiCNMT1<br>(QFU85193)	METLLQVPYNVTVKMLLGSLERALLPDMVVRRLTRLLLAARLRQGYKPSSQ<br>LQLYDLLHFAQSLQDMPIAIRTDKAKEQHYELPTSFFNLVLGKNMKYSCCY<br>FLDKTSTLEDAENAMLELYCERAQLKDGHTVLDVGCGWGSLSLYIAQKYT<br>NCRVTGICNSMTQKACIEEKCRELQVHNVEIIVADISTFEMEGTFDRIFSIEM<br>FEHMKNYKELLKKISKWMTQEGLLFVHYFCHKTFAYHFEDLSDDDWITRYF<br>FTGGTMPSANLLLYFQEDVSILNHWLVNGKHYSQTSEEWLKRMDRNLASI<br>KPIMESTYGKAEAVKWTVYWRTFFIAVAELFGYNNGEEWMVAHFLFKKK
	SiCNMT2<br>(QFU85194)	EAPQRNRAEVTEVMRKLGLGLIPDEELRSLISVQVERRLRWGYKPTFEQQL<br>AQLVQFVHSLKQMSISLEAEVLESQVYEIPNSFMKLLHGSSMKASWCFFIN<br>DSTTLDEAEIAMLELYCDRSQIRDGDRVLDLGCGFGALATYIARKYPNCQV<br>TGVTNSEFQKEFIEEQCKKDNLVNVEVILADVTTLEMDKEFDRVMAIGVIEH<br>MKSYELLLKKISKWMKQDGLLFVDHICHKAFAYHFEPIGEEDWIEEYIFPGG<br>VMTIPSADLLLYFQDDISVVNHWAVNGKHYSRTNEEWLKRLDGNADAARAI<br>LEDSLGSKEEAMKMLNYWRTFCFYGMELCKYNNGEEWMSAHVLFKKK
	SiCNMT3<br>(QFU85195)	MEAKQGKKDELKSKVAELLERPELGLVPDEEIRRLAKARLEKRLKWGYKPT<br>HEEQLSNLLQFVHSLPSLNMASEDDSPKAWLYETPTSFLQLIYGDIIKESGS<br>YYNDESSTLEEAMIHNMDLCCERASIKEAHSVLDLGCGYGAFILHVAQKYK<br>TCNVTGITSSISQKNYIIEQCKKLNLSNVEVILADVATIKLDTTFDRVFAAGMF<br>EHINDYKSFLRKISKWMKPDGLLFVEHLCNKTFPYQNKPLDDGDNWGEYV<br>FPSGGLIIPSASLLLYFQEDVSIVNHWTFSGKHAANKFEELLKRIDAKIEAIKG<br>IFNECYGSKDAVRFINYWRVFLITAGEMFGYNNGEEWMGVHVLFKKK
Thalictrum flavum	TfCNMT<br>(AAU20766)	MAVEGKQVAPKKAIIVELLKKLELGLVPDDEIKKLIRIQLGRRLQWGCKSTYE<br>EQIAQLVNLTHSLRQMKIATEVETLDDQMYEVPIDFLKIMNGSNLKGSCCYF<br>KNDSTTLDEAEIAMLELYCERAQIKDGHSVLDLGCGQGALTLYVAQKYKNS<br>RVTAVTNSVSQKEFIEEESRKRNLSNVEVLLADITTHKMPDTYDRILVVELF<br>EHMKNYELLLRKIKEWMAKDGLLFVEHICHKTFAYHYEPIDEDDWFTEYVF<br>PAGTMIIPSASFFLYFQDDVSVVNHWTLSGKHFSRTNEEWLKRLDANVELI<br>KPMFVTITGQCRQEAMKLINYWRGFCLSGMEMFGYNNGEEWMASHVLFK<br>KK
	PavNMT<br>(ACO90251)	METKQTKKEAVANLIKRIEHGEVSDEEIRGMMKIQVQKRLKWGYKPTHEQ<br>QLAQLVTFAQSLKGMEMAEEVDTLDAELYEIPLPFLHIMCGKTLKFSPGYFK<br>DESTTLDESEVYMMDLYCERAQIKDGQSILDLGCGHGSLTLHVAQKYRGC<br>KVTGITNSVSQKEFIMDQCKKLDLSNVEIILEDVTKFETEITYDRIFAVALIEH<br>MKNYELFLKKVSTWIAQYGLLFVEHHCHKVFAYQYEPLDEDDWYTEYIFPS<br>GTLVMSSSSILLYFQEDVSVVNHWTLSGKHPSLGFKQWLKRLDDNIDEVKE<br>IFESFYGSKEKAMKFITYWRVFCIAHSQMYSTNNGEEWMLSQVLFKKK
**CYP80**		
Berberis stolonifera	BsCYP80A1<br>(AAC48987)	MDYIVGFVSISLVALLYFLLFKPKHTNLPPSPPAWPIVGHLPDLISKNSPPFL<br>DYMSNIAQKYGPLIHLKFGLHSSIFASTKEAAMEVLQTNDKVLSGRQPLPCF<br>RIKPHIDYSILWSDSNSYWKKGRKILHTEIFSQKMLQAQEKNRERVAGNLV<br>NFIMTKVGDVVELRSWLFGCALNVLGHVVFSKDVFEYSDQSDEVGMDKLI<br>HGMLMTGGDFDVASYFPVLARFDLHGLKRKMDEQFKLLIKIWEGEVLARR<br>ANRNPEPKDMLDVLIANDFNEHQINAMFMETFGPGSDTNSNIIEWALAQLIK<br>NPDKLAKLREELDRVVGRSSTVKESHFSELPYLQACVKETMRLYPPISIMIP<br>HRCMETCQVMGYTIPKGMDVHVNAHAIGRDPKDWKDPLKFQPERFLDSDI<br>EYNGKQFQFIPFGSGRRICPGRPLAVRIIPLVLASLVHAFGWELPDGVPNEK<br>LDMEELFTLSLCMAKPLRVIPKVRI
Coptis japonica	CjNMCH<br>(BAB12433)	MEVLSIAIVSFSFLLFLFFILRDSRPKNLPPGPRPSPIVGNLLQLGDKPHAEF<br>AKLAQKYGELFSLKLGSQTVVVASSPAAAAEILKTHDKILSGRYVFQSFRVK

		EHVENSIVWSECNDNWKLLRKVCRTELFTPKMIESQSEIREAKAREMVKFL<br>RGKEGEVVKIVEVVFGTLVNIFGNLIFSKDVFDLEDPTGGSVELKEHLWKLL<br>DMGNSTNPADYFPIMGKLDLFGQRRAVAEVLQQIYDVWGVMLKERRGTK<br>GSESKNDFVDVLLNAGLDDQKINALLMELFGAGTETSASTIEWAITELTKKP<br>LVVSKIRLELVNVVGDNTVKESDLPHLPYLQAFVKETLRLHPPTPLLLPRRA<br>LETCTVMNYTIPKECQIMVNAWAIGRDPKTWDDPLNFKPERFLSSDVDYKG<br>NDFELIPFGGGRRICPGLPLASQFSNLIVATLVQNFEWSLPQGMSTSELSM<br>DEKFGLTLQKDPPLLIVLKARASNI
	CjCYP80G2<br>(BAF80448)	MDLQIALFSLIPVILVFILLLKPKYKNLPPGPHPWPLIGNLPILFTNTEVPLHITL<br>ANMARTHGPIMILWLGTQPTVMASTAEAAMEILKTHDRIFSARHIRMSFRLK<br>HHIKYSLVWSDCTDYWKLLRKIVRTEIFSPKMLQAQSHVREQKVAELIDFLR<br>SKEGQVVKISQFVFGTLLNILGNVVFSKDVFVYGDETDKGGIQNLIREMLMI<br>GAEPNVAEFYPSLEELDLQGLKKKCDERFIRVMKMWEGTVKERKANRNEE<br>SKDMLDVLLANDFNDAQINALFLETFGPGSETSSATIEWVIAELIKSPKEMA<br>KVRKELNEVVGTSTIKESDLPQLPYLQACIKEAMRLHPAAPFLLPRRAAETC<br>EVMGYTIPKNSQVLVNAYAIGRDPKSWKDPSTFWPERFLESDVDFHGAHY<br>QFIPFGSGRRTCVGMPLATRTIPLIVGSLVHNYDFGLPGGNRPEDLKMNEM<br>LSLTLAIDPSLCVVPKARA
Corydalis yanhusuo	CyNMCH ([14])	MEILSIAILSLVISTLIYYIFFSDSKALKGLPPGPKPWPIVGNLLQLGEKPHSQF<br>AEMAQTYGDLFTLKMGTQTVVVASTPSSAIEILKTHDRLLSARYVFQSFRVP<br>NHVENSMVWSDCNEVWKMLRKVCRTELFTPKMIESQAHVRESKAMEMVK<br>FLKGKQGGEVKIAEVVFGTLVNIFGNLIFSQDVFELGDPSGGSAEMKEHLW<br>RMLELGNSTNPADYFPILGRFDLFGQRKDVAECLRLIYDVWGVMLKERKVK<br>KAAQGKKGNDNNDFVDVLLDSGLDDFQINALLMELFGAGTETSSSTIEWAI<br>AELTKNPHITAKIRAELESVVGQSPIKESDIPNLPYLQACTKETLRLHPPTPLL<br>LPRRAIETCQVMNYTIPKDCQIMVNAWAIGRDPKIWKDALKFSPERFLNSSV<br>DFKGNDFELIPFGAGRRICPGVPLATQFISLIVATLVQNFEWNLPNGMDPKD<br>LVMDEKFGLTLQKEPPLYIVPKSRV
Eschscholzia californica	EcNMCH<br>(AAC39453)	MEVVTVALIAVIISSILYLLFGSSGHKNLPPGPKPWPIVGNLLQLGEKPHAQF<br>AELAQTYGDIFTLKMGTETVVVASTSSAASEILKTHDRILSARYVFQSFRVK<br>GHVENSIVWSDCTETWKNLRKVCRTELFTQKMIESQAHVREKKCEEMVEY<br>LMKKQGEEVKIVEVIFGTLVNIFGNLIFSQNIFELGXPNSGSSEFKEYLWRML<br>ELGNSTNPADYFPMLGKFDLFGQRKEVAECLKGIYAIWGAMLQERKLAKK<br>VDGYQSKNDFVDVCLDSGLNDYQINALLMELFGAGTETSASTIEWAMTELT<br>KNPKITAKLRSELQTVVGERSVKESDFPNLPYLEATVKETLRLHPPTPLLLP<br>RRALETCTILNYTIPKDCQIMVNAWGIGRDPKTWIDPLTFSPERFLNSSVDF<br>RGNDFSLIPFGAGRRICPGLPIANQFIALLVATFVQNLDWCLPNGMSVDHLI<br>VEEKFGLTLQKEPPLFIVPKSRV
Nelumbo nucifera	NnCYP80P1<br>(XP_010241290)	MEIMAQAALAGEVINLLFPVFLSLCLFFLFIKLIKSASSATGPPLPPGPHPWP<br>VVGNIFRLGRKPHVRLSQLAQVHGPLMSLSLGRQLIVVASSPATATQILKTH<br>DKILCGRYAPVSSRRNDQLKYLDNFLWTELFSTRAIDSQAALREKKVRELIR<br>YLGSKEGEVVNIGEVMFATVFNILCNLFLSKDFISLEDDEIMKGGIKRLLRSIS<br>EVASTPNLADLFPILGPLDIQGLNKKARELFVKITAMWEDIIKERREAQSAGG<br>HVSRQRDLLDVLIDNNFSDDQINNLLLKLFTAGTHTSSSTIEWAMAEMMKN<br>QESLVKARIELAREIKRENQVREADLCNLVYLNACLKETLRLHPPAPFLLPH<br>RAIKTCTVMNYTIPKDSQVFVNVWAIGRDSMAWSNPLSFNPERFLSSNLGF<br>MGNNFEFIPFGAGRRICPGLPMAGKQIQLIMASLIYCFNWSLPNGTHPSTLN<br>MNDKLGVVLQREQPLLLIPKLRRRNY
	NnCYP80Q1<br>(AXJ91462)	MALLVSFIFFLLSILSVVLFLKPSPNNLPPGPFSWPIIGTLLPKLKKQPHVELS<br>KLAQRFGPLMLLKFGVEPVVVASSHVAAVEVLKNQDRLLSGRFAPHSVRIK<br>GYIEHSMVWADCTDYWKMVRKVWRTELFSTKMLDIQAHAREEKVSELMK<br>FLIRKEGEKVNFADVIFGSILNILGALIFSKDVYDFEDRTDNNLGMKGMIRQL<br>MILAAIPNLADLYPILGGSDFQGLRKASAACVKRMNESWAAIVKQRRKNDD<br>HSKNDFLQVLLDSGFSDPQIDAMLLETFGPGSDTSTSTIEWAMAELLRNPE<br>KLVKVREELDRVIRRSNNVKESDLPNLPYLHACVKETLRLHPPVTFLLPHRA<br>METCQMMNYTIPKGCQLMVNTYAIGRDSKTWEKPLSFLPERFLNSELDYQ<br>GNDFQYIPFGAGRRICPGLSLATRVVRLILASLLHTFDWSLPDGMHPDELD<br>MNDKFGLALQKDIPLVVIPKLRK

	NnCYP80Q2<br>(XP_010242988)	MALLALFILFLLSILSLVLFLKPSSKKLPPGPFSWPIIGTQLPSPTKKPNLELVK<br>LAQRYGPLMLFRFGLENVVVASNHVAAMEVLKNQDRVLSGRYKGNSVRV<br>KGYIEYSMVWADCTDYWKMVRKILRTELFSTKMLDVHAHAREEKVSELMK<br>FLRRKEGEEVNFVDVIFGCILNMLGALIYSKDVYDFEDKTDINLGMKGMIRQ<br>LMILAATPNIADLYPIFFDGSDFQGLRKESAACVKRMSESWAAIINERRKNN<br>DHTKNDLLQVLLDSGFSDPQIDAMFLETFGPGSDTSASTIEWALAELLRNP<br>EKLVKLHEELDRVIGRNNTVKDSDLPNLPYLHACVKETLRLHPPVPFLIPHIA<br>LESCEVMNYTIPKGSEVLVNLYATGRDPTTWDNPNSFLPERFLNSEVDYQ<br>GNHFQYIPFGAGRRMCPGMSLGTRVVRLILAALVHTFDWSLPGGMHQDEL<br>DMADRFGVGFQKETPLVVIPTLRK
Papaver somniferum	PsNMCH<br>(AAF05621)	MEIVTVSLVAVVITTFLYLIFRDSSPKGLPPGPKPWPIVGNLLQLGEKPHSQF<br>AQLAETYGDLFSLKLGSETVVVASTPLAASEILKTHDRVLSGRYVFQSFRVK<br>EHVENSIVWSECNETWKKLRKVCRTELFTQKMIESQAEVRESKAMEMVEY<br>LKKNVGNEVKIAEVVFGTLVNIFGNLIFSQNIFKLGDESSGSVEMKEHLWRM<br>LELGNSTNPADYFPFLGKFDLFGQSKDVADCLQGIYSVWGAMLKERKIAKQ<br>HNNSKKNDFVEILLDSGLDDQQINALLMEIFGAGTETSASTIEWALSELTKN<br>PQVTANMRLELLSVVGKRPVKESDIPNMPYLQAFVKETLRLHPATPLLLPR<br>RALETCKVLNYTIPKECQIMVNAWGIGRDPKRWTDPLKFSPERFLNSSIDFK<br>GNDFELIPFGAGRRICPGVPLATQFISLIVSSLVQNFDWGFPKGMDPSQLIM<br>EEKFGLTLQKEPPLYIVPKTRD
Thalictrum flavum	TfNMCH<br>(AAU20767)	MEVLSAAMVSLFFIFLFIFFILCDSRNKDLPPGPRPSPIVGNLLQLGEKPHAE<br>FAKLAEKYGELFTLKLGSQTVVVASSPAAAAEILKTRDKILSGRYVFQSFRV<br>YEHVLNSIVWSECNENWKLLRKVCRTELFSPKMIESQAYIREAKALDMVRF<br>LRKKENQEVKIVEVVFNTLVNIFGNLIFSKDVFDLEDPTGGSAELKEHLWKL<br>LDMGNSTNPADYFPIMGKFDLFGQRREVAKVLKQIYDVWGVMLKERRSIT<br>GHRENDFANVLLNAGLDDQKINALLMELFGAGTETTASTIEWAITELTNNLR<br>VISKLRAELINVVGHKTVKESNIPHLPYLQAIVKETLRLHPPTPLLLPRRALET<br>CKVMNYTIPKECQIMVNAWAIGRDPKTWDDPLTFKPERFMNSTVDYKGND<br>FELIPFGGGRRICPGLPLASQFLSLIVATLVQNFDWSFPQGMTPNEVPMDE<br>KFGLPLQKDPPLLIVLKARTSMKLQD
**CYP719A**		
Argemone mexicana	AmCYP719A13<br>(ABR14721)	MEEKIMTNNSPWILTSSTTTTTTILLSLLFTIFIILRRNKSSSSKMVWPTGPKT<br>LPIIGNMNILGGTALHVVLHNLAKTYGNVMTIWIGSWRPVIVVSDIDRAWEVL<br>VNKSSDYSARDMPEITKLATADWKTISSSDSGPFWTNLRKGLQNVALSPQ<br>NLSSQSKFQERDIIKTIQNLKEEAKMNNGIVKPLDHLKKAMVRLISRLIYGQD<br>FDNDEYVEEMHHTIEELIRVSGYARLAEAFYYAKYLPSHKKAVREVLQANQ<br>RVQNLVRPLLSLNSPTNTYLHFLRSQNYEDEVIIFAIFEAYLLGVDSTSSTTA<br>WALAYLIREPNVQEKLYEELKNFTNDNDRKMVKFEDLNKLQYLQAVVKET<br>MRMKPIAPLAIPHKACRETSLMGRKVNQGTRVMVNIYALHHNQNVWKEPY<br>KFNPERFLQKNQDGVDGKAMEQSLLPFSAGMRICAGMELGKLQFSFALAN<br>LVNAFKWSCVSDGVFPDMSDQLGFVLLMKTPLEAGIVPRM
	AmCYP719A14<br>(ABR14722)	MDETIWLIISTVIIVLGIAKFLLGKSSSSSLSTMEWPVGPKKLPIIGNLHQLGG<br>DVFHVVLANLAKVYGSVFTIWVGSWRPMIIVSDIDKAWEVLVNKSSDYSAR<br>DMPDITKIISANWKNISCSDSGPFWHNLRKGLQGVALTPLNVASQYHLQER<br>DMKNLINSMYKDASRKNGILKPLDYLKEETVRLLSRLIFGQDFQDEKLVVG<br>MHHALDDLVRISGYASLADAFKFCENLPSHKKSIREVHEVKKRVENLIRPHI<br>VSNPPTNTYLYFLKTQDFNEDIIISAILEVYDLGVDSTASTTVWALTFLVREQ<br>EIQEKLYREIVNVTGGKRSVKVEDVNKMPYLQAVMKETMRMKPIAPMAIPH<br>KTSKDTSLMGKKINKGSVIMVNLYAIHHNPKVFPEPYKFMPERFLKDVNSD<br>ESLGNIKTMESSLLAFSAGMRICAGMELGKLQLAFGLASLVHEFKWSCSVD<br>GKLPDLSEDHCFILLMKNPLEAKITCRIH
Chelidonium majus	CmCYP719A_SPS<br>(ASG81439)	MEELMSDPWIITATTLAVVILLTTILRRKSSSMKWPKGPKTLPIIGNMHLFGG<br>TDLHVVLYNIAKVYGNVMTIWIGSWRPVIVISDIERAWEVLVNKSSDYSARD<br>MPEITKLISSDWKTISTSDAGAFWTNLRKGLQNVALSPHNLASQFQFQESDI<br>KKLIETIKEEAAVNNGIVKPLDHLKKAMVRLISRLVFGQDFDNDEYVEGMHH<br>AIEELIRVSGYARLAEAFYYAKYLPSHKKAVRDVEEANRRVQKLVRPFLSLN<br>PPTNTYLHFLRSQNYSEEVIIFAIFEAYLLGVDSTSSTTAWALAFLIREPNVQ<br>EKLYQELKNFTVKNGRDMLKVEDINKLPYLQATIKETMRMKPIAPLAIPHKA<br>CRDTSLKGSKIDKGTRVMVNIYALHHNEKIWKEPFKFMPERFLQTEGENGK

		AMEQSFLPFSAGMRICAGMELGKLQFNFALANLVNAFKWSCVADGVLPDM<br>SDQLGFVLVMKTPLEARITSRC
	CmCYP719A_CFS<br>(ASG81438)	MEESFWLVAATVLVVFVVAKLLFRKSSSISTMEWPAGPKTLPIIGNLHQLGG<br>AALHVVLANLAKVYGSVMTIWVGAWRPMIVISDIDKAWEVLVNKSSDYSAR<br>SLPEITRIISANWKNIMTSDSGPFWQNLRKGLQGGALSPHNVMSQYQLQE<br>RDMQNLIKTMRVEASKNNGRIKPLDHLKQETVRLLSRLIFGQDFNDEKLVV<br>GMHHALDDLVRISGYASLADAFKVAENLPSHKKSIREVHELKRRVENLVRP<br>HIVSNPPTNTYLNFLLSQNFSEDLIISAILEVYDLGVDSTASTTVWALTFLVRE<br>QKVQEKLYQEIKNLTGGRSTVKVEEVSKMPYLQAVMKETMRMKPIAPMAIP<br>HTAARETSLMGKKIDKGTVVMVNLYAIHHNPNIFPEPYKFMPERFLHGEEQ<br>NGGNIKEMEQSLLPFSAGMRICAGMELGKLQLGFALASLVNAFKWECAAD<br>GKLPDLSEDHCFILLMKNPLEAKITPRTH
Coptis chinensis	CcCYP719A<br>(AGL76711)	MEMSPLLVCATVAIVFATTTIIRILFSSSSLPQMKWPSGPRKLPIIGNLHQLG<br>DDVLHVALAKLAKVHGSMMTIWIGSWRPVIVISDLEKAWEVLVNKSADYGA<br>RDMPEITKIASASWHTISTSDAGPFWQNVRKGLQSGAMGPFNVAAQNQH<br>QERGMKRLIKAMSDEAANNNGIVKPLDHIKKNTVRLLTRLIFGQNFDDNKFI<br>ESMHYEIEDIIRISGYARLAEAFYYAKYLPSHKKAEREAFLVKCRVQELVRPL<br>LSSNPPTNSYLYFLLSQNFEEEVIIFCIFELYLLGVDSTSSTTTWALAYLIREQ<br>GAQEKLYQDIRMTLGDVDLVKIEDVNKLKYLQGVVKETMRMKPIAPLAIPHK<br>TAKETTLMGTKVAKGTRIMVNLYALHHNQNIWPDPYKFMPERFLEGATGTA<br>YNKAMEQSFLPFSAGMRICAGMDLGKLQFAFALANLVNAFKWSCVEEGKL<br>PDMGEELSFVLLMKTPLEARIAGRNV
Coptis japonica	CjCYP719A1<br>(BAB68769)	MEMNPLLVCATVAIVFATTTIIRILFSSSSLPQMKWPSGPRKLPIIGNLHQLG<br>DDVLHVALAKLAKVHGSVMTIWIGSWRPVIVISDIEKAWEVLVNKSADYGAR<br>DMPEITKIASASWHTISTSDAGSFWQNVRKGLQSGAMGPLNVAAQNQYQE<br>RDMKRLIKAMSDEAANNNGIVKPLDHIKKNTVRLLTRLIFGQAFDDNKFIES<br>MHYEIEDIIRISGYARLAEAFYYAKYLPSHKKAEREAFLVKCRVEELVRPLLS<br>SKPPTNSYLYFLLSQNFEEEVIIFCIFELYLLGVDSTSSTTTWALAYLIREQGA<br>QEKLYQDIRMTLGDVDLVKIEDVNKLKYLQGVVKETMRMKPIAPLAIPHKTA<br>KETTLMGTKVAKGTRIMVNLYALHHNQNIWPDPYKFMPERFLEGETGTAY<br>NKAMEQSFLPFSAGMRICAGMDLGKLQFAFALANLVNAFKWSCVEEGKLP<br>DMGEELSFVLLMKTPLEARIAGRNV
Eschscholzia californica	EcCYP719A2<br>(BAD98250)	MEEMKILMMNNPWILTATATTLLISIFLFFTRKSSKMVWPAGPKTLPIIGNMH<br>LLGGTALQVVLHNLAKVHGSVMTIWIGSWRPVIVVSDIERAWEVLVNKSSD<br>YSARDMPDITKIISADWKTISTSDSGPHWTNLRKGLQNVALSPHNLAAQFQ<br>FQEKDMTKMIQTLEEEARNNNGIVKPLDHMKKATLRLISRLVFGQDFNNDK<br>YVDDMHLAIEELIRVSGYARLAEAFYYAKYLPSHKKAVREVEEAQRRVQNL<br>VSPFLSLNPPTNTYLHFLRSQKYDDEVIIFAIFEAYLLGVDSTSLTTAWALAF<br>LIREPNVQEKLYQELESFASKNDRRILKVEDINKLQYLQAVIKETMRMKPIAP<br>LAIPHKACRDTSLMGKKIDKGTRVMVNIFALHHNKNVFNDPFKFMPERFMK<br>VDSQDANGKAMEQSLLPFSAGMRICAGMELGKLQFSFALANLAYAFKWSC<br>VADGVLPDMSDQLGFVLLMKTPLEARINRRN
	EcCYP719A3<br>(BAD98249)	MEEMKFLIMNNPWVLFATSATLLISIFLFFRRKSPNMAWPEGPKTLPIIGNM<br>HLLGGTALQVVLYNLAKVHGRVMTIWIGSWRPVIVVSDIEQAWEVLVNKSS<br>DYSARDMPDITKIVTADWRTISTSDSGPHWSNLRKGLQNIAISPNNLAAQFQ<br>FQEKDIIKMIQILEQEAKDNNGIVKPLDHLKKATIRLISRLVFGRDFEEDKYVE<br>DMHHAIEELIRISGYARLAEAFYYAKYLPSHRKAVRYVEELKQIVKNLIRPFL<br>SVNPPTNTYLHFLRSQNYDEEVVIFAIFETYLLGVDSTSSTTAWALAYLVRE<br>PSVQDRLHQELDHFAKQNDRKILKVEDMNKLQYLQAVIKETMRMKPIAPLAI<br>PHKACKDTSLMGNKINKGTRVMVNLYALHHNKNVFNDPFKFMPERFLKVD<br>NQDAKGKAMEQSLLPFSAGMRICAGMELGKLQFSFALANLIFAFKWSCVD<br>DGVLPDMSDELGFVLLMKTPLKARINPRN
	EcCYP719A5<br>(BAG75113)	MEESLWVVTATVVVVFAIAKLLKKSSSISTMEWPKGPKKLPIIGNLHQLGGE<br>AFHVVLANLAKIHGTVMTIWVGAWRPMIVISDIDKAWEVLVNKSSDYAGRD<br>FPEITKIISANWKNISCSDSGPFWQNLRKGLQGGALAPLNVISQYQLQERD<br>MKNLITSMQEKASKNNGILKPLDYLKEETIRLLSRLIFGQSFNDENFVKGVHL<br>ALDDLVRISGYASLADAFKFCENLPSHKKSIREVHEVNERVVNLVKPYLVKN<br>PPTNTYLYFLNSQKFSDEVIISAVLEVYDLGVDSTASTAVWALTFLVREPRV<br>QEKLYKEIIDLTGGERSVKVEDVSKLPYLQAVMKETMRMKPIAPMAIPHKTS

		RDTSLMGKKVNKGTSIMVNLYAIHHNPKVFPEPYKFIPERFLQGQESKYGDI<br>KEMEQSLLPFSAGMRICAGMELGKLQYGFSLASLVEAFKWTCAVDGKLPD<br>LSEDHCFILLMKNPLEARITPRTQL
	EcCYP719A9<br>(BAG75114)	MEEMKFLMMNNPWVLTATFTTLLISIFLFVTRKTSNTEWPNGPKTLPIIGNM<br>HLLGGTKALQVILHNLAKVYGGVMTIWIGSWRPVIIVSDIERAWEVLVNKSS<br>DYSARELPEIIKYNAANFRTIATCDSGPHWSNLRKGLQNVALSPHNLAAQF<br>QFQEKDITKMIQNLEVEAKNNNGIVQPLDHLKKATIRLISRLIFGQDFDEDTY<br>VEKLHHTIDELIRMSGYAQLAEAFYYARYLPNHKKAVNHAAKTKQIVTHLMR<br>PFLSLNPPTNSYLHFLQSQNYDEELIIFSIFEVYLLGVDSTSSTTAWALAYLV<br>REPNVQETLYQELDNFAKQNDRKILKVEDINKLQYLQAVTKETMRMKPIAPL<br>AIPHKASRDTTLMGKKVEKGTKVMVNLHALHHNENVFNDPYKFMPERFLK<br>SYQGAKAKAMEQSYLPFSAGMRICGGMEVGKLQFGFALANLAYAFKWSC<br>AVNGVLPDMSDELGFVLFMKTPLEARIVRRN
Nelumbo nucifera	NnCYP719A22<br>(XP_010267084)	MQGNQGLILASVIFVVAIVQMMIRKRRTSPTAMKWPAGPRKLPIIGNMHQL<br>SRADGLFHVALTKLAKVHGSVMTIWLGSWRPTIVVSDDEVAWEVLVNKSS<br>DYAARDHPYIDKIMWAGARTIHTSDASPHWHSLRKGLQSGGLGPLSISGQT<br>HLQEKDIAQMLRDMREEASLNGGLVKLFHHIRRTSVRLLCRLCFGPNFEDA<br>KFSEAIDKAIEDIIRISGVGYLADAFFFGRHFPGLKHTFQEACDLKRRVEDLI<br>RPFLRAVPPPNCYLHFLLSNNIPEDVTIFTILEVFTLGIDSTSSTATWALALLT<br>NEQRVQQKLYQDIKKNIDSTQQIVRVEDVSKLQYLQAAVKETLRLKPVAPLV<br>PHMTATETTLMGTKVAQGTRVVVNLHAIHYNPNVWPEPEKYMPERFMPR<br>QEEVDEIRPGTTKLSYFLPFGGGMRACAGMEVGKLHVGFVIANIVNAFQW<br>SSAVEGQPPDLTEDFKFVLLMKNPLTVRITARHP
Papaver somniferum	PsCYP719A21<br>(AFB74615)	MIMSNLWILTLISTILAVFAAVLIIFRRRISASTTEWPVGPKTLPIIGNLHILGGT<br>ALHVVLHKLAEVYGSVMTIWIGSWKPVIIVSDFDRAWEVLVNKSSDYSARE<br>MPEITKIGTANWRTISSSDSGPFWATLRKGLQSVALSPQHLASQTAHQERD<br>IIKLIKNLKDEAASGMVKPLDHLKKATVRLISRLIYGQDFDDDKYVEDMHDVI<br>EFLIRISGYAQLAEVFYYAKYLPGHKRAVTGAEEAKRRVIALVRPFLQSNPA<br>TNTYLHFLKSQLYPEEVIIFAIFEAYLLGVDSTSSTTAWALAFLIREPSVQEKL<br>YQELKNFTANNNRTMLKVEDVNKLPYLQAVVKETMRMKPIAPLAIPHKACK<br>DTSLMGKKVDKGTKVMVNIHALHHTEKVWKEPYKFIPERFLQKHDKAMEQ<br>SLLPFSAGMRICAGMELGKLQFSFSLANLVNAFKWSCVSDGVLPDMSDLL<br>GFVLFMKTPLEARIVPRL
	PsCYP719A20<br>(ADB89214)	MEKTIFSNPWILTTSTLVVITIITMLMVFKRKKSSTMVWPTGPKTLPIIGNMHV<br>LGGTALQVVLHNLAKIHGNVMTIWIGSWRPVIVVSDIDRAWEVLVNKSSDY<br>SARDMPEITKIISADWKTISTSDSGPGWSNLRKGLQNVALSPHNLAAQSQF<br>QEKDITKMIQSLKKEAASNNGIVKPIDHLKKATLRLISRLIFGQDFDDDKYVD<br>DMHHAIEELIRVSGYAQLAEAFYYAKYLPSHKKAVRDVEEANQRVKKLVRP<br>FLSLNPPTNTYLHFLNSQKYDEEVIIFAIFEAYLLGVDSTSLTTAWALAFLIRE<br>PVVQDKLYQELKNLTAKNDCEIVKVEDLNKLQYLQAVLKETMRMKPIAPLAI<br>PHKACRDTSLKGNKIDQGTQVMVNIYALHHNEKVWKEPFKFMPERFLQTQ<br>DVVNGKGMEQSLLPFSAGMRICAGMELGKLQFSFSLANLVNAFEWSCVSD<br>GVLPDMSDQLGFVLLMKTPLQARIVPRV
	PsCYP719A25<br>(ADB89213)	MEVTFWLITCGVVVAFALAKLLFGKKSSMSTMEWPSGPKTLPIIGNLHQLG<br>GEAFHVCLANLAKVYGGVFTIWVGSWRPFIVISDVDKAWEVLVNKSSDYSA<br>RDMPDITKIISANWKNISHGDSGPFWHNLRKGLQGVALTPFNVASQYHLQE<br>RDMQNLIKSMKKKASQKNGILKPLDYVKEETVRLLSRLIFGQDFVDEDFVV<br>GMHQALDELVSISGYASLADAFKFCENLPSHKKTVRAVHAIKIRFDNLIRPHI<br>VSNPPTNTYLHFLLSQDFSEDVIISAILEVYDLGVDSTASTTVWALTFLVREQ<br>KIQEKLYREINNVTGGKKPVKVEDLNKLPYLQAVMKETMRMKPIAPMAIPHK<br>ASKDTSLMGKKINKGAVVMVNLYAIHHNPAVFPEPYKFMPERFLKDANSDG<br>SLGDIKKMESSLLAFSAGMRICAGMELGKLQLAFGLASLVNEFKWDCFAEG<br>KLPDLSEEHCFILLMKNPLEAKITPRIH

Supplementary Table 13: Sequence-specific primers used in this work. Primers were used to (**A**) amplify sacred lotus gene candidates, (**B**) perform quantitative real-time PCR (qRT-PCR), (**C**) construct expression plasmids for yeast transient transformation, and (**D**) linearize the expression vectors. For NCS and MT candidates cDNA cloning into the pRSET-A expression vector, the 5'-3' sequences GGTTCTCATCATCATCATCATCATCAT (forward) and TCCACCAGTCATGCTAGCCATACC (reverse) were added to the corresponding primers shown in (**A**).

Gene	Forward primer (5'-3')	Reverse primer (5'-3')
**(A) Gene amplification**		
NnNCS1	ATGATGATCGGACGTGTAGTTAAC	TTAGTGGTTTCTCCTCTTTGATTTG
NnNCS3	ATGCGTGGGCAAGTAACG	TTAGACGTCACAAGCTTTAGC
NnNCS4	ATGCATGCTGGGCAACTATC	CTACTCTGTTTTAAGTTCAGTGAGATACTTC
NnNCS5	ATGATTCACAGTGTGTCTACTG	TCATTCATAGCATGCTTTAGC
NnNCS7	ATGATGACTGCGCGTGTAACTAACG	TTAGTTGTTGTCGCTCTTGGATTTGG
NnOMT1	ATGGAAATTCAGAAGGAAGGTCAAGC	CTAATAAGGATAGGCCACAATTACAGATTGA ACG
NnOMT5	ATGGAGGAGGACATGAAAGC	TCAAGGGAACACTTCTATAATTGATTCG
NnOMT7	ATGGAGGACATGAAAGCTCAAG	TCAAGGGAAGACTTCTATAATTGATTCG
NnOMT8	ATGGGGCAAGGCGAC	TCAAGGATAGGCCTGAATTAG
NnCNMT	ATGGATGCGTTGATCCAGG	TTAATTTATTTTTTTCTTGAATAGGAACAGTG C
NnCYP80P1	ATGGAAATCATGGCTCAAG	TTAATAATTTCTCCTTCTCAGTTTAG
NnCYP80Q1	ATGGCTCTACTAGTCTCG	TTACTTCCTCAACTTGGGAATGACTAC
NnCYP80Q2	ATGGCTCTACTAGCCTTGTTTATTCTC	TTATTTTCTCAAGGTGGGAATGACG
NnCYP719A22	ATGCAGGGCAATCAGG	TCAAGGGTGACGAGCAGTG
**(B) Gene expression**		
NnNCS1	GACGATATTTGGGCTGTGTATAG	CGTTCCATCACCTTGAAGAAT
NnNCS3	GCGTTGGCACCATTCTATAC	GCTCCTCGTCGTCTATCTTAAT
NnNCS4	ACCTTGGGTTTCGTTTGTATC	TGGCTGACTCATCATCAATTTC
NnNCS5	AGGCCAATGCTTCTCTAGTC	GCTGCTGCTATTAGCTTTCTTC
NnNCS7	GATGTGTGGGCTGTGTATG	GTTCCATCACCCTCAAGAAC
NnOMT1	GGACAAGAGCATAGTGTCAATC	TCCATATGCTCCTCCCTAAC
NnOMT5	CAGTGGGTGGTAAAGAGAGAAG	AGATAACACCCATCGTTGCA
NnOMT7	CTTCATCACCGAACGAGATTAC	CCAGTCTTCTTCGCTTCTTTC
NnOMT8	GAGAGGACTGCCAAAGAATG	GCCTGAATTAGAGAGTGCATAG
NnCNMT	CTGCAAACCTTCTCCTCTATTT	CCACTCCTCACTTGTTCTTG
NnCYP80P1	GCTTGGAGCAACCCATTATC	GCACCAAACGGTATGAACTC
NnCYP80Q1	CGACTGATACTCGCTTCTCT	GAGCCAGCCCAAACTTATC
NnCYP80Q2	AGGACTCTGATCTGCCTAAC	GCTATGTGAGGAATAAGGAATGG
NnCYP719A22	TTGTTTCATGTGGCCCTAAC	GTCGTCGCTCACAACAATAG
Nn*B*-Actin	AGGGAGAAGATGACCCAGATTA	GTTGTTCTACCACTGGCGTATAG
**(C) Gene cloning for yeast transient transformation**		
NnCYP80P1	CATAAACAAACAAAGGATCCATGGAAATCAT GGCTCAA	GGATCTTAGCTAGCCGCGGTTAGTGGTGAT GGTGATGATGATAATTTCTCCTTCTCAGTT
NnCYP80Q1	CATAAACAAACAAAGGATCCATGGCTCTACT AGTCTCGTT	GGATCTTAGCTAGCCGCGGTTAGTGGTGAT GGTGATGATGCTTCCTCAACTTGGGAAT
NnCYP80Q2	CATAAACAAACAAAGGATCCATGGCTCTACT AGCCTTGTTTATTCTC	GATCTTAGCTAGCCGCGGTTAGTGGTGATG GTGATGATGTTTTCTCAAGGTGGGAATGAC
NnCYP719A22	CATAAACAAACAAAGGATCCATGCAGGGCA ATCAGGGTTT	CTTAGCTAGCCGCGGTCAAGCGTAATCTGG AACATCGTATGGGTAAGGGTGACGAGCAGT
**(D) Vector linearization for SLIC**		
pRSET-A	GGTATGGCTAGCATGACT	ATGATGATGATGATGATGAGA
pEV2	CCGCGGCTAGCTAAG	GGATCCTTTGTTTGTTTATGTG

Supplementary Information

Supplementary Table 14: Nucleotide sequences of opium poppy genes integrated into YNO-0 yeast strain.

Gene	Nucleotide sequence
BUP1-Cmyc	ATGATCATCGAAACTTTGGATATCTTGGGTCCAAACCAAAACGGTAACTCAGGTACTCATACAC<br>AAAAACCAATTAAAACAAGAAACTGGTTGTTGATCATTATTAACTGTGCTTTAGTTTTCTGTGGT<br>GTTATTGGTGGTCCATTGTTGATGAGATTGTACTACTTGCATGGTGGTTCTAGAAAGTGGTTATC<br>TTCATTTTTGCAAACTGCAGGTTTTCCAGTTTTGATCTTCCCATTGATTTTCTTGTACATTAAACC<br>AAAATTGTCAACACAAAACAACGATCAATCTTCTTCTTTCTTTATGGAACCAAAGTTGTTTTTATG<br>GTCTGCTATTGTTGGTATCGTTTTCGGTGTTTCAAACTTCATGTACGCATTGGGTTTGTCTTACT<br>TGCCAGTTTCAACTTCTACAATCTTGTTCGCTACTCAATTGTGTTTCACAGCTATCTTCGCATGG<br>TTGATCGTTAAGCAAAAGTTTACTGCTTTTATTATTAATGCAGTTATTGTTATGACTTTGGGTTCA<br>ATCTTGTTGGGTATTAATACAAACGGTGACAGACCAATCGGTGTTTCTAAGACTCAATATTTGAT<br>CGGTTTCTTGATGACATTGGCTGCAGCTGCATTAACTGGTTTGGGTACACCATTTGTTGAATTG<br>TCTTTTATTAAGGCTACTAGAAACATCACTTACCCAACATTGTTGCAATTCCAAGTTATTTTGTGT<br>TTGTTCGGTACATGTTTGAACGTTATCGGCATGTTGATTAATAAGGATTTCCAAGCTATCCCAAG<br>AGAAGCAGATATGTTTGAATTAGGTAAATCAAAGTACTACATGATCATCTGTTTAACTGCTTTGA<br>CATGGCAATTATCTGGTATTGGTTTGGTTGGTTTGATCTTGTACACAAACGCATTGTTTAATGGT<br>ATCTATGTTTCAGTTTTGGTTCCTTTTACTGAAGTTGCTGCTGTTATTTTCTTTCATGAAAAGTTT<br>ACTGGTTTGAAGGGTATGGCTTTGGCATTGTGTTTGTGGGGTTTTTCATCTTACTTCTACGGTG<br>AATACAAGATGATGAATAAGGTTGGTGACAATGAAACTCATGAAAAGATTGAAGAAGCTGAATC<br>TGAACCAAAAAGATTGGAAGATCAACAAGCACCATACTCAACAGTTGAACAAAAATTAATTTCTG<br>AAGAAGATTTGTAA
CPR2-Cmyc	ATGGAATCAAACTCTATGAAGTTGTCAATCGTTGATTTGATGTCTGCTATCTTGAACGGTAAATT<br>GGATCAAGCAGATTCAATCTTGATCGAAAACAGAGAAATCTTGATGATCTTGACTACAGCAATC<br>GCTGTTTTTATTGGTTGTGGTTTCTTGTACATCTGGAGAAGATCTTTTAGAAAATCTTCAAAGAT<br>CGTTGAAGTTCCAAAGACAGGTTTTACTAAAGAACCAGAACCAGAAATCGATGATGGTAAAAAG<br>AAAGTTACTATTTTCTTTGGTACTCAAACAGGTACTGCTGAAGGTTTTGCAAAAGCTTTGTCTGA<br>AGAAGCAAAGGCTAGATACGATAAGGCAGTTTTTAAAGTTGTTGATTTGGATGATTACGCTGCA<br>GATGATGATGAATTCGAAGAAAAGTTGAAGAAAGAAAATTTGGCTTTGTTTTTCTTGGCAACATA<br>TGGTGACGGTGAACCAACTGATAACGCTGCAAGATTCTACAAGTGGTTCACAGAAGTTGCTAAG<br>GAAAAGGAACCATGGTTGCCAAATTTGAACTTCGGTGTTTTCGGTTTGGGTAACAGACAATACG<br>AACATTTCAATAAGGTTGCTAAGGTTGTTGATGAAATCATCGTTGAATTAGGTGGTAAAAGATTG<br>GTTCCAGTTGGTTTAGGTGACGATGATCAATGTATCGAAGATGATTTCACTGCTTGGAGAGAAT<br>TGGTTTGGCCAGAATTAGATCAATTGTTGTTGGATGAAAACGATTCAACATCTGTTTCAACTCCA<br>TATGCTGCAGCTGTTGCTGAATACAGAGTTGTTTTTCATGATTCAGCAGATGCTTCTTTGCAAGA<br>TAAGAACTGGTCTAACGCAAACGGTTATGCTGTTTACGATGCATTGCATCCATGTAGAGCAAAT<br>GTTGCTGTTAGAAGAGAATTACATACACCAGCTTCTGATAGATCATGTATCCATTTGGAATTCGA<br>TATTTCAGGTACAGGTTTAACTTACGAAACAGGTGACCATGTTGGTGTTTACTCTGAAAATTGTA<br>TGGAAACTGTTGAAGAAGCTGAAAGATTATTGGGTTTGTCTTCAGATACAGTTTTCTCTATCCAT<br>GTTGATAACGAAGATGGTACTCCAATTGCAGGTTCTGCTTTGCCACCACCATTTCCATCTCCAT<br>CAACTTTGAGAACAGCTTTGACTAAGTACGCAGATTTGTTGAACTTCCCAAAGAAAGCAGCTTTA<br>CATGCTTTGGCAGCTCATGCATCAGATCCAAAGGAAGCTGAACAATTGAGATTTTTGGCATCTC<br>CAGCTGGTAAAGATGAATACGCTCAATGGGTTGTTGCATCTCAAAGATCATTATTGGAAGTTAT<br>GGCTGAATTTCCATCTGCAAAACCACCATTGGGTGTTTTCTTTGCAGCTATTGCTCCAAGATTAC<br>AACCAAGATTCTACTCAATTTCTTCATCTAACAGAATGGCACCATCTAGAATTCATGTTACATGT<br>GCTTTGGTTAATGAAAGAACTCCAGCAGGTAGAGTTCATAAAGGTGTTTGTTCAACATGGATGA<br>AAAATTCTGTTCCATCAGAAGAATCTAGACATTGTTCTTGGGCTCCAGTTTTCGTTAGACAATCA<br>AACTTCAAGTTGCCAGCAGATTCTACAGTTCCAATTATTATGATTGGTCCAGGTACTGGTTTAGC<br>TCCTTTTAGAGGTTTTATGCAAGAAAGATTGGCTTTGAAGGAAGCAGGTGTTGAATTGGGTGCA<br>GCTGTTTTGTTTTTCGGTTGTAGAAACAGATCAATGGATTTCATCTATGAAGATGAATTGAACAA<br>CTTCGTTGAATCAGGTGCTATTTCTGAATTAGTTGTTGCATTTTCTAGAGAAGGTCCAACAAAGG<br>AATACGTTCAACATAAGATGACTGAAAAGGCTTCAGATATTTGGAACATGATTTCTCAAGGTGCA<br>TATTTGTACGTTTGTGGTGACGCTAAGGGTATGGCAAAGGATGTTCATAGAACATTGCATACTA<br>TTGTTCAAGAACAAGGTTCATTAGATTCATCTAAGACTGAAATGTTGGTTAAAAATTTGCAAATG<br>GATGGTAGATATTTGAGAGATGTTTGGGAACAAAAATTAATTTCTGAAGAAGATTTGTAA

Supplementary Information

Supplementary Note 1: Complete nucleotide sequence of pEV2. The pEV2 plasmid was used to transform engineered yeast (*Saccharomyces cerevisiae*) YNO-0 strain to test the transient expression of NnCYP candidates, cloned in-frame with the triose-phosphate dehydrogenase promoter (TDH3, in blue) and the cytochrome c 1 terminator (CYC, in yellow).

pEV2 (circular vector sequence)

TGTTTTATATTTGTTGTAAAAAGTAGATAATTACTTCCTTGATGATCTGTAAAAAAGAGAAAAAGAAAG
CATCTAAGAACTTGAAAAACTACGAATTAGAAAAGACCAAATATGTATTTCTTGCATTGACCAATTTAT
GCAAGTTTATATATATGTAAATGTAAGTTTCACGAGGTTCTACTAAACTAAACCACCCCCTTGGTTAGA
AGAAAAGAGTGTGTGAGAACAGGCTGTTGTTGTCACACGATTCGGACAATTCTGTTTGAAAGAGAGA
GAGTAACAGTACGATCGAACGAACTTTGCTCTGGAGATCACAGTGGGCATCATAGCATGTGGTACTA
AACCCTTTCCCGCCATTCCAGAACCTTCGATTGCTTGTTACAAAACCTGTGAGCCGTCGCTAGGACC
TTGTTGTGTGACGAAATTGGAAGCTGCAATCAATAGGAAGACAGGAAGTCGAGCGTGTCTGGGTTTT
TTCAGTTTTGTTCTTTTTGCAAACAACAGTTTATTCCTGGCATCCACTAAATATAATGGAGCCCGCTTT
TTAAGCTGGCATCCAGAAAAAAAAAGAATCCCAGCACCAAAATATTGTTTTCTTCACCAACCATCAGT
TCATAGGTCCATTCTCTTAGCGCAACTACAGAGAACAGGGGCACAAACAGGCAAAAAACGGGCACA
ACCTCAATGGAGTGATGCAACCTGCCTGGAGTAAATGATGACACAAGGCAATTGACCCACGCATGTA
TCTATCTCATTTTCTTACACCTTCTATTACCTTCTGCTCTCTCTGATTTGGAAAAAGCTGAAAAAAAAG
GTTGAAACCAGTTCCCTGAAATTATTCCCCTACTTGACTAATAAGTATATAAAGACGGTAGGTATTGA
TTGTAATTCTGTAAATCTATTTCTTAAACTTCTTAAATTCTACTTTTATAGTTAGTCTTTTTTTTAGTTTTA
AAACACCAAGAACTTAGTTTCGAATAAACACACATAAACAAACAAAGGATCCACCAGCTTGGTACCGC
GGCTAGCTAAGATCCGCTCTAACCGAAAAGGAAGGAGTTAGACAACCTGAAGTCTAGGTCCCTATTT
ATTTTTTTATAGTTATGTTAGTATTAAGAACGTTATTTATATTTCAAATTTTTCTTTTTTTTCTGTACAGA
CGCGTGTACGCATGTAACATTATACTGAAAACCTTGCTTGAGAAGGTTTTGGGACGCTCGAAGATCC
AGCTGCATTAATGAATCGGCCAACGCGCGGGGAGAGGCGGTTTGCGTATTGGGCGCTCTTCCGCTT
CCTCGCTCACTGACTCGCTGCGCTCGGTCGTTCGGCTGCGGCGAGCGGTATCAGCTCACTCAAAGG
CGGTAATACGGTTATCCACAGAATCAGGGGATAACGCAGGAAAGAACATGTGAGCAAAAGGCCAGC
AAAAGGCCAGGAACCGTAAAAAGGCCGCGTTGCTGGCGTTTTTCCATAGGCTCCGCCCCCCTGACG
AGCATCACAAAAATCGACGCTCAAGTCAGAGGTGGCGAAACCCGACAGGACTATAAAGATACCAGG
CGTTTCCCCCTGGAAGCTCCCTCGTGCGCTCTCCTGTTCCGACCCTGCCGCTTACCGGATACCTGT
CCGCCTTTCTCCCTTCGGGAAGCGTGGCGCTTTCTCATAGCTCACGCTGTAGGTATCTCAGTTCGGT
GTAGGTCGTTCGCTCCAAGCTGGGCTGTGTGCACGAACCCCCCGTTCAGCCCGACCGCTGCGCCTT
ATCCGGTAACTATCGTCTTGAGTCCAACCCGGTAAGACACGACTTATCGCCACTGGCAGCAGCCACT
GGTAACAGGATTAGCAGAGCGAGGTATGTAGGCGGTGCTACAGAGTTCTTGAAGTGGTGGCCTAAC
TACGGCTACACTAGAAGAACAGTATTTGGTATCTGCGCTCTGCTGAAGCCAGTTACCTTCGGAAAAA
GAGTTGGTAGCTCTTGATCCGGCAAACAAACCACCGCTGGTAGCGGTGGTTTTTTTGTTTGCAAGCA
GCAGATTACGCGCAGAAAAAAAGGATCTCAAGAAGATCCTTTGATCTTTTCTACGGGGTCTGACGCT
CAGTGGAACGAAAACTCACGTTAAGGGATTTTGGTCATGAGATTATCAAAAAGGATCTTCACCTAGAT
CCTTTTAAATTAAAAATGAAGTTTTAAATCAATCTAAAGTATATATGAGTAAACTTGGTCTGACAGTTAC
CAATGCTTAATCAGTGAGGCACCTATCTCAGCGATCTGTCTATTTCGTTCATCCATAGTTGCCTGACT
CCCCGTCGTGTAGATAACTACGATACGGGAGGGCTTACCATCTGGCCCCAGTGCTGCAATGATACC
GCGAGACCCACGCTCACCGGCTCCAGATTTATCAGCAATAAACCAGCCAGCCGGAAGGGCCGAGC
GCAGAAGTGGTCCTGCAACTTTATCCGCCTCCATCCAGTCTATTAATTGTTGCCGGGAAGCTAGAGT
AAGTAGTTCGCCAGTTAATAGTTTGCGCAACGTTGTTGCCATTGCTACAGGCATCGTGGTGTCACGC
TCGTCGTTTGGTATGGCTTCATTCAGCTCCGGTTCCCAACGATCAAGGCGAGTTACATGATCCCCCA
TGTTGTGCAAAAAAGCGGTTAGCTCCTTCGGTCCTCCGATCGTTGTCAGAAGTAAGTTGGCCGCAGT
GTTATCACTCATGGTTATGGCAGCACTGCATAATTCTCTTACTGTCATGCCATCCGTAAGATGCTTTT
CTGTGACTGGTGAGTACTCAACCAAGTCATTCTGAGAATAGTGTATGCGGCGACCGAGTTGCTCTTG
CCCGGCGTCAATACGGGATAATACCGCGCCACATAGCAGAACTTTAAAAGTGCTCATCATTGGAAAA
CGTTCTTCGGGGCGAAAACTCTCAAGGATCTTACCGCTGTTGAGATCCAGTTCGATGTAACCCACTC
GTGCACCCAACTGATCTTCAGCATCTTTTACTTTCACCAGCGTTTCTGGGTGAGCAAAAACAGGAAG

GCAAAATGCCGCAAAAAAGGGAATAAGGGCGACACGGAAATGTTGAATACTCATACTCTTCCTTTTT
CAATATTATTGAAGCATTTATCAGGGTTATTGTCTCATGAGCGGATACATATTTGAATGTATTTAGAAA
AATAAACAAATAGGGGTTCCGCGCACATTTCCCCGAAAAGTGCCACCTGAACGAAGCATCTGTGCTT
CATTTTGTAGAACAAAAATGCAACGCGAGAGCGCTAATTTTTCAAACAAAGAATCTGAGCTGCATTTT
TACAGAACAGAAATGCAACGCGAAAGCGCTATTTTACCAACGAAGAATCTGTGCTTCATTTTTGTAAA
ACAAAAATGCAACGCGAGAGCGCTAATTTTTCAAACAAAGAATCTGAGCTGCATTTTTACAGAACAGA
AATGCAACGCGAGAGCGCTATTTTACCAACAAAGAATCTATACTTCTTTTTTGTTCTACAAAAATGCAT
CCCGAGAGCGCTATTTTTCTAACAAAGCATCTTAGATTACTTTTTTTCTCCTTTGTGCGCTCTATAATG
CAGTCTCTTGATAACTTTTTGCACTGTAGGTCCGTTAAGGTTAGAAGAAGGCTACTTTGGTGTCTATT
TTCTCTTCCATAAAAAAAGCCTGACTCCACTTCCCGCGTTTACTGATTACTAGCGAAGCTGCGGGTG
CATTTTTTCAAGATAAAGGCATCCCCGATTATATTCTATACCGATGTGGATTGCGCATACTTTGTGAA
CAGAAAGTGATAGCGTTGATGATTCTTCATTGGTCAGAAAATTATGAACGGTTTCTTCTATTTTGTCTC
TATATACTACGTATAGGAAATGTTTACATTTTCGTATTGTTTTCGATTCACTCTATGAATAGTTCTTACT
ACAATTTTTTTGTCTAAAGAGTAATACTAGAGATAAACATAAAAAATGTAGAGGTCGAGTTTAGATGCA
AGTTCAAGGAGCGAAAGGTGGATGGGTAGGTTATATAGGGATATAGCACAGAGATATATAGCAAAGA
GATACTTTTGAGCAATGTTTGTGGAAGCGGTATTCGCAATATTTTAGTAGCTCGTTACAGTCCGGTGC
GTTTTTGGTTTTTTGAAAGTGCGTCTTCAGAGCGCTTTTGGTTTTCAAAAGCGCTCTGAAGTTCCTAT
ACTTTCTAGAGAATAGGAACTTCGGAATAGGAACTTCAAAGCGTTTCCGAAAACGAGCGCTTCCGAA
AATGCAACGCGAGCTGCGCACATACAGCTCACTGTTCACGTCGCACCTATATCTGCGTGTTGCCTGT
ATATATATATACATGAGAAGAACGGCATAGTGCGTGTTTATGCTTAAATGCGTACTTATATGCGTCTAT
TTATGTAGGATGAAAGGTAGTCTAGTACCTCCTGTGATATTATCCCATTCCATGCGGGGTATCGTATG
CTTCCTTCAGCACTACCCTTTAGCTGTTCTATATGCTGCCACTCCTCAATTGGATTAGTCTCATCCTTC
AATGCTATCATTTCCTTTGATATTGGATCATACTAAGAAACCATTATTATCATGACATTAACCTATAAAA
ATAGGCGTATCACGAGGCCCTTTCGTCTCGCGCGTTTCGGTGATGACGGTGAAAACCTCTGACACAT
GCAGCTCCCGGAGACGGTCACAGCTTGTCTGTAAGCGGATGCCGGGAGCAGACAAGCCCGTCAGG
GCGCGTCAGCGGGTGTTGGCGGGTGTCGGGGCTGGCTTAACTATGCGGCATCAGAGCAGATTGTA
CTGAGAGTGCACCATATTTCACACCGCATAGATCCGTCGAGTTCAAGAGAAAAAAAAAGAAAAAGCA
AAAAGAAAAAAGGAAAGCGCGCCTCGTTCAGAATGACACGTATAGAATGATGCATTACCTTGTCATC
TTCAGTATCATACTGTTCGTATACATACTTACTGACATTCATAGGTATACATATATACACATGTATATAT
ATCGTATGCTGCAGCTTTAAATAATCGGTGTCACTACATAAGAACACCTTTGGTGGAGGGAACATCG
TTGGTACCATTGGGCGAGGTGGCTTCTCTTATGGCAACCGCAAGAGCCTTGAACGCACTCTCACTAC
GGTGATGATCATTCTTGCCTCGCAGACAATCAACGTGGAGGGTAATTCTGCTAGCCTCTGCAAAGCT
TTCAAGAAAATGCGGGATCATCTCGCAAGAGAGATCTCCTACTTTCTCCCTTTGCAAACCAAGTTCGA
CAACTGCGTACGGCCTGTTCGAAAGATCTACCACCGCTCTGGAAAGTGCCTCATCCAAAGGCGCAA
ATCCTGATCCAAACCTTTTTACTCCACGCACGGCCCCTAGGGCCTCTTTAAAAGCTTGACCGAGAGC
AATCCCGCAGTCTTCAGTGGTGTGATGGTCGTCTATGTGTAAGTCACCAATGCACTCAACGATTAGC
GACCAGCCGGAATGCTTGGCCAGAGCATGTATCATATGGTCCAGAAACCCTATACCTGTGTGGACG
TTAATCACTTGCGATTGTGTGGCCTGTTCTGCTACTGCTTCTGCCTCTTTTTCTGGGAAGATCGAGTG
CTCTATCGCTAGGGGACCACCCTTTAAAGAGATCGCAATCTGAATCTTGGTTTCATTTGTAATACGCT
TTACTAGGGCTTTCTGCTCTGTCATCTTTGCCTTCGTTTATCTTGCCTGCTCATTTTTTAGTATATTCTT
CGAAGAAATCACATTACTTTATATAATGTATAATTCATTATGTGATAATGCCAATCGCTAAGAAAAAAA
AAGAGTCATCCGCTAGGTGGAAAAAAAAAAAATGAAAATCATTACCGAGGCATAAAAAAATATAGAGTG
TACTAGAGGAGGCCAAGAGTAATAGAAAAAGAAAATTGCGGGAAAGGACTGTGTTATGACTTCCCTG
ACTAATGCCGTGTTCAAACGATACCTGGCAGTGACTCCTAGCGCTCACCAAGCTCTTAAAACGGGAA
TTTATGGTGCACTCTCAGTACACGCGCCAGATCTGTTTAGCTTGCCTCGTCCCCGCCGGGTCACCC
GGCCAGCGACATGGAGGCCCAGAATACCCTCCTTGACAGTCTTGACGTGCGCAGCTCAGGGGCAT
GATGTGACTGTCGCCCGTACATTTAGCCCATACATCCCCATGTATAATCATTTGCATCCATACATTTT
GATGGCCGCACGGCGCGAAGCAAAAATTACGGCTCCTCGCTGCAGACCTGCGAGCAGGGAAACGC
TCCCCTCACAGACGCGTTGAATTGTCCCCACGCCGCGCCCCTGTAGAGAAATATAAAAGGTTAGGAT
TTGCCACTGAGGTTCTTCTTTCATATACTTCCTTTTAAAATCTTGCTAGGATACAGTTCTCACATCACA
TCCGAACATAAACAACCATGGGTAAGGAAAAGACTCACGTTTCGAGGCCGCGATTAAATTCCAACAT
GGATGCTGATTTATATGGGTATAAATGGGCTCGCGATAATGTCGGGCAATCAGGTGCGACAATCTAT

Supplementary Information

CGATTGTATGGGAAGCCCGATGCGCCAGAGTTGTTTCTGAAACATGGCAAAGGTAGCGTTGCCAAT
GATGTTACAGATGAGATGGTCAGACTAAACTGGCTGACGGAATTTATGCCTCTTCCGACCATCAAGC
ATTTTATCCGTACTCCTGATGATGCATGGTTACTCACCACTGCGATCCCCGGCAAAACAGCATTCCA
GGTATTAGAAGAATATCCTGATTCAGGTGAAAATATTGTTGATGCGCTGGCAGTGTTCCTGCGCCGG
TTGCATTCGATTCCTGTTTGTAATTGTCCTTTTAACAGCGATCGCGTATTTCGTCTCGCTCAGGCGCA
ATCACGAATGAATAACGGTTTGGTTGATGCGAGTGATTTTGATGACGAGCGTAATGGCTGGCCTGTT
GAACAAGTCTGGAAAGAAATGCATAAGCTTTTGCCATTCTCACCGGATTCAGTCGTCACTCATGGTG
ATTTCTCACTTGATAACCTTATTTTTGACGAGGGGAAATTAATAGGTTGTATTGATGTTGGACGAGTC
GGAATCGCAGACCGATACCAGGATCTTGCCATCCTATGGAACTGCCTCGGTGAGTTTTCTCCTTCAT
TACAGAAACGGCTTTTTCAAAAATATGGTATTGATAATCCTGATATGAATAAATTGCAGTTTCATTTGA
TGCTCGATGAGTTTTTCTAATCAGTACTGACAATAAAAAGATTCTTGTTTTCAAGAACTTGTCATTTGT
ATAGTTTTTTTATATTGTAGTTGTTCTATTTTAATCAAATGTTAGCGTGATTTATATTTTTTTTCGCCTCG
ACATCATCTGCCCAGATGCGAAGTTAAGTGCGCAGAAAGTAATATCATGCGTCAATCGTATGTGAAT
GCTGGTCGCTATACTGCTGTCGATTCGATACTAACGCCGCCATCCAGTGTCGAATTCGCCATTCAGG
CTGCGCAACTGTTGGGAAGGGCGATCGGTGCGGGCCTCTTCGCTATTACGCCAGCTGAATTGGAGC
GACCTCATGCTATACCTGAGAAAGCAACCTGACCTACAGGAAAGAGTTACTCAAGAATAAGAATTTTC
GTTTTAAAACCTAAGAGTCACTTTAAAATTTGTATACACTTATTTTTTTTATAACTTATTTAATAATAAAA
ATCATAAATCATAAGAAATTCGCTTATTTAGAAGTGTCAACAACGTATCTACCAACGATTTGACCCTTT
TCCATCTTTTCGTAAATTTCTGGCAAGGTAGACAAGCCGACAACCTTGATTGGAGACTTGACCAAACC
TCTGGCGAAGAATTGTTAATTAAGAGCTCAGATCTTTTGCGGCCGC

Supplementary References

1 Mizutani, M. & Sato, F. Unusual P450 reactions in plant secondary metabolism. *Arch Biochem Biophys* **507**, 194-203 (2011).

2 Stuart, K. L. & Cava, M. P. Proaporphine alkaloids. *Chem. Rev.* **68**, 321-339 (1968).

3 Stadler, R. & Zenk, M. H. The purification and characterization of a unique cytochrome P-450 enzyme from *Berberis stolonifera* plant cell cultures. *J Biol Chem* **268**, 823-831 (1993).

4 Bauer, W. & Zenk, M. H. Two methylenedioxy bridge forming cytochrome P-450 dependent enzymes are involved in (*S*)-stylopine biosynthesis. *Phytochemistry* **30**, 2953-2961 (1991).

5 Zhang, L. *et al.* Direct coupling of thin-layer chromatography-bioautography with electrostatic field induced spray ionization-mass spectrometry for separation and identification of lipase inhibitors in lotus leaves. *Anal Chim Acta* **967**, 52-58 (2017).

6 Menendez-Perdomo, I. M. & Facchini, P. J. Isolation and characterization of two *O*methyltransferases involved in benzylisoquinoline alkaloid biosynthesis in sacred lotus (Nelumbo nucifera). *J Biol Chem* **295**, 1598-1612 (2020).

7 Desgagne-Penix, I. & Facchini, P. J. Systematic silencing of benzylisoquinoline alkaloid biosynthetic genes reveals the major route to papaverine in opium poppy. *Plant J* **72**, 331-344 (2012).

8 Guo, Y., Chen, X., Qi, J. & Yu, B. Simultaneous qualitative and quantitative analysis of flavonoids and alkaloids from the leaves of *Nelumbo nucifera* Gaertn. using high-performance liquid chromatography with quadrupole time-of-flight mass spectrometry. *J Sep Sci* **39**, 2499-2507 (2016).

9 Deng, X. *et al.* Analysis of isoquinoline alkaloid composition and wound-induced variation in *Nelumbo* using HPLC-MS/MS. *J Agric Food Chem* **64**, 1130-1136 (2016).

10 Luo, X., Chen, B., Liu, J. & Yao, S. Simultaneous analysis of *N*-nornuciferine, *O*-nornuciferine, nuciferine, and roemerine in leaves of *Nelumbo nucifera* Gaertn by high-performance liquid chromatography–photodiode array detection–electrospray mass spectrometry. *Analytica Chimica Acta* **538**, 129-133 (2005).

11 Lin, Z., Yang, R., Guan, Z., Chen, A. & Li, W. Ultra-performance LC separation and quadrupole time-of-flight MS identification of major alkaloids in Plumula Nelumbinis. *Phytochem Anal* **25**, 485-494 (2014).

12 Li, J., Lee, E. J., Chang, L. & Facchini, P. J. Genes encoding norcoclaurine synthase occur as tandem fusions in the Papaveraceae. *Sci Rep* **6**, 39256 (2016).

13 Hagel, J. M. *et al.* Transcriptome analysis of 20 taxonomically related benzylisoquinoline alkaloid-producing plants. *BMC Plant Biol* **15**, 227 (2015).

14 Liu, X. *et al.* Functional characterization of (*S*)-*N*-methylcoclaurine 3'-hydroxylase (NMCH) involved in the biosynthesis of benzylisoquinoline alkaloids in *Corydalis yanhusuo*. *Plant Physiol Biochem* **168**, 507-515 (2021).
